# Tuning the Photocatalytic Activity of Ti-Based Metal–Organic Frameworks through Modulator Defect-Engineered Functionalization

**DOI:** 10.1021/acsami.2c02668

**Published:** 2022-04-28

**Authors:** Isabel Abánades Lázaro, Horatiu Szalad, Pablo Valiente, Josep Albero, Hermenegildo García, Carlos Martí-Gastaldo

**Affiliations:** † Instituto de Ciencia Molecular (ICMol), Universitat de València, Catedrático José Beltrán Martínez no 2, 46980 Paterna, València, Spain; ‡ Instituto Universitario de Tecnología Química CSIC-UPV, UniversitatPolitècnica de València, Av. De los Naranjos s/n, 46022 València, Spain

**Keywords:** metal−organic frameworks, defects, porous materials, functionalized materials, photocatalysis, photostability

## Abstract

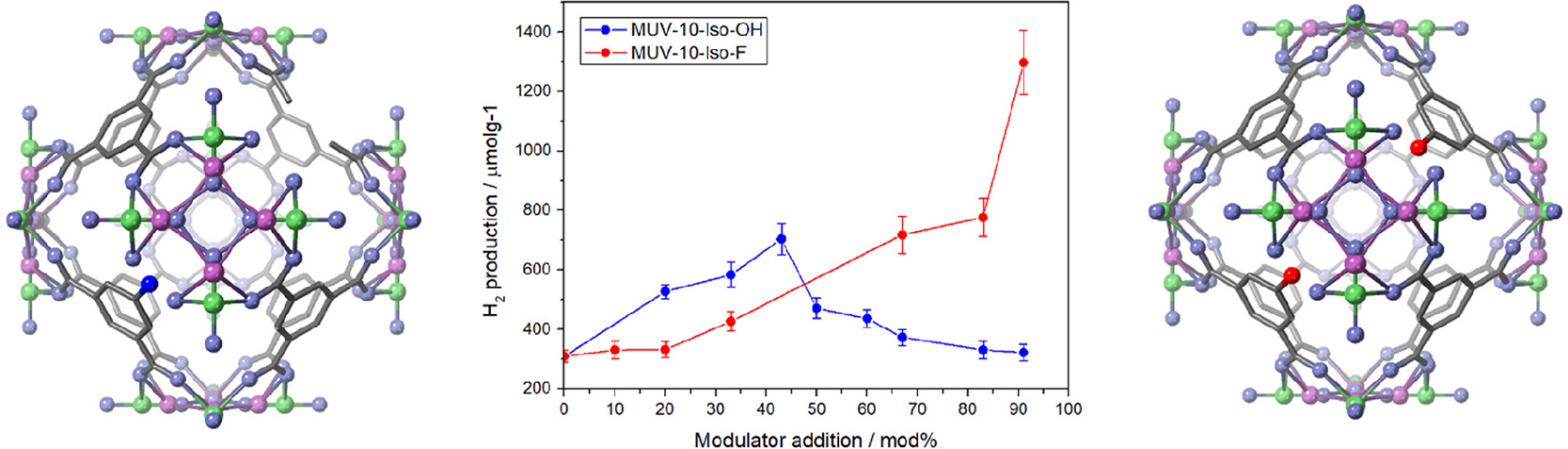

Defect engineering is a valuable tool to tune the photocatalytic activity of metal–organic frameworks (MOFs). Inducing defects through the attachment of functionalized modulators can introduce cooperative units that can tune the bandgap of the material and enhance their chemical, thermal, and photostabilities among other properties. However, the majority of defect engineering studies for photocatalytic applications are limited to Zr-based MOFs, and there is still a lack of interrelation between synthetic variables, the resultant MOF properties, and their effect on their photocatalytic performance. We report a comprehensive study on the defect engineering of the titanium heterometallic MOF MUV-10 by fluoro- and hydroxy-isophthalic acid (Iso) modulators, rationalizing the effect of the materials’ properties on their photocatalytic activity for hydrogen production. The Iso-OH modified MOFs present a volcano-type profile with a 2.3-fold increase in comparison to the pristine materials, whereas the Iso-F modified samples have a gradual increase with up to a 4.2-fold enhancement. It has been demonstrated that ∼9% of Iso-OH modulator incorporation produces ∼40% defects, inducing band gap reduction and longer excited states lifetime. Similar defect percentages have been generated upon near 40% Iso-F modulator incorporation; however, negligible band gap changes and shorter excited states lifetimes were determined. The higher photocatalytic activity in Iso-F modulator derived MOF has been attributed to the effect of the divergent defect-compensation modes on the materials’ photostability and to the increase in the external surface area upon introduction of Iso-F modulator.

## Introduction

The increasing energy demand together with the environmental concerns derived from the massive consumption of fossil fuels has stimulated the research for the obtention of environmentally friendly fuels and chemicals from clean and renewable energy sources.^1^ In this regard, photocatalysis is an appealing approach to directly convert the abundant and renewable sunlight energy into fuels and chemicals by means of a photocatalyst.^2,3^ Since the seminal paper of Fujishima and Honda,^4^ a wide range of semiconductor materials headed by TiO_2_, among other metal oxides, phosphides, and nitrides, as well as carbon-based materials (doped-graphene, g-C_3_N_4_, etc.), have been investigated as photocatalysts for light-driven reactions such as water splitting, CO_2_ reduction and N_2_ fixation, among others.^3,5−7^

Recently, metal–organic frameworks (MOFs),^8,9^ a class of crystalline porous solids composed of metal ions or clusters connected by organic linkers, have attracted much attention in photocatalysis as a consequence of their large specific surface area, flexible design of micro- or mesoporous structures, and the possibility to incorporate different organic moieties or metal clusters.^10−14^ Interestingly, the rational design of the organic linkers can endow very promising photophysical and photochemical properties since it can extend the light-harvesting in the visible range,^15,16^ modulate substrates adsorption due to Brönsted acid or basic sites,^17^ provide additional binding centers,^18,19^ modulate charge separation and recombination,^13^ or even alter the MOF stability.^20,21^

However, most of the reported functionalized linkers aim for the photoresponse enhancement, while other factors, such as the stabilization of adequate intermediate species, structural stability during reaction or charge carrier recombination suppression, could also increase the efficiency of MOFs as photocatalysts.^10−12,15^ Thus, for instance, it has been reported that amino functionalized linkers not only enhance light harvesting in the visible range but also influence the excited states decay profile and location in MIL-125(Ti).^22^ It was demonstrated that upon NH_2_-MIL-125(Ti) photoexcitation, the electrons are located in the Ti-oxo clusters, while the holes remain at the NH_2_-functionalized linkers. This rendered in long-lived charge carriers, suppressing charge recombination. Similarly, mixed-linker UiO-66 MOF using a 1:1 molar ratio of 2-amino-1,4-benzenedicarboxylic acid and (2-X-1,4-bezenedicarboxylate (X-BDC, X = H, F, Cl, Br)) enhanced the photocatalytic activity of this MOF toward the benzyl alcohol oxidation reaction. The halogenated ligands promoted ^•^O_2_^–^ radicals stabilization on Zr^3+^, which could decrease the recombination rate and therefore increase the reaction yield.^23^

Among protocols to functionalize MOFs,^15,24,25^ coordination modulation (CM) has recently acquired a notable interest. Coordination modulation is a synthetic tool based on the introduction of ligands (modulators) that compete with the linkers for metal complexation during MOF solvothermal synthesis,^26,27^ and it is used to fine-tune MOFs’ pore functionalization^28,29^ among other properties such as crystallinity,^30^ particle size,^30,31^ defectivity,^32−34^ chemical reactivity,^34^ and porosity.^33^ Because of DMF decomposition to formic acid, coordination modulation occurs in any DMF containing synthesis.^33^ However, the complex equilibria that govern MOFs’ self-assembly are still not fully understood due to the complexity of the underlying deprotonation, metal-complexation, nucleation and crystallization equilibria, and the lack of inter-relation between synthetic variables and properties.^26,35,36^ Moreover, defects, which can be induced through the introduction of functionalized modulators, have a direct implication in the photophysical, photochemical, and structural properties of MOFs,^12,37−39^ modulating the band gap^37^ and electronic structures,^40,41^ and the conductivity,^42,43^ active sites,^17,34^ and MOFs stability. In this regard, theoretical studies show that missing linkers in UiO-66 result in a decrease of the energy levels of the unoccupied d orbitals of Zr atoms, with a possible increase in charge transfer in the photocatalytic process.^40,41^ Moreover, the coordinatively unsaturated sites associated with surface defects can act as active sites for substrate activation. However, studies that directly relate the defect chemistry of defect-engineered functionalized materials with their photocatalytic performance are mostly related to Zr based MOFs of the UiO topology.

**Figure 1:**
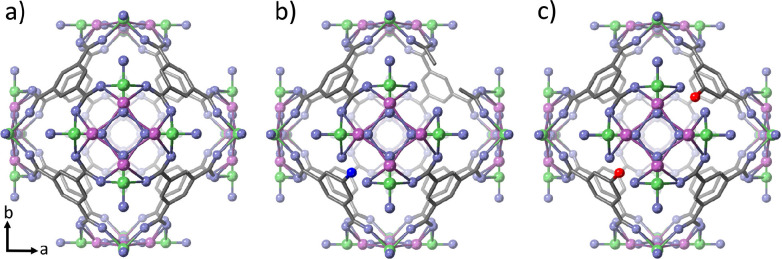
Schematic representation of (a) MUV-10 and MUV-10 modified with (b) 5-hydroxy isophthalic acid (blue) and (c) 5-fluoro isophthalic acid (dark red). Green titanium, purple calcium, gray carbon, blue oxygen and red fluorine. Hydrogen atoms have been omitted for clarity.

Herein, we report the comprehensive modification of MUV-10 (Figure 1a), an 8,3-connected heterometallic Ti (IV)-MOF that is built of Ti^IV^_2_Ca^II^_2_(μ_3_-O)_2_(H_2_O)_4_(RCO_2_)_8_ clusters connected by benzene tricarboxylate (btc) linkers forming a cubic structure with unit cell [Ti_3_Ca_3_(μ_3_-O)_3_(μ_2_-C_6_H_3_(CO_2_)_3_)_4_(OH_2_)_6_],^44^ with defect-inducing 5-hydroxy isophthalic acid and 5-fluoro isophthalic acid modulators at different ratios, and we have correlated the compositional and photophysical properties of the resultant materials with their photocatalytic activity for H_2_ evolution, achieving up to a 4.2-fold and 2.3-fold increase in H_2_ production compared to the pristine material using the 5-fluoro isophthalic acid and 5-hydroxy isophthalic modulators, respectively. MUV-10 is a highly stable (500 °C) porous framework able to tolerate a high degree of defects (up to 40 molar%, 3 missing linkers out of 8 in the unit cell) while maintaining its thermal and chemical stabilities.^45,46^ MUV-10 is stable in water from pH 2 to 12^44^ and is amenable to the uniform incorporation of modulators in its structure as defect-compensating ligands,^45^ which makes this Ti-containing MOF an ideal candidate to study the effect of introducing different functionalized modulators during its synthesis on the photocatalytic performance for H_2_ evolution.

## Results and Discussion

MUV-10, represented in Figure 1a, was synthesized by reacting one equivalent of CaCl_2_ and titanium(IV) isopropoxide with 1.5 equiv of btc linker in DMF containing AcOH in ∼18 v/v %, a synthesis that resulted in defect-free crystals of ∼2 μm with a BET surface area of 1040 m^2^ g^–1^.^44,45^

To introduce functionality into the frameworks through defect engineering, we have prepared MUV-10 containing 5-hydroxy isophthalic acid (Iso-OH) and 5-fluoro isophthalic acid (Iso-F) modulators, as represented in Figure 1b and c, aiming to enhance light harvesting in the visible range and to stabilize the photoexcited states to provide long-lived photocarriers with lower recombination rates. The functionalized MOFs are denominated MUV-10-Iso-OH(X) and MUV-10-Iso-F(X), respectively, where X corresponds to modulator percentage added during synthesis in comparison to the total amount of ligands in the synthesis (linker plus modulator).^45^

A fixed excess of linker (1.5 equiv compared to metal) was used in the synthesis of all MOFs as we have previously observed that these synthetic conditions are optimum for the generation of highly defective phases.^45^ The equivalents of each modulator in comparison to the linker, and the % of modulator added are presented in Table S1, showing the wide range of synthetic conditions employed during this study, from 0.25 equiv to 10 equiv, corresponding to 10 and 91% modulator addition, respectively (see Table S1 for detailed synthetic conditions and modulator addition in equivalents compared to the linker).

**Figure 2:**
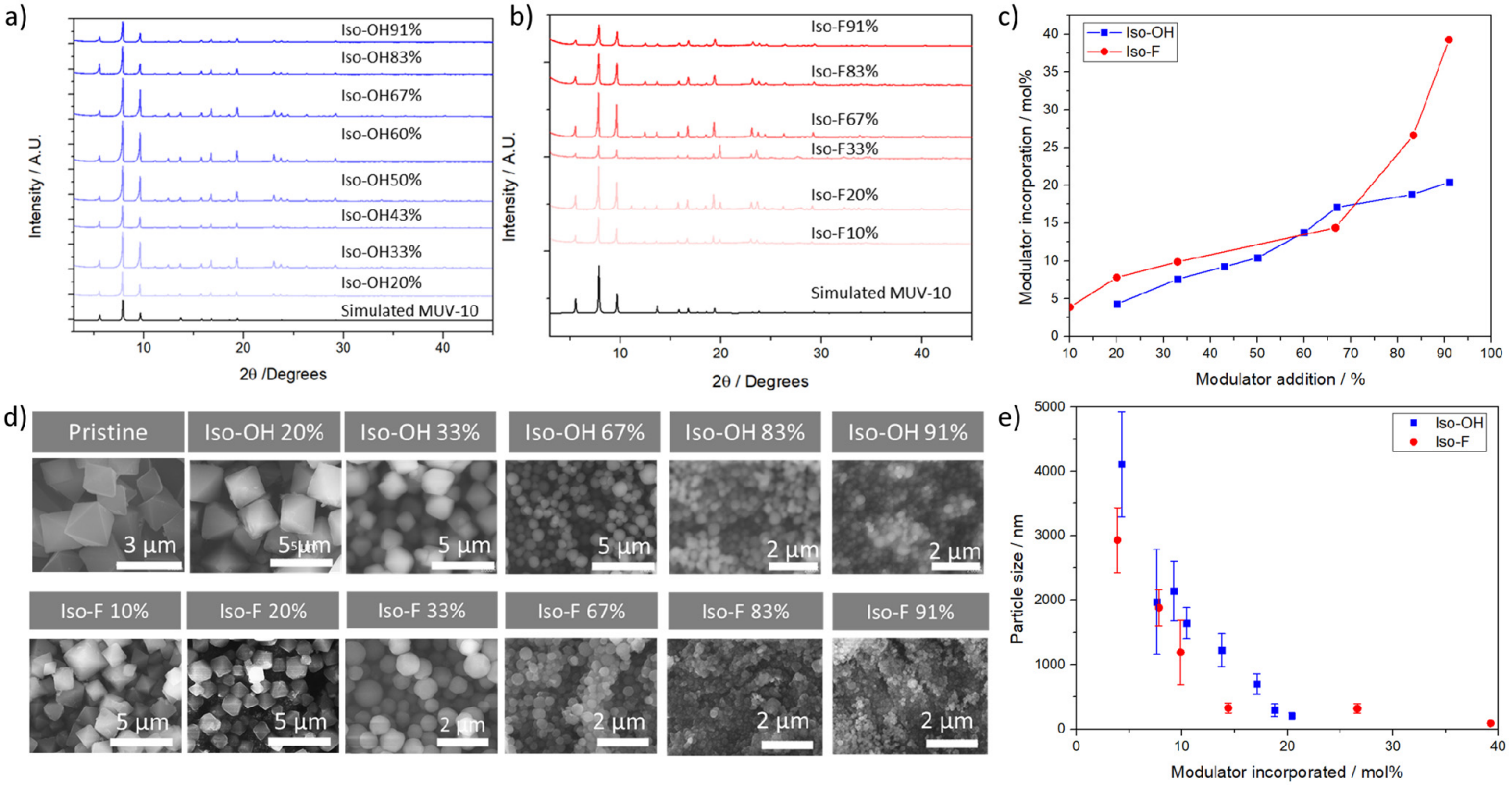
PXRD patterns of (a) Iso-OH modulated samples and (b) Iso-F modulated samples. (c) Modulator incorporation in molar percent as a function of modulator addition. (d) SEM images. (e) Particle size as a function of modulator incorporation in molar percent.

All the samples were highly crystalline and phase pure as evidenced in their PXRD profiles (Figure 2a,b), with Bragg diffraction bands confirming phase pure MUV-10 (see Figures S1 and S2 for PXRD profiles). All the samples had Ti:Ca ratios according to the 1:1 cluster structure, as determined by energy disperse X-ray (see Tables S2 and S3 for tabulated values). Analytical data, together with PXRD profiles, provide firm proof that there is no coformation of a different phase. While acid-digested ^1^HNMR showed high modulator’s incorporation (see section S.3.4), FT-IR confirmed the successful modulators attachment, as no free carboxylate units were observed in any case, confirming the interaction of the aromatic carboxylates with the metal clusters (see Figures S3–S8).

The modified MOFs displayed typical MUV-10 FT-IR profiles, with characteristic vibration bands coming from the functionalized modulators increasing in intensity along with their increasing percentage of addition, and subsequent incorporation such as the O–H *st* (ca. 3250 cm^–1^) and the O–H *ip* (ca. 1400 cm^–1^) for the Iso-OH modulator and the C–F *st* (ca. 950 cm^–1^) for the Iso-F modulator. Broadening of MUV-10 characteristic carboxylate vibration bands was observed as a consequence of defectivity (see Figures S3–S8 for FT-IR profiles and vibration bands’ assignation).

The modulator incorporation into the structure was quantified by acid-digested ^1^HNMR spectroscopy as molar percent and molar ratio (see Figures S9–S14 and Tables S4 and S5). During CM, the introduction of modulators influences equilibria,^31^ being their disruption is more noticeable upon increasing modulator acidity, which typically displays a higher degree of incorporation.^33^ Thus, the modulator incorporation was in good agreement with their acidity (Table 1 and Figure 2c). For both modulators, incorporation increases with the percentage added in the synthesis, linearly in the case of Iso-OH modulation across all the samples (*r*^2^ = 0.9899), while linearly upon low Iso-F addition (10–67%, *r*^2^ = 0.9705) and exponentially onward (Figure 2c). The Iso-F modulator is more acidic (p*K*_a_ 3.23) than the Iso-OH analogue (p*K*_a_ 3.32), and thus, the former is incorporated more significantly, from ∼4 mol % to ∼39 mol % incorporation, for MUV-10-Iso-F(10) and (91), respectively, double the incorporation of TFA in UiO-66 for similar modulator addition.^33^

**Table 1:** Modulator Incorporation in Molar Percent Determined by Quantitative ^1^HNMR Spectroscopy, Particle Size in nm Determined by SEM, BTC/Ti Ratio, and Modulator Per Missing Linker Determined by Combination of TGA, ^1^H-NMR Spectroscopy, and Porosimetry Values Extracted from N_2_ Adsorption/Desorption Isotherms Including BET, Micropore and External Surface Areas (m^2^ g^–1^), Micropore, Mesopore and Total Pore Volumes (cm^3^ g^–1^ Å^–1^), and Percent of External Surface Area

sample	Iso-X% Mol%	size ± SD	ratio btc/Ti	Mod/ML	*S*_BET_	*S*_MICRO_	*S*_EXT_	*V*_MICRO_	*V*_MESO_	*V*_TOTAL_	% *S*_EXT_
Pristine 2 μm	0	2359 ± 909	1.434	0.00	1040	974	66	0.365	0.037	0.402	6.3
Iso-OH 20%	4.286	4111 ± 821	0.918	0.237	1075	1002	73	0.383	0.031	0.414	6.8
Iso-OH 33%	7.595	1975 ± 811	0.988	0.283	1072	954	118	0.369	0.063	0.432	11.0
Iso-OH 43%	9.231	2144 ± 462	0.839	0.193	1083	957	81	0.371	0.064	0.435	7.5
Iso-OH 50%	10.420	1644 ± 240	0.656	0.156	1091	987	104	0.379	0.059	0.438	9.5
Iso-OH 60%	13.761	1226 ± 254	0.659	0.100	1152	1033	119	0.398	0.065	0.463	10.3
Iso-OH 67%	17.094	700 ± 157	0.779	0.174	1135	975	160	0.379	0.099	0.478	14.1
Iso-OH 83%	18.785	297 ± 97	0.600	0.113	1290	1129	161	0.4314	0.105	0.536	12.5
Iso-OH 91%	20.408	204 ± 53	0.473	0.157	1472	1348	124	0.524	0.097	0.621	8.4
Iso-F 10%	3.880	2931 ± 502	1.168	0.289	876	786	90	0.300	0.06	0.360	10.3
Iso-F 20%	7.806	1882 ± 280	1.116	0.443	910	789	121	0.304	0.087	0.391	13.3
Iso-F 33%	9.873	1193 ± 501	1.086	0.487	1051	930	121	0.356	0.072	0.428	11.5
Iso-F 67%	14.388	330 ± 76	1.082	0.734	958	803	155	0.313	0.114	0.427	16.2
Iso-F 83%	26.619	321 ± 72	0.961	0.944	929	710	219	0.282	0.228	0.510	23.6
Iso-F 91%	39.267	91 ± 27	0.809	1.005	1005	773	232	0.305	0.219	0.524	23.1

In the case of the Iso-OH modulator, incorporation in the MUV–OH(X) ranged from ∼4 mol % to ∼20 mol % for MUV-10-Iso-OH(20) and (91), respectively (Table 1).

The particle size and morphology of the different samples were investigated by scanning electron microscopy (SEM) (see Figures S15–S22 and Tables S7 and S8). Pristine MUV-10 is constituted by ∼2 μm crystals with octahedral morphology, while upon the introduction of modulators, the particle size is gradually reduced (Table 1), and morphology gradually changed to round nanoparticles for both modulators as shown in Figure 2d (see section S.3.5 for statistical analysis of particle size for all the samples).

The effect on the materials particle size aligns with the modulators acidity and is related to their incorporation, suggesting a capping effect (Figure 2e).^26,31^ However, the degree of incorporation is too high to be only located at the particle surface. The effect of Iso-OH in particle size is linearly related to its addition and incorporation (*r*^2^ = 0.96999), as represented in Figure 2e. Low addition of Iso-OH modulator results in octahedral crystals of ∼4111 ± 821 nm, 1975 ± 811 nm, and 2144 ± 462 nm for MUV-10-Iso-OH(20), (33), and (43), respectively, whereas addition higher amounts of modulator result in a gradual reduction of particle size down to 700 ± 157 nm, 297 ± 97 nm, and 205 ± 53 nm for MUV-10-Iso-OH(67), (83), and (91), respectively, with particle edges completely lost upon 67% addition. Low addition of Iso-F results in octahedral microcrystals of 2931 ± 502 nm, 1882 ± 280 nm, and 1193 ± 501 nm for 10%, 20%, and 33% of Iso-F modulator addition that gradually soften the edges to round particles. Further Iso-F addition promotes size decrease up to 330 ± 76 nm, 321 ± 72 nm, and 91 ± 27 nm upon 67%, 83%, and 91% modulator addition, respectively. Similarly to the incorporation degree analyzed by ^1^HNMR, the effect on particle size is linearly related for low degrees of addition and incorporation (up to Iso-F67%, 15 mol %, *r*^2^ = 0.998), as represented in Figure 2e. Energy-dispersed X-ray mapping (see Figures S23–S25) confirmed the homogeneous distribution of the modulators’ signature elements within the particles.

**Figure 3:**
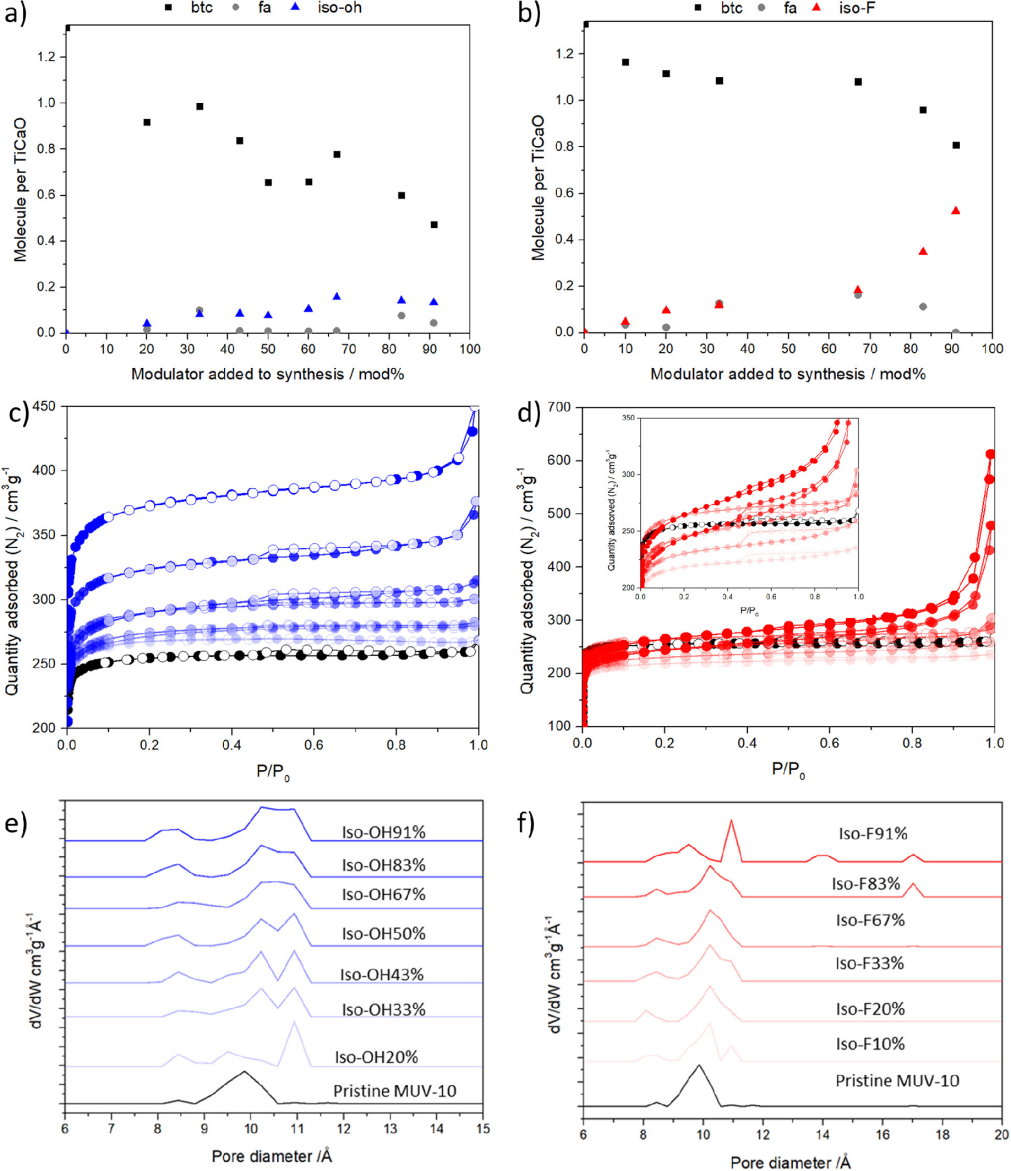
MOF compositional analysis, linker (btc) formic acid (fa) and Iso-X modulator per unit formula for (a) Iso-OH modulated samples and (b) Iso-F modulated samples. N_2_ adsorption and desorption isotherms of (c) Iso-OH modulated samples and (d) Iso-F modulated samples. Pore size distribution of (e) Iso-OH modulated samples and (f) Iso-F modulated samples. Note that as the color darkness the modulator addition increases in panels c and d as in e and f. Black sample corresponding to pristine MUV-10 (2 μm).

To further investigate the composition of the materials and the effect of modulators in defect compensation, we applied a multifaceted method based on the combination of ^1^HNMR spectroscopy with TGA (see section S.3.6 for models and mathematical determination, Figures S26–S37).^47^ All the modulated samples display defectivity that arises from the incorporation of modulators as defect compensating ligands, being the molar percent of missing linkers related to modulators addition and subsequent incorporation (Tables S9 and S10). However, although the incorporation of Iso-OH modulator is less significant than Iso-F for analogue synthetic conditions, this induces a higher degree of defects, reaching up to a ∼65 mol % of linker deficiency upon the highest addition, whereas a maximum of ∼40 mol % of linker deficiency is reached upon F-modulation for the same conditions, which highlights the complexity of the coordination modulation process. Figure 3a and b show the linker to metal ratio and the modulator and formic acid per unit formula upon Iso-OH and Iso-F modulation, respectively, which highlights the differences in defect-compensation between the set. The linker to metal ratio and the modulator per missing linker are tabulated in Table 1, (see Tables S9 and S10 for molecular formulas, molar percent of linker deficiency and missing linkers per unit cell). MUV-10-Iso-OH defectivity is not fully compensated by Iso-OH and FA modulators. In fact, across the Iso-OH modulated series the MOF displays between ∼0.3–0.1 modulators per missing linker defect, while in the Iso-F modulated series the samples display ∼0.3–1 modulators per missing linker, gradually increasing with defectivity, as shown in Table 1, meaning that the most defective MUV-10-Iso-F samples are compensated by Iso-F modulator attachment and that Iso-F is more efficient in compensating the induced defectivity than the analogue Iso-OH. Thus, MUV-10-Iso-OH samples have more defects compensated by OH/H_2_O pairs (ca. 1–2 pairs per molecular formula) than Iso-F modulated samples (ca. 0.25–1 pairs per unit formula) (see Tables S9 and S10).

These differences in defect compensation have a direct impact on the materials thermal and chemical stabilities (see sections S.3.6 and S.3.7), with MUV-10-Iso-OH having a gradual decrease in thermal stability (from ca. 500 °C to ca. 390 °C) (Figure S28) that is directly related to the OH-modulator incorporation (Figure S29), whereas MUV-10-Iso-F is thermally stable across all the series (Figure S34). It is important to note that a decrease in thermal stability upon the introduction of defect-compensating modulators has been observed before.^33^ OH moieties are monodentate and coordinate only to one metal position, whereas having missing linker defects compensated by ditopic isophthalic acid modulators results in 4 coordination sites that connect metals and clusters, thus providing higher stability to the framework.^21^ In consonance, the Iso-OH modulated samples display a decrease in stability toward dispersion in water during 24 h, as envisioned in the decrease in crystallinity (Figure S38) and damage to the crystals observed by SEM (Figure S39) that increases with the modulator incorporation and subsequently induced defects, while the Iso-F modulated samples are stable under the same conditions, with identical PXRD profiles (Figure S40) and no damage to the crystals (Figure S41). It is also important to take into account that the hydrophobicity of the Iso-F modulator could prevent the crystals from water instability.

The differences in defect compensation could also be one of the reasons why the samples display significant differences in porosity,^21^ tabulated in Table 1 (see Figure S42–S51 and Tables S11 and S12 for a detailed assessment of the material’s porosity). MUV-10 is a microporous framework, with a surface area of close to 1000 m^2^ g^–1^, a pore centered at ∼1 nm, and ∼0.40 cm^3^ g^–1^ pore volume.^44,45^ MUV-10-Iso-OH displays type I isotherms (Figure 3c) with an exponential increase in porosity with modulators incorporation up to *S*_BET_ = 1472 m^2^ g^–1^ for Iso-OH91%, with a pore volume of ∼0.621 cm^3^ g^–1^.

In contrast, a gradual shift toward type IV isotherms is observed upon Iso-F modulation in Figure 3d, showing hysteresis loops characteristic of mesoporous materials and interparticle space for MUV-10-Iso-F(83) and (91) samples that exhibit the smaller particle sizes. In this case, the BET surface areas are maintained or slightly reduced compared to pristine MUV-10 (876–1051 m^2^ g^–1^), whereas a significant increase in the samples mesoporosity is observed, reaching pore volumes of ∼0.524 cm^3^ g^–1^ at *P*/*P*_0_ = 0.9 for the Iso-F91% sample, before the contribution of interparticle space, reaching a pore volume of ∼0.95 cm^3^ g^–1^ at *P*/*P*_0_ = 1.

These differences in porosity between the sets are likely to arise from the different modes of defect-compensation. As Iso-OH induces a higher degree of defects but does not compensate for the induced defects with ∼0.1–0.3 modulators per missing linker, the compensation by OH/H_2_O pair plays two important roles. First, the volume of the pairs is smaller than the volume of the Iso-OH modulator, thus leading to higher free space, and second, the weight of the pairs is significantly smaller than the weight of the modulator, and since the N_2_ adsorption/desorption is given as a function of the MOF’s mass, this results also in a higher amount of adsorbed gas per gram. Adding these two features together results in a remarkable overall increase in porosity.

The changes are also apparent in the pore size distribution, represented in Figure 3e and f. Our previous studies show that missing cluster defects, consequence of the spatial distribution of missing linkers, results in changes in the pore size distribution with the formation of a bigger pore at ∼11–12 Å and a smaller pore at ∼8 Å, while missing linkers (without missing clusters) only result in the formation of a smaller pore.^46^ Both samples display changes in the pore size distribution (Figure 3e,f), displaying new pores at ∼11 and 8 Å that are in great agreement with the formation of missing cluster defects.^46^ However, the MUV-10-Iso-F(83) and (91) samples also display a bigger pore at ∼17 Å that suggests the presence of nanoregions that are rich in missing cluster defects.^48^

Looking closer at the micro- and mesopore contribution from both modulated sets reveals that the changes in mesoporosity are more noticeable upon Iso-F modulation, which displays a significant increase in external surface area (Table 1), beneficial for photocatalytic applications.^12^ Analyzing the contribution of the micropore and external surface area to the overall porosity (Table 1, see section S.3.8) shows that the Iso-OH samples display an external surface of ∼6–14% with no relation with the modulator incorporation, having MUV-10-Iso-OH(91) an ∼8% of external surface area that is comparable to pristine MUV-10 (6% of external surface area). In contrast, the MUV-10-Iso-F samples display a palatine increase in external surface area in consonance with Iso-F incorporation reaching up to a ∼23% external surface area upon MUV-10-Iso-F(83) and (91) that corresponds to ∼230 m^2^ g^–1^.

Overall, although Iso-OH modulator induces a higher number of defects that are not fully compensated by the modulator resulting in higher microporosity, the defects compensated by Iso-F modulator are in close proximity (possibly nanoregions) resulting in a higher increase of the pore size and induced mesoporosity.^48^

In summary, these results highlight the complexity of the coordination modulation equilibria upon the introduction of competing modulators during synthesis. Modulators have a direct impact on the deprotonation, complexation, nucleation, and crystallization equilibria, but the inter-relation of these phenomena during the self-assembly processes is still not understood.^26^ While the acidity of the modulator disrupts the deprotonation of the linker, the ratio between modulator and linker deprotonated species will be higher for a more acidic modulator,^31^ thus leading to its preferred incorporation^33^ that could increase the metal complexation and nucleation kinetics. In this regard, since defects have been proposed to be the kinetic product for UiO MOFs,^49,50^ a less acidic modulator could result in a higher number of defects due to the slower self-assembly kinetics. It is worth noting that studies are often focused on the introduction of the desired functionality, whereas exhaustive characterization of the resultant properties is often dismissed.

With these interesting changes in mind, we aim to study the differences in photocatalytic activity and to relate them with the materials’ properties, which is also often not thoroughly performed in the available literature.

## Photocatalytic H_2_ Evolution

**Figure 4:**
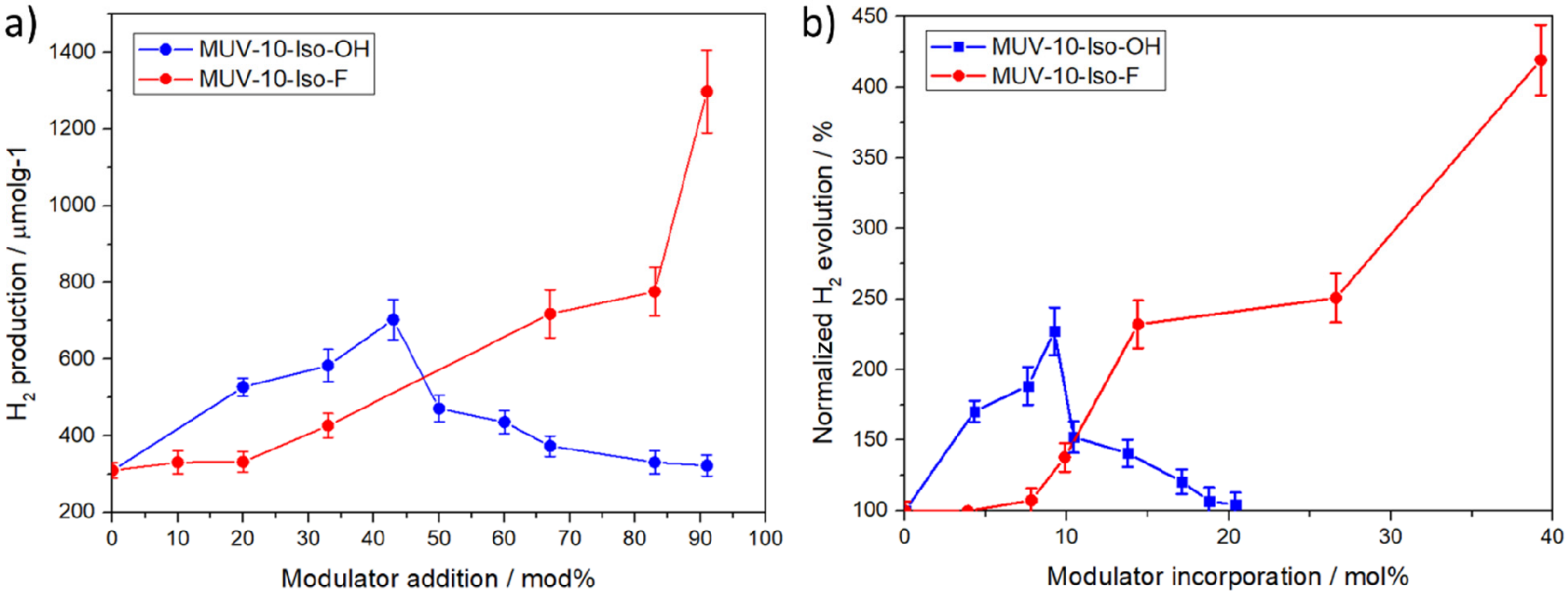
(a) H_2_ evolution after 24 h irradiation as a function of modulator addition and (b) normalized H_2_ evolution after 24 h irradiation as a function of modulator incorporation. Normalization was performed toward the 2 μm pristine MUV-10 sample as 100%.

The influence of Iso-OH and Iso-F modulators incorporation in the photocatalytic activity was tested for the H_2_ evolution reaction (see section S.1 for experimental and measurement conditions). For the sake of comparison, the photocatalytic activity of pristine MUV-10 was also included in the study with particle sizes of 2 μm and 200 nm, given that the introduction of modulators results in a decrease in particle size. The results show that very similar photocatalytic H_2_ evolution was obtained for 200 nm and 2 μm pristine MUV-10 (Tables S13–14), meaning that particle size does not play an important role in the photocatalytic H_2_ evolution with these photocatalysts under these conditions and that the photocatalytic reaction takes place mainly inside of the MOF, while the external surface area has a minor role in the photocatalytic activity. Normalized H_2_ evolution from the 1 mg/mL dispersions in H_2_O:MeOH (considering the H_2_ formed with MUV-10 as photocatalyst as 100%) of the different MUV-Iso-X samples after 24 h irradiation is shown in Figure 4, while full H_2_ production profiles (0.5, 1.5, 3, 6, and 24 h in μmol g^–1^) are shown in section S4.1 of the Supporting Information (Tables S15–S28 and Figures S52–S55).

Figure 4a shows that the addition of small amounts of the Iso-OH modulator (20–43%, corresponding to 0.25–0.75 equiv) has a beneficial effect on the photocatalytic H_2_ evolution, with a 1.70-fold increase (527 ± 24 μmolg^–1^) upon 20% addition in comparison to the unfunctionalized MUV-10 samples (309 ± 20 μmolg^–1^). However, after an optimum Iso-OH modulator addition of 43% (ca. 9 mol % of Iso-OH incorporated generating ca. 37 mol % of missing linker defects) with a 2.27-fold increase (703 ± 52 μmol g^–1^) compared to pristine MUV-10, further incorporation of Iso-OH in MUV-10 results in a detrimental effect, with photocatalytic activity gradually decreasing for further Iso-OH incorporation reaching a slightly higher production than the pristine MOFs for MUV-10-Iso-OH(91). Diverging from Iso-OH effect, the incorporation of Iso-F modulator has demonstrated a beneficial effect in the photocatalytic H_2_ evolution (Figure 4a), in which photocatalytic H_2_ production increases continuously with the modulator incorporation, obtaining a H_2_ production of 1298 ± 107 μmol g^–1^, corresponding to a 4.2-fold increase compared to the pristine material after 24 h irradiation for MUV10-Iso-F(91).

Figure 4b, which analyses the normalized H_2_ production as a function of the modulator incorporation in mol %, shows that there is an optimal incorporation degree for Iso-OH modulation, lower or higher incorporation than 9 mol % decreasing the photocatalytic H_2_ generation, that becomes similar to that unfunctionalized MUV-10 for high Iso-OH incorporation levels. In contrast, low Iso-F incorporation degrees result in similar H_2_ production to the pristine material, whereas incorporation above ∼14.4 mol % results in higher H_2_ production than the analogue Iso-OH modulated samples, increasing gradually with further incorporation. Control experiments in the absence of light, by covering the quartz photoreactor with aluminum foil while the light was on, were also carried out using these optimum photocatalysts, but undetectable H_2_ amounts were found after 24 h, indicating that light is responsible for H_2_ generation. These differences between Iso-OH and Iso-F modulated samples cannot be ascribed to changes in particle size or modulator content, given that the samples with the smallest particle sizes and higher modulator content in the Iso-OH series have worse performance.

Interestingly, MUV-10-Iso-OH(43) and MUV-10-Iso-F(91) have a similar molar percent of missing linkers, suggesting that ∼40 mol % of missing linker is the optimal amount of defects, in consonance with our previous studies reaching similar defect degrees with unfunctionalized isophthalic acid into the same framework.^45^ The volcano-type trend has been previously reported for other defected frameworks as photocatalyst,^37,39^ indicating that excessive amounts of defects can be detrimental, as defects can turn into recombination centers of electron–hole pairs as well as affect their chemical stability.

## Origin of Different Photocatalytic Activity

It has been previously observed that carboxylate-containing MOFs undergo decarboxylation upon UV light irradiation, evolving CO_2_,^51^ but there is still scarce literature covering this effect or the *in situ* stability of the materials during photocatalytic reactions. In fact, the photogeneration of defects through oxidation of formate and acetate modulators to generate holes that increase the photocatalytic activity of UiO-66 (Zr) has recently been reported,^18^ although the photodegradation during the catalytic process was not thoroughly assessed. This brought us to analyze the CO_2_ released (due to photodecarboxylation) during the photocatalytic H_2_ production reactions. On the basis of our compositional models obtained by combining TGA and ^1^HNMR,^47^ we have calculated the % of CO_2_ released in relation to the maximum amount of CO_2_ from all the carboxylate moieties present in the samples (see section S.4.2 for calculation of structural CO_2_ release, Tables S29–S33 and Figures S56 and S57). First, a difference in CO_2_ release was observed upon the 2 μm and 200 nm pristine samples, suggesting that decarboxylation initially occurs at the sample’s surfaces, with a ∼8% structural CO_2_ evolution of the biggest and ∼19% for the smallest pristine sample after 24 h of reaction, despite them having a similar H_2_ evolution (Table S29). The CO_2_ release was in tune with the photocatalytic activity of the modulated samples, with MUV-10-Iso-F(X) displaying higher H_2_ evolution when the structural CO_2_ release was lower than for MUV-10-Iso-OH(X) (Figures S56 and S57). In fact, while the CO_2_ production of the Iso-F samples is mostly maintained across the series with a ∼21–25% of structural CO_2_ released, it increases gradually among the Iso-OH series, from 14% CO_2_ for MUV-10-Iso-OH(20) to 20% CO_2_ for MUV-10-Iso-OH(43) and 38% CO_2_ for MUV-10-Iso-OH(91). Our CO_2_ evolving results could be explained by the different defect-compensation modes, which previously showed reduced stability for the MUV-10-Iso-OH samples due to their high amount of defect being compensated by monocoordinated OH^–^/H_2_O pairs.

To further prove that the differences in photocatalytic performance are related to the sample’s differences in photochemical stability coming from the differences in defect compensation and not due to differences in the photogenerated charges or excited states lifetime, we performed photocurrent and transient absorption spectroscopy (TAS) measurements.

**Figure 5:**
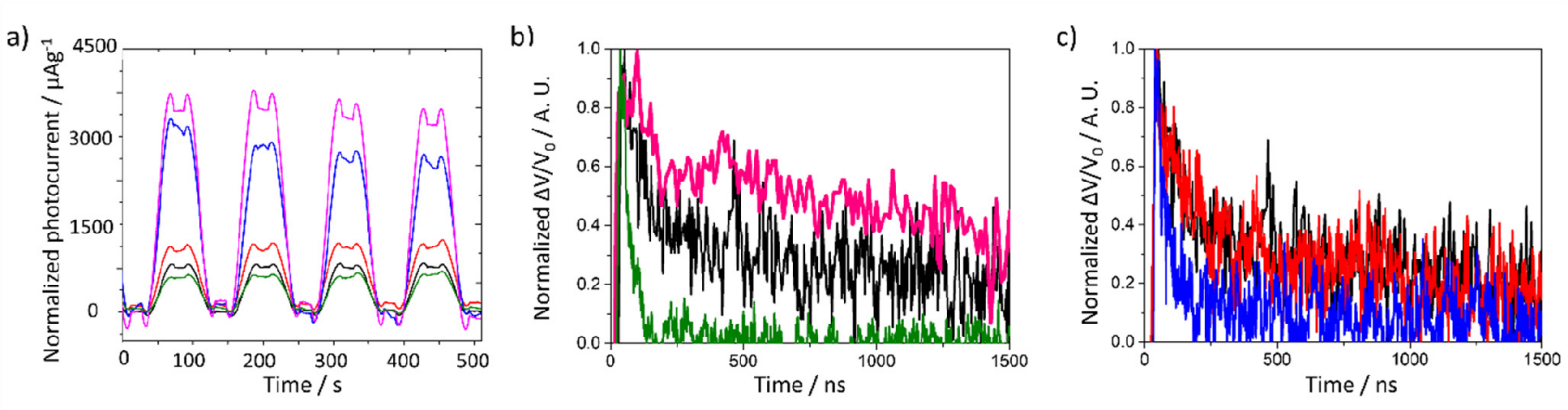
(a) Photocurrent experiments using MUV10 (black), MUV10-ISO-OH(33) (pink), MUV10-ISO-OH(91) (green), MUV10-ISO-F(33) (red), and MUV10-ISO-F(91) (blue) supported on FTO substrates alternating light (120 mW/cm^2^) and dark cycles of 60 s under 0.5 V applied bias vs Ag/AgCl_sat._ electrode. Electrolyte KCl 0.1 M, counter electrode Pt wire. (b) Normalized transient absorption kinetics of MUV-10 (black), MUV-10-Iso-OH(33) (pink), and MUV-10-Iso-OH(91) (green). (c) Normalized transient absorption kinetics of MUV-10 (black), MUV-10-Iso-F(33) (red) and MUV-10-Iso-OH(91) (blue). Laser excitation at 355 nm, monitored at 640 nm.

Photocurrent experiments (Figure 5a) on MUV-10, MUV-10-Iso-OH(33), MUV-10-Iso-OH(91), MUV-10-Iso-F(33), and MUV-10-Iso-F(91) were carried out in 4 consecutive cycles of 60 s with the light switched on and off, and the results are presented in section S.4.3, together with the photoelectrode fabrication procedure. The results show that all MOFs display an instant increase in current density when exposed to light, indicating photoinduced charge generation. The photocurrent of MUV-10-Iso-OH(33) was higher (3450 μA g^–1^) than that measured for MUV-10-Iso-OH(91) (759 μA g^–1^), in good agreement with the obtained H_2_ evolution from MUV-10-Iso-OH(33) (583 μmol g^–1^) and MUV-10-Iso-OH(91) (322 μmol g^–1^). Similarly, the photocurrent obtained from MUV-10-Iso-F(33) was lower (1030 μA g^–1^) than that obtained from MUV-10-Iso-F(91) (2830 μA g^–1^), in good agreement with the obtained H_2_ evolution for these samples, 426 μmol g^–1^ and 1298 μmol g^–1^, for MUV-10-Iso-F(33) and MUV-10-Iso-F(91), respectively. However, it is worth commenting that the highest photocurrent was obtained from MUV-10-Iso-OH(33), despite the highest H_2_ evolution values obtained with the MUV-10-Iso-F(91) sample, for which the photocurrent is 1.2-fold lower than that of MUV-10-Iso-OH(33), thus indicating that photoinduced charge generation is not the main reason for the differences in photocatalytic activity between these two samples.

Diffuse reflectance UV–vis spectra measurements allowed for the optical band gap estimation of the different samples (see section S.4.4). The results show that the band gap of MUV-10-Iso-OH samples decreases upon Iso-OH incorporation from ∼3.3 eV for pristine MUV-10 to 2.3 eV for MUV-10-Iso-OH(33) (Figures S58 and S59), resulting in yellow samples. However, further Iso-OH addition did not produce significant band gap reduction, being 2.1 eV for MUV-10-Iso-OH(91). Surprisingly, MUV-10 did not suffer significant band gap changes upon Iso-F modulator addition (Figure S60). Therefore, the photocatalytic activity increase at low Iso-OH incorporation could be ascribed to a decrease in band gap due to a light-harvesting increase in the visible region, which is also in good agreement with the photocurrent experiments’ data. However, the decrease in the photocatalytic activity at high Iso-OH incorporation, together with the higher H_2_ production at high Iso-F incorporation, must be attributed to different causes.

To rule out the influence of excited states species and their deactivation kinetics, TAS measurements have been carried out in isoabsorbing dispersions of MUV-10, MUV-10-Iso-OH(33), MUV-10-Iso-OH(91), MUV-10-Iso-F(33), and MUV-10-Iso-F(91) (Figure 5b,c) in acetonitrile under N_2_ atmosphere upon 355 nm laser excitation (Figures S61–S65). MUV-10 transient absorption spectrum exhibits two main absorption bands with similar intensity and centered at 460 and 640 nm (Figure S61). Quenching experiments in O_2_ saturated atmosphere and upon MeOH addition (15% Vol.) were carried out to rule out the origin of these excited states. However, neither O_2_, a well-known electron acceptor, nor MeOH, as hole scavenger, promoted noticeable quenching of the excited states or significant lifetime reduction. The excited states deactivation kinetics followed very similar dynamics independently of the monitored wavelength (ca. 460 or 640 nm), indicating that both bands belong to the same species. In both cases, the deactivation kinetics followed a bimodal behavior, which can be fitted to a biexponential function (eq 1 in section S.5.1). From the fitting of the experimental data, we obtained a lifetime of 52 ns for the faster component, monitored at 640 nm, while the second decay lifetime is 0.87 μs. MUV-10-Iso-OH(33) and MUV-10 presented similar transient absorption spectra, although the relative intensity of the 640 nm band in MUV-10-Iso-OH(33) is larger than that of 460 nm (Figure S62). Surprisingly, MUV-10-OH(33) excited states absorption was partially quenched, and the kinetics lifetime decreased upon MeOH addition, while exposure to O_2_ atmosphere did not promote noticeable changes, indicating that the photoinduced excited states can be partially attributed to holes, with a minor or negligible contribution of electrons. Moreover, the deactivation kinetics in MUV-10-Iso-OH(33) is very different from MUV-10, with MUV-10-Iso-OH(33) deactivation kinetics presenting a single component that can be fitted to a single exponential function (eq 2 in section S.5.1). In this case, long-lived excited states, assigned mainly to holes at 640 nm, can be measured, and data fitting to eq 2 result in a lifetime of 1.28 μs for the 640 nm band and 1.56 μs for the band at ∼460 nm.

MUV-10-Iso-OH(91) shows a similar transient absorption spectrum to that of MUV–OH(33) (Figure S63). However, the photoinduced excited states cannot be assigned only to holes, as in the case of MUV-10-Iso(33), since both O_2_ and MeOH quench similarly the transient signal, indicating a similar contribution of both species. Interestingly, the deactivation kinetics is much faster than MUV-10-Iso-OH(33), with a lifetime of 38 ns at 630 nm and 32 ns at 460 nm. This faster excited states lifetime can be attributed to the faster recombination rate in MUV-10-Iso-OH(91) than in MUV-10-Iso-OH(33), which would explain the higher photocurrent and photocatalytic activity of MUV-10-Iso-OH(33), when compared with MUV-10-Iso-OH(91), despite the band gap in these samples being comparable.

MUV-10-Iso-F(33) transient absorption spectrum is very similar to that of MUV-10-Iso-OH(33) (Figure S64). However, quenching experiments with O_2_ and MeOH revealed that in this case this band can be attributed to the presence of electrons since both the signal intensity and lifetime is only reduced in the presence of O_2_. Moreover, the deactivation kinetics in MUV-10-Iso-F(33) follows a bimodal behavior exhibiting similar kinetics to MUV-10. In this case, fitting of the experimental data to a biexponential function (eq 1) resulted in lifetimes of 0.106 and 0.704 μs at 640 nm and 0.119 and 0.660 μs at 460 nm.

MUV-10-F(91) TAS also show bands at ∼460 and 640 nm, whereas their separation is significantly more resolved than in pristine MUV-10 and other functionalized samples (Figure S65). Additionally, a new band at ∼700 nm is apparent. Similarly to MUV-10-Iso-OH(91), MUV-10-Iso-F(91) kinetics lifetime shows a fast (37 ns), single-component behavior, and the excited state’s species cannot be clearly assigned to either electron or holes. This result falls in contradiction with the previous observations in the photocurrent and photocatalytic H_2_ evolution measurements. Therefore, we must hypothesize that the improved photocatalytic activity in MUV-10-Iso-F(91) cannot be ascribed to an improvement in light-harvesting or recombination suppression but other factors related to the better stability during the reaction. In fact, the initial H_2_ production rates, acquired during the first reaction hour, are very similar in both series (see section S.4.1). As the main differences in H_2_ production appear at long reaction times, this should be related to the better photostability in the MUV10-iso-F(X) samples since the other studied factors should be reflected in the photocatalytic activity from the very beginning.

## Conclusions

We report that introducing modulators through coordination modulation results in drastic changes in compositional and textural properties that highlight the complex equilibria occurring during the self-assembly process and the importance of deep characterization beyond the incorporation of functionalized modulators.

Here we show that changing a functional group in the modulator results in different defect compensation that has a drastic effect on the materials’ optoelectronics, porosity, and stability. The Iso-OH modulator promotes bandgap reduction and longer excited states lifetime. However, the Iso-F modulator is more efficient than the Iso-OH modulator in compensating for the induced defectivity of the samples, thus leading to both higher thermal and chemical stabilities than the analogue Iso-OH modulated samples.

All-in-all, the photocatalytic data show that the incorporation of the Iso-OH moiety in the MUV-10 structure plays initially a beneficial effect as a consequence of the band gap reduction that low incorporation of Iso-OH produces. However, after a certain Iso-OH modulator amount (43% addition, ca. 9.2 mol % incorporation), further incorporation causes detrimental effects in the photocatalytic activity of MUV-10-Iso-OH(X), while the photocatalytic activity of the Iso-F modulated samples is improved continuously with Iso-F incorporation as a consequence of the stability effects. Since the H_2_ production rate is similar for all the samples at early stages of the reaction, while experiments with pristine samples of different sizes showed no significant differences in the H_2_ production, the differences at long reaction times are mostly due to the differences in photostability coming from the different defect-compensation and hydrophilic/hydrophobic nature of the modulators. Importantly, the most active samples, MUV-10-Iso-OH(43) and MUV-10-Iso-F(91) present a similar degree of defects (ca. 40 mol %), indicating that further defect induction is detrimental for photocatalytic H_2_ evolution due to induced samples instability.

## Supporting Information Available

Synthetic conditions, full characterization of MOFs (PXRD, NMR, SEM, EDX, FT-IT, TGA, and N2 adsorption and desorption isotherms), photocatalytic experiments (experimental conditions, tabulated values of conversion, normalized conversion, relation with synthetic variables, photocatalytic decarboxylation, photocurrent experiments, band gap, and transient absorption spectroscopy) (PDF)
