# Supplementary material for: Tuning the Photocatalytic Activity of Ti-Based Metal–Organic Frameworks through Modulator Defect-Engineered Functionalization
Source: ACS Appl Mater Interfaces. 2022 Apr 28;14(18):21007–17. doi: 10.1021/acsami.2c02668 (PMC9100481; doi:10.1021/acsami.2c02668)
Supplement: Supplementary file 1 — am2c02668_si_001.pdf [file am2c02668_si_001.pdf]

Supporting Information

Tuning the photocatalytic activity of Ti-based MOFs through modulator defect-engineered functionalization

Isabel Abánades Lázaro[a]†*, Horatiu Szalad[b]†, Pablo Valiente[a], Josep Albero[b], Hermenegildo García[b] and Carlos Martí-Gastaldo[a]

[a]Instituto de Ciencia Molecular (ICMol), Universitat de València, Catedrático José Beltrán Martínez no 2, 46980 Paterna, València, Spain.

[b]Instituto Universitario de Tecnología Química CSIC-UPV, UniversitatPolitècnica de València, Av. De los Naranjos s/n, 46022 València, Spain.

† I.A.L and H.S contributed equally regarding experimental work.

*Email:isabel.abanades@uv.es

Table of contents

S.1. General Experimental Remarks 2

S.2. Materials and Synthesis 3

S.3. Characterisation of MUV-10-Iso-OH 4

S.3.1. Powder X-Ray Diffraction (PXRD) 4

S.3.2. Metal ratio calculated by Energy Dispersed X-Ray 5

S.3.3. Fourier transformed Infra-Red (FT-IR) 6

S.3.3.a. Fourier transformed Infra-Red (FT-IR) of Iso-OH modulated samples 6

S.3.3.b. Fourier transformed Infra-Red (FT-IR) of Iso-F modulated samples 8

S.3.4. Proton Nuclear Magnetic Resonance (^{1}HNMR) 9

S.3.4.a. Proton Nuclear Magnetic Resonance (^{1}HNMR) of Iso-OH modulated samples 9

S.3.4.b. Proton Nuclear Magnetic Resonance (^{1}HNMR) of Iso-F modulated samples 11

S.3.5. Scanning Electron Microscopy (SEM) 13

S.3.5.a. Scanning Electron Microscopy (SEM) of Iso-OH modulated samples 13

S.3.5.b. Scanning Electron Microscopy (SEM) of Iso-F modulated samples 16

S.3.5.c. Energy-dispersive X-Ray analysis (EDX) mapping. 18

S.3.6. Thermogravimetric analysis (TGA) 20

S.3.6.a. Thermogravimetric analysis (TGA) of Iso-OH modulated samples 21

S.3.6.b. Thermogravimetric analysis (TGA) of Iso-F modulated samples 25

S.3.7. Water and Methanol Stability 29

S.3.8. Nitrogen Adsorption and desorption measurements 31

S.3.8.a. Nitrogen Adsorption and desorption measurements of Iso-OH modulated samples 31

S.3.8.b. Nitrogen Adsorption and desorption measurements of Iso-F modulated samples 35

S.4. Photocatalysis 37

S.4.1.a. Photocatalysis of MUV-10-Iso-OH 38

S.4.1.b. Photocatalysis of MUV-10-Iso-F 42

S.4.2. Photocalytic decarboxylation 45

S.4.3. Photocurrent experiments 47

S.4.4. Band Gap 48

S.5. Transient absorption spectroscopy 50

S.6. References 53

S.1. General Experimental Remarks

Powder X-Ray Diffraction (PXRD): PXRD patterns were collected in a PANalytical X'Pert PRO diffractometer using copper radiation (Cu Kα = 1.5418 Å) with an X'Celerator detector, operating at 40 mA and 45 kV. Profiles were collected in the 3° < 2θ < 40° range with a step size of 0.017°. (University of Valencia)

Thermogravimetric Analysis (TGA): were carried out with a Mettler Toledo TGA/SDTA 851 apparatus between 25 and 800 °C under ambient conditions (10 $°C·min^{-1}$ scan rate and an air flow of 9 mL·min−1). (University of Valencia)

Nuclear Magnetic Resonance Spectroscopy (NMR): NMR spectra were recorded on either a Bruker AVIII 300 MHz spectrometer and referenced to residual solvent peaks. (University of Valencia)

Gas Uptake: N_2 adsorption isotherms were carried out at 77 K on a with a Micromeritics 3Flex gas sorption analyser. Samples were degassed under vacuum at 120 °C for 24 h in a Multisorb station prior to analysis. BET surface areas, micropore surface areas and external surface areas were calculated from the isotherms using the MicroActive operating software. The pore size distributions were calculated using DFT cylindrical pore oxide surface model within the MicroActive software,(University of Valencia)

Scanning Electron Microscopy (SEM) and single point energy-dispersive X-Ray analysis (EDX): particle morphologies, dimensions and mapping were studied with a Hitachi S-4800 scanning electron microscope at an accelerating voltage of 20 kV, over metalized samples with a mixture of gold and palladium during 90 seconds. (University of Valencia)

Fourier Transform Infrared Spectroscopy: IR spectra of solids were collected using a Shimadzu Fourier Transform Infrared Spectrometer, FTIR-8400S, fitted with a Diamond ATR unit. (University of Valencia)

UV-Vis diffuse reflectance spectroscopy (DRS) was performed on a Jasco V-670 spectrophotometer using an integrated Labsphere in the range 200-800 nm. (University of Valencia)

Photocatalytic experiments: H_2 evolution upon UV-Vis irradiation with a 300 W Xe lamp was tested by dispersing 20 mg of the different samples in Milliq-H_2O:MeOH mixtures (4:1, v:v) at 1 mg/mL in a quartz photoreactor, purging exhaustively with Ar before irradiation. Analysis of the evolved gases has been carried out through a micro-GC with two columns (Molsieve 5A and PorePlot Q) and TC detector that allowed us to monitor and quantify H_2, O_2, N_2, CO_2, among other gases. (Universidad politecnica de Valencia)

S.2. Materials and Synthesis

All reagents unless otherwise stated were obtained from commercial sources and were used without further purification.

General remarks

For all modulated syntheses a mixture of solvents (2.2 mL of AcOH per 9.6 mL of DMF) was prepared in function of the number of reactions to perform (11.8 mL per reaction). This pre-made solvent mixture was used to separately dissolve the different synthetic components as further explained during this section.

In all syntheses the jars were placed in an oven at room temperature and heated to 120℃ with 2℃/min ramp. The temperature was maintained during 24 hours and cooled down to room temperature with 0.4℃/min ramp. The resultant powder was collected by centrifugation (5000 rpm, 5 min) and washed with DMF (X2) and MeOH (x3) through dispersion-centrifugation cycles. The samples were dried under vacuum overnight and further activated by sohxlet with MeOH during approximately 24 hours. The samples were further dried under vacuum for 24 hours prior to characterization.

Procedure MUV-10-Iso-OH%: In 25 mL pyrex jars, $CaCl_2$ (1 equivalent) was dissolved in 2 mL of solvent mixture. In a separate vial 1.5 equivalents of btc compared to Ti and Ca were dissolved in 9.8 mL of solvent mixture together with the modulator (number of equivalents compared to btc as desired depending on the synthetic conditions). Both solutions were mixed in in 25 mL pyrex jars followed by slow Ti(IV) isopropoxide addition (1 equivalent) and gentle stirring.

Table S1: Tabulated synthetic conditions.

Sample's labelling	$CaCl_2$	$Ti(O^iPr)_4$	btc	Modulator	Mod Vs btc
	**mmol**	**mmol**	**mmol**	**mmol**	**Equivalents**
**MUV-10 (H)**	0.6 mmol	0.6 mmol	0.9 mmol	0	0
**20%**	0.6 mmol	0.6 mmol	0.9 mmol	0.225	0.25
**33%**	0.6 mmol	0.6 mmol	0.9 mmol	0.45	0.5
**43%**	0.6 mmol	0.6 mmol	0.9 mmol	0.675	0.75
**50%**	0.6 mmol	0.6 mmol	0.9 mmol	0.9	1
**60%**	0.6 mmol	0.6 mmol	0.9 mmol	1.35	1.5
**67%**	0.6 mmol	0.6 mmol	0.9 mmol	1.8	2
**83%**	0.6 mmol	0.6 mmol	0.9 mmol	4.5	5
**91%**	0.6 mmol	0.6 mmol	0.9 mmol	9	10

S.3. Characterisation of MUV-10-Iso-OH

S.3.1. Powder X-Ray Diffraction (PXRD)

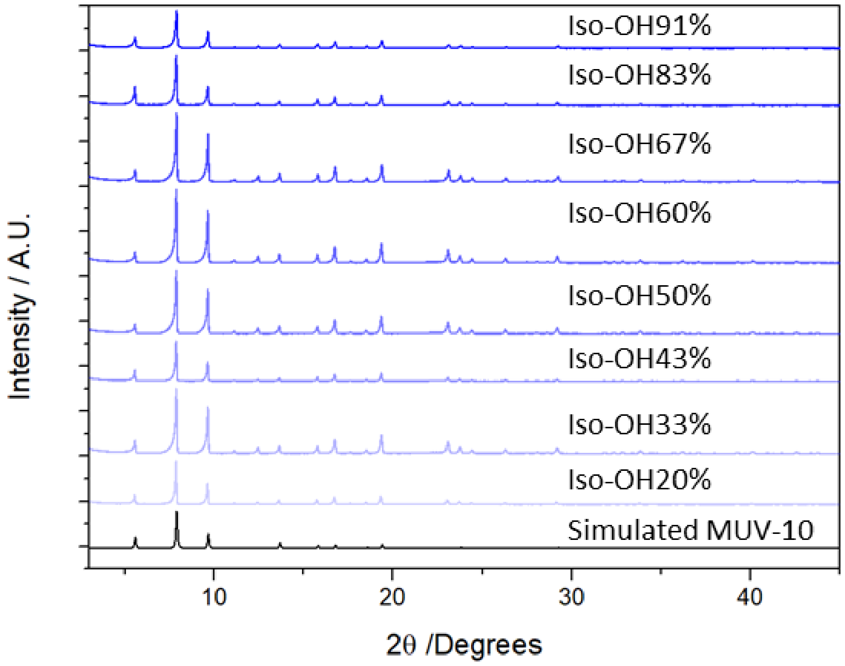

Figure S1: PXRD patterns of MUV-10 modulated with 5-hydroxy isophthalic acid compared to simulated MUV-10, showing a moderate increase in crystallinity (intensity) when the quantity of isophthalic acid added to the synthetic mixture increases.

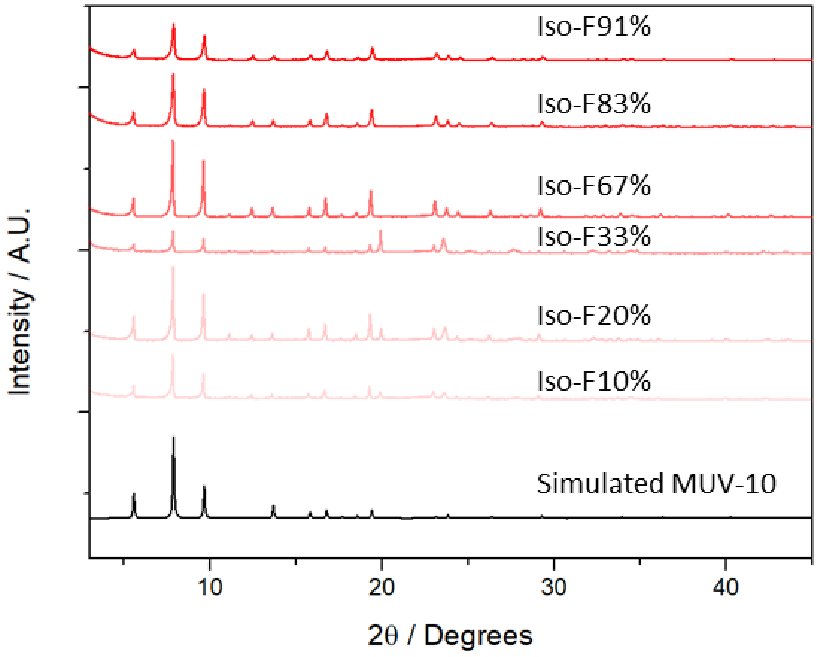

Figure S2: PXRD patterns of MUV-10 modulated with 5-Fluoro isophthalic acid compared to simulated MUV-10, showing a moderate increase in crystallinity (intensity) when the quantity of isophthalic acid added to the synthetic mixture increases.

S.3.2. Metal ratio calculated by Energy Dispersed X-Ray

Table S2: Tabulated metal Ca molar percent versus total amount of metal (Ti and Ca) of MUV-10-Iso-OH.

Sample	0%	20%	33%	43%	50%	60%	67%	83%	91%
**Ca molar % EDX**	45.6	44.5	43.9	43.9	44.5	44.0	41.8	44.0	45.0
**SD**	0.3	0.3	0.2	0.4	0.2	0.1	0.5	0.1	0.4

Table S3: Tabulated metal Ca molar percent versus total amount of metal (Ti and Ca) of MUV-10-Iso-F.

sample	0%	10%	20%	33%	67%	83%	91%
**Ca molar % EDX**	45.6	45.9	42.5	42.9	42.2	44.3	45.7
**SD**	0.3	0.4	0.2	0.4	0.5	0.1	0.5

S.3.3. Fourier transformed Infra-Red (FT-IR)

S.3.3.a. Fourier transformed Infra-Red (FT-IR) of Iso-OH modulated samples

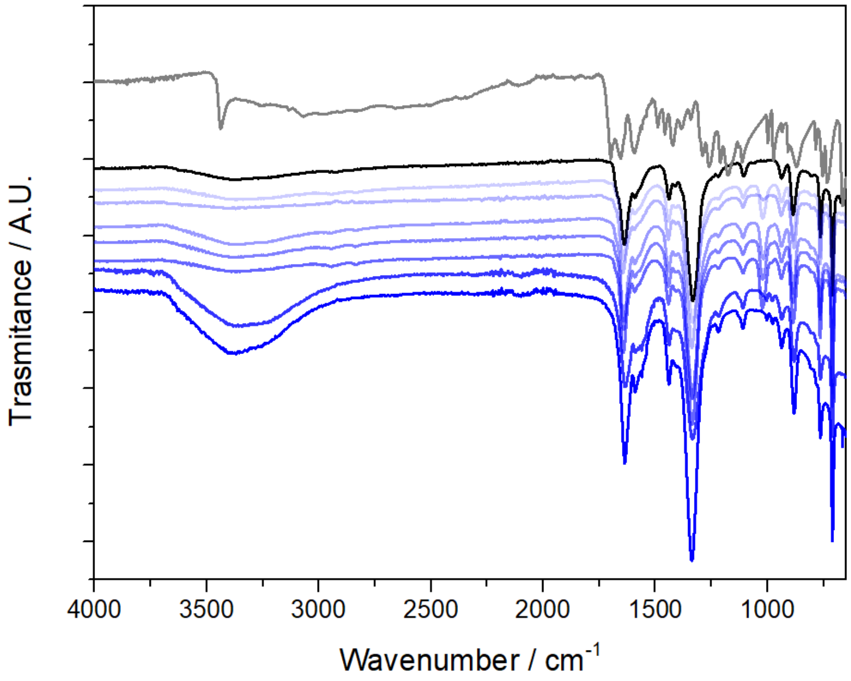

Figure S3: Raw FT-IR profiles of MUV-10 synthesised with increased Iso-OH concentration compared to pristine MUV-10. The OH broad band at ca. 3250 cm^{-1} is in great agreement with the high incorporation determined by ^{1}HNMR.

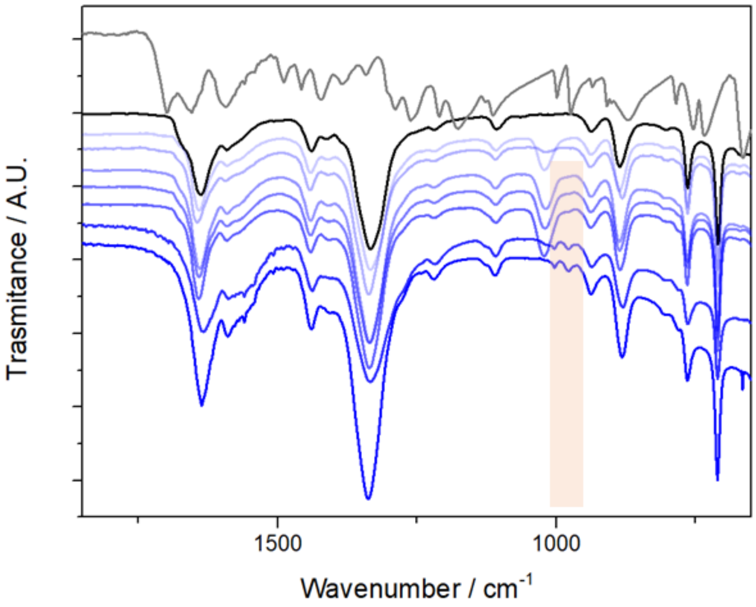

Figure S4: Amplification of raw FT-IR profiles of MUV-10 synthesised with increased Iso-OH concentration compared to pristine MUV-10. Changes in the metal vibration bands at *ca.* 650 cm^{-1}, together with the broadening of the carboxylate signals, indicates defectivity. The absence of free carboxylate bands confirms the modulator attachment to the metal clusters. The O-H *ip* (ca. 1400 cm^{-1}) band is masked with MUV-10 carboxylate vibration bands while the C-O *st* can be observed at *ca.* 1000 cm^{-1}.

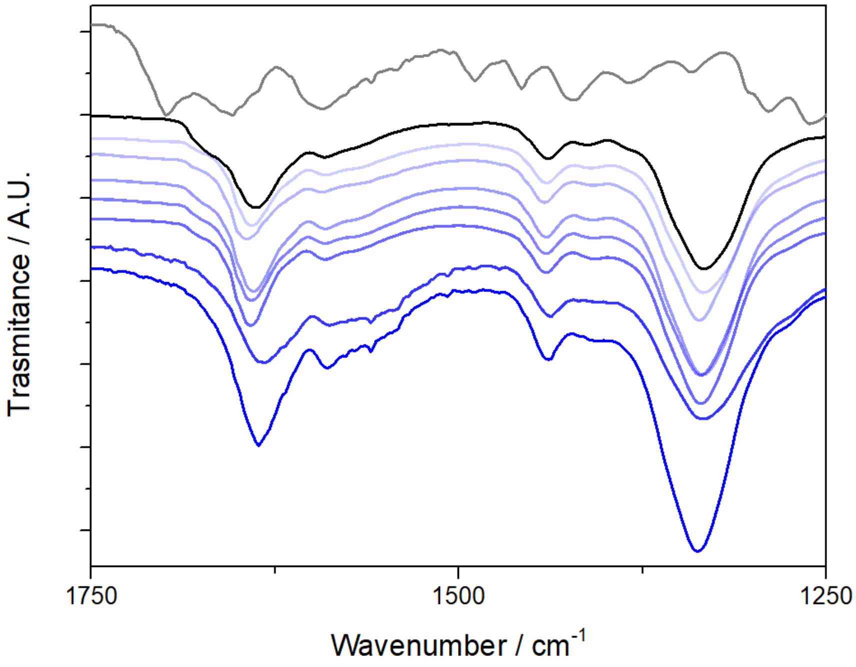

Figure S5: Amplification of raw FT-IR profiles of MUV-10 synthesised with increased Iso-OH concentration compared to pristine MUV-10. Shifting in MUV-10 characteristic vibration bands can be appreciated consequence of the modulator's attachment.

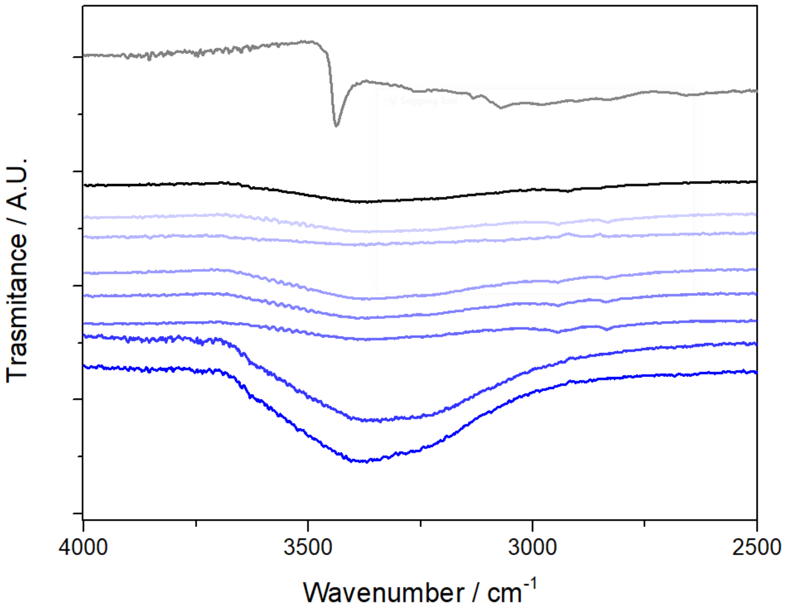

Figure S6: Amplification of raw FT-IR profiles of MUV-10 synthesised with increased Iso-OH concentration compared to pristine MUV-10. The O-H *st* (*ca.* 3250 cm^{-1}) broad band is in great agreement with the high incorporation determined by ^{1}HNMR.

S.3.3.b. Fourier transformed Infra-Red (FT-IR) of Iso-F modulated samples

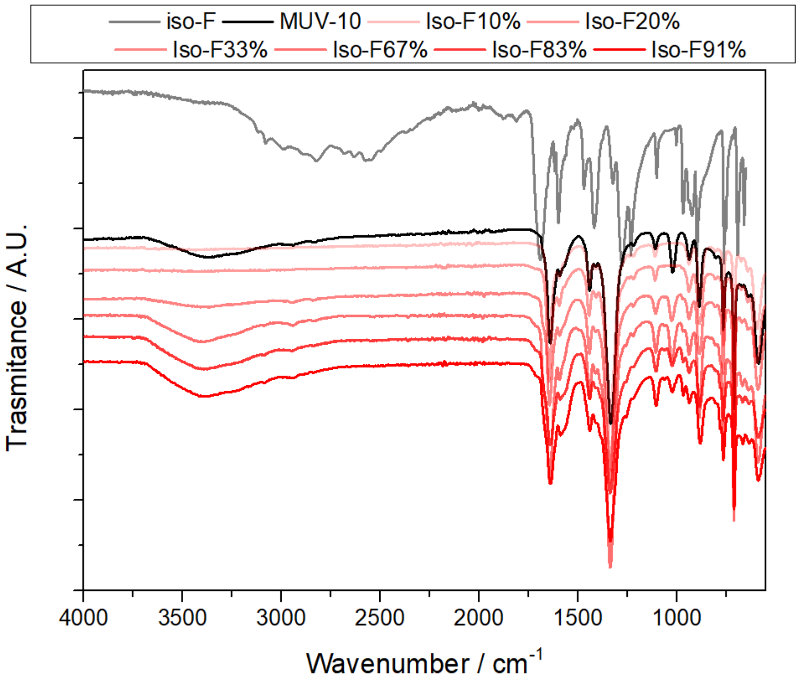

Figure S7: Raw FT-IR profiles of MUV-10 synthesised with increased Iso-F concentration compared to pristine MUV-10.

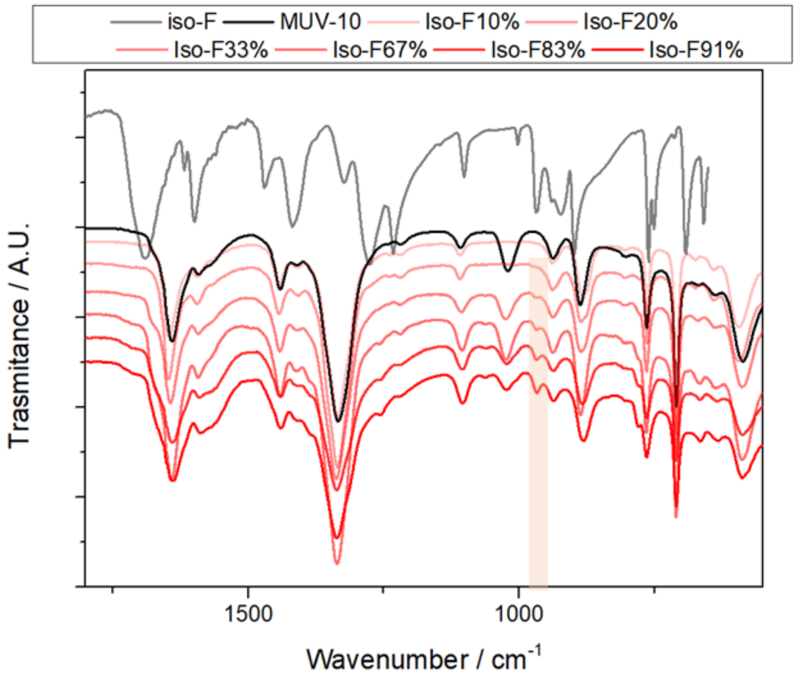

Figure S8: Amplification of raw FT-IR profiles of MUV-10 synthesised with increased Iso-F concentration compared to pristine MUV-10. Changes in the metal vibration bands at ca. 650 cm^{-1}, together with the broadening of the carboxylate signals, indicates defectivity. The absence of free carboxylate bands confirms the modulator attachment to the metal clusters. Iso-F shows characteristic vibration bands of the modulators such as C-F bending at *ca.* 967 cm^{-1}

S.3.4. Proton Nuclear Magnetic Resonance (^{1}HNMR)

Iso-OH was present in the ^{1}HNMR profiles alongside formic acid coming from the decomposition of DMF during synthesis. Incorporation of the modulator and formic acid is expressed as the **molar ratio** (R_{mod},) between modulator and btc, $\mathrm{Rmod} = \frac{\mathrm{Mod}}{\mathrm{btc}}$ and as the **molar percent** of modulator (mol%) compared to btc, mol%= $\frac{\mathrm{Mod}}{\mathrm{Mod+btc}} * 100$, while the **total modulator percent** (total mod%) is calculated taking into account modulator, formic acid and btc, $\mathrm{total\ mod\%} = \frac{\mathrm{Mod+FA}}{\mathrm{Mod+FA+btc}} * 100$

S.3.4.a. Proton Nuclear Magnetic Resonance (^{1}HNMR) of Iso-OH modulated samples

Table S4: Tabulated data extracted from acid digested ^{1}HNMR of MUV-10-Iso-OH, showing Iso-OH and total modulator content increasing with the addition of modulator, whereas fa incorporation seems to be constant.

Sample	RMOD	%MOD	Rrfa	%fa	%tot
**20%**	0.045	4.286	0.017	1.673	5.820
**33%**	0.082	7.595	0.101	9.181	15.490
**43%**	0.102	9.231	0.011	1.106	10.143
**50%**	0.116	10.420	0.012	1.150	11.344
**60%**	0.160	13.761	0.011	1.105	14.584
**67%**	0.206	17.094	0.019	1.822	18.350
**83%**	0.231	18.785	0.014	1.369	19.690
**91%**	0.256	20.408	0.015	1.515	21.371

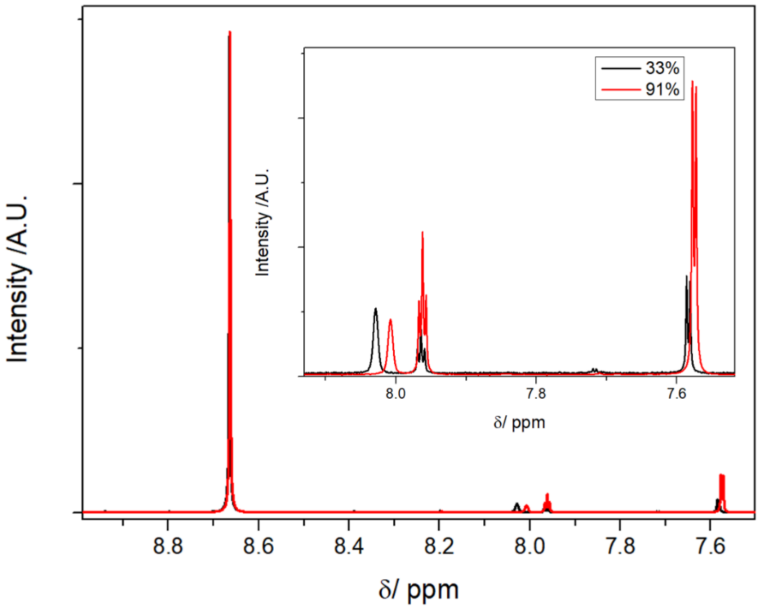

Figure S9: Representation of partial ^{1}HNMR profile of iso-OH 33% and iso-OH91%, showing increased presence in comparison to btc. Modulator incorporation is calculated from the integral at *ca.* 7.96 ppm (1H).

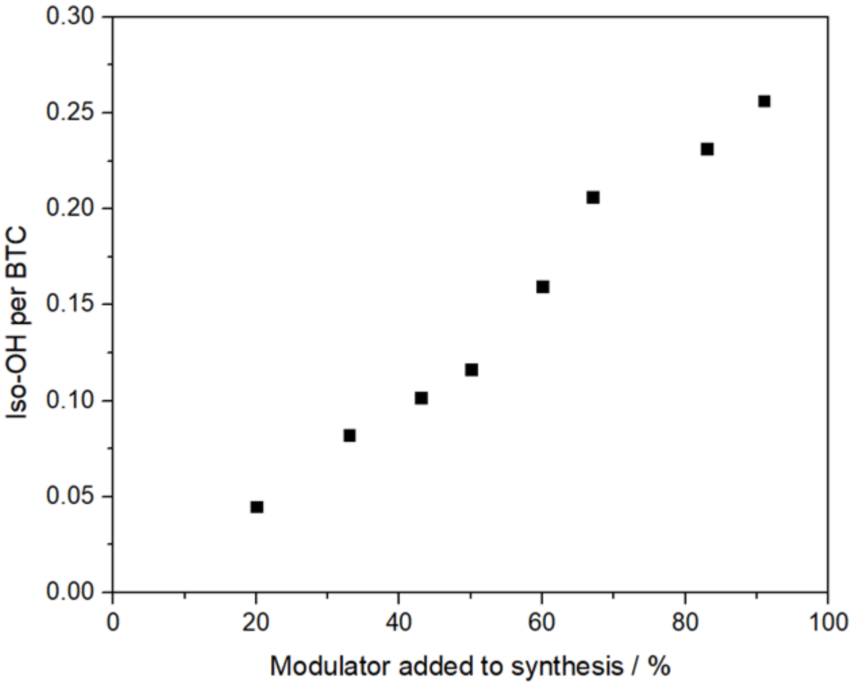

Figure S10: Iso-OH incorporation in molar ration as a function of the modulator percent added to the synthesis in comparison to btc.

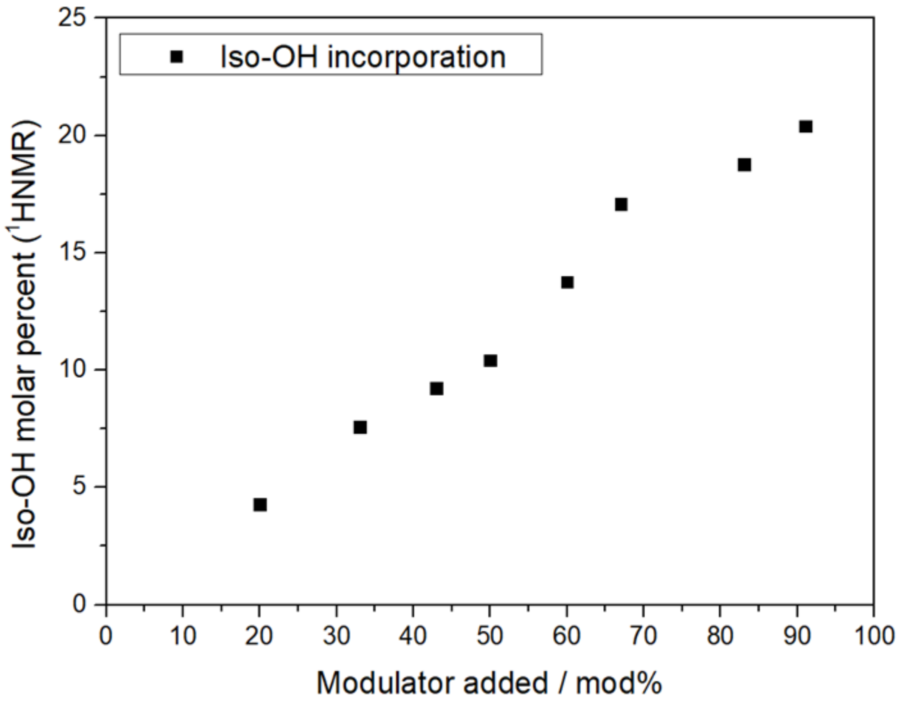

Figure S11: modulator incorporation in molar percent as a function of the modulator percent added to the synthesis in comparison to btc.

S.3.4.b. Proton Nuclear Magnetic Resonance (^{1}HNMR) of Iso-F modulated samples

Table S6: Tabulated data extracted from acid digested ^{1}HNMR of MUV-10-Iso-F, showing Iso-F and total modulator content increasing with the addition of modulator, whereas fa incorporation seems to be constant.

Sample	RMOD	%MOD	Rrfa	%fa	%tot
**10%**	0.040	3.880	0.028	2.748	5.775
**20%**	0.085	7.806	0.021	2.073	11.943
**33%**	0.110	9.873	0.116	10.403	26.814
**67%**	0.168	14.388	0.151	13.139	24.204
**83%**	0.363	26.619	0.116	10.401	32.379
**91%**	0.647	39.267	0.000	0.000	39.267

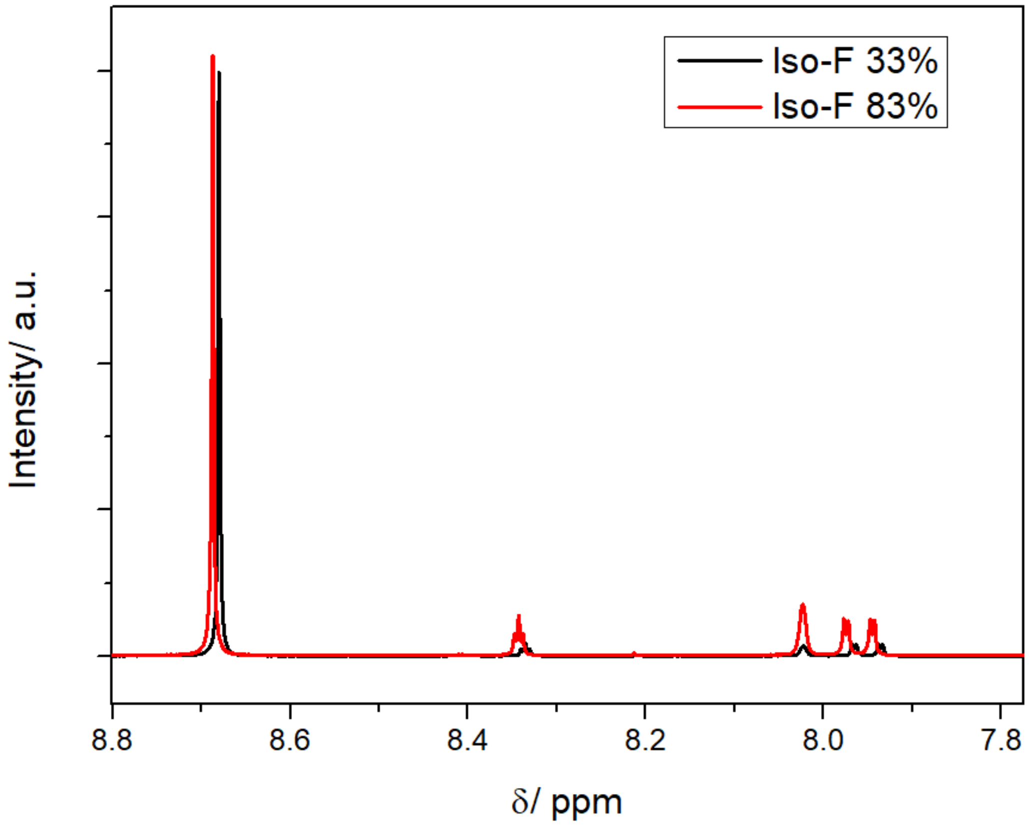

Figure S12: Representation of partial ^{1}HNMR profile of iso-F 20% and iso-F83%, showing increased presence in comparison to btc. Modulator incorporation is calculated from the integral at *ca.* 8.33 ppm (1H).

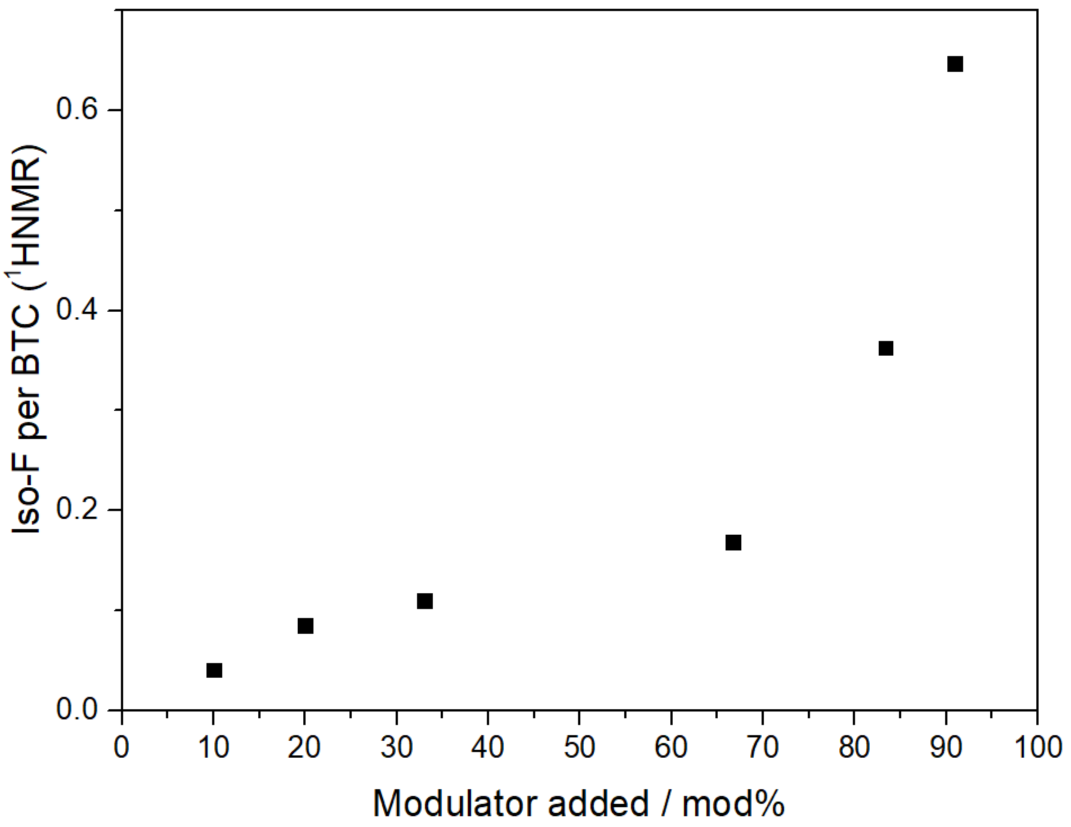

Figure S13: Iso-F modulator incorporation in molar ration as a function of the modulator percent added to the synthesis in comparison to btc.

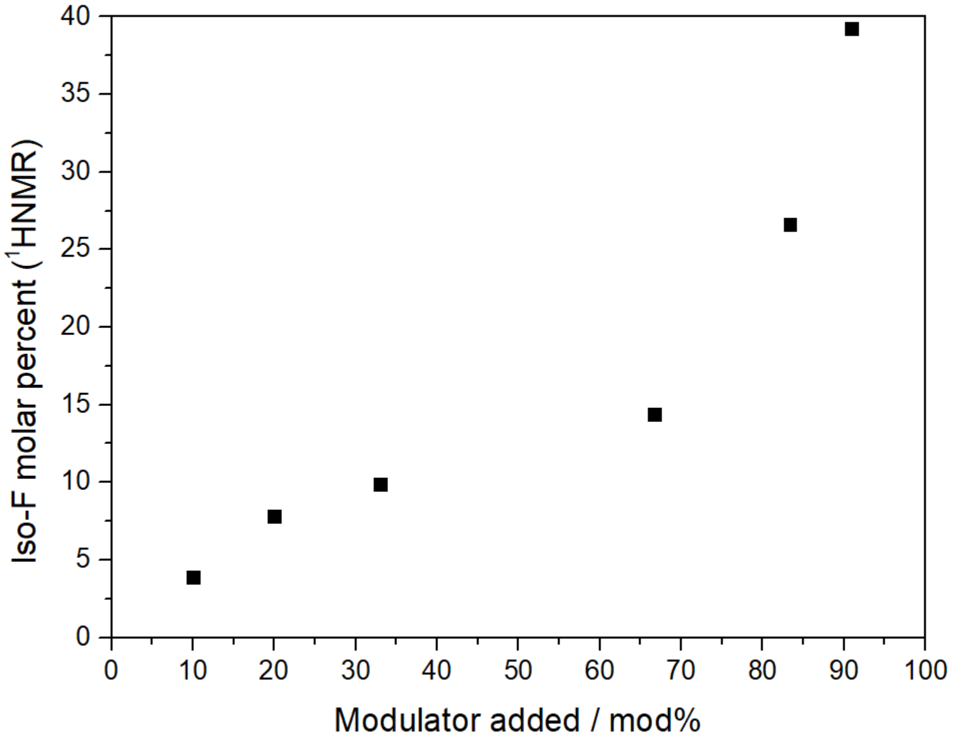

Figure S14: Iso-F modulator incorporation in molar percent as a function of the modulator percent added to the synthesis in comparison to btc.

S.3.5. Scanning Electron Microscopy (SEM)

S.3.5.a. Scanning Electron Microscopy (SEM) of Iso-OH modulated samples

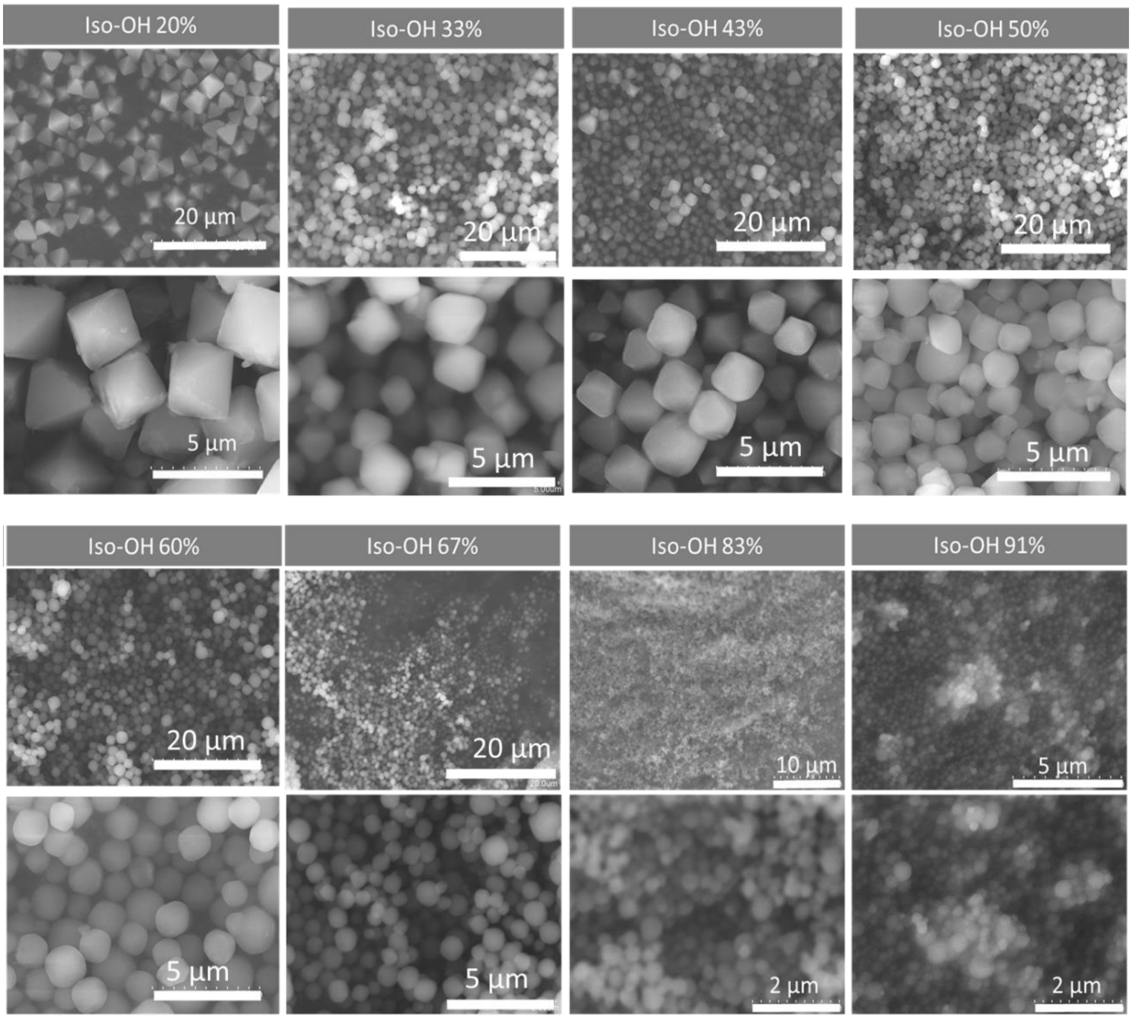

Figure S15: SEM images of MUV-10-Iso-OH, showing a change in morphology compared to the pristine MOF and a decrease in particle size with increasing concentration of modulator, indicating a capping effect and/or faster metal-complexation, nucleation and crystallisation kinetics.

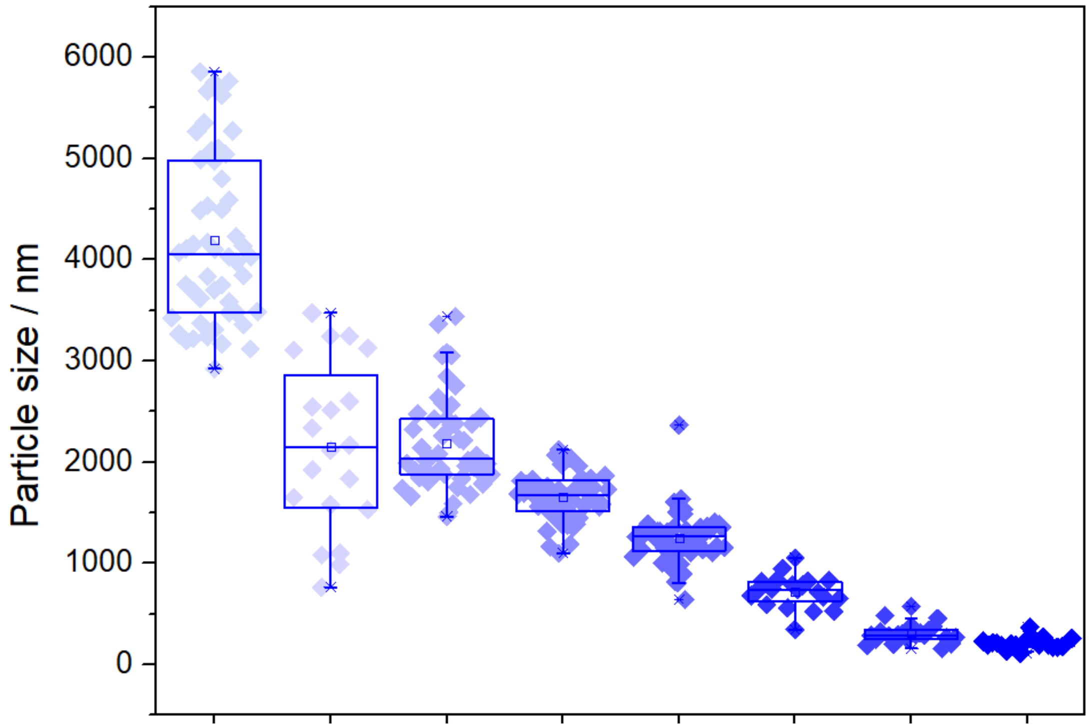

Figure S16: Box chart representation of MUV-10-Iso-OH particle sizes. Bin size of 200 nm. Average size and standard deviation, 25% and 75% quartiles.

Table S7: Tabulated particle sizes and standard deviations of MUV-10-Iso-OH.

**sample**	**20%**	**33%**	**43%**	**50%**	**60%**	**67%**	**83%**	**91%**
**Av size (nm)**	4111	1975	2144	1644	1227	700	297	204
**SD (nm)**	821	811	462	240.2	254	157	97	53

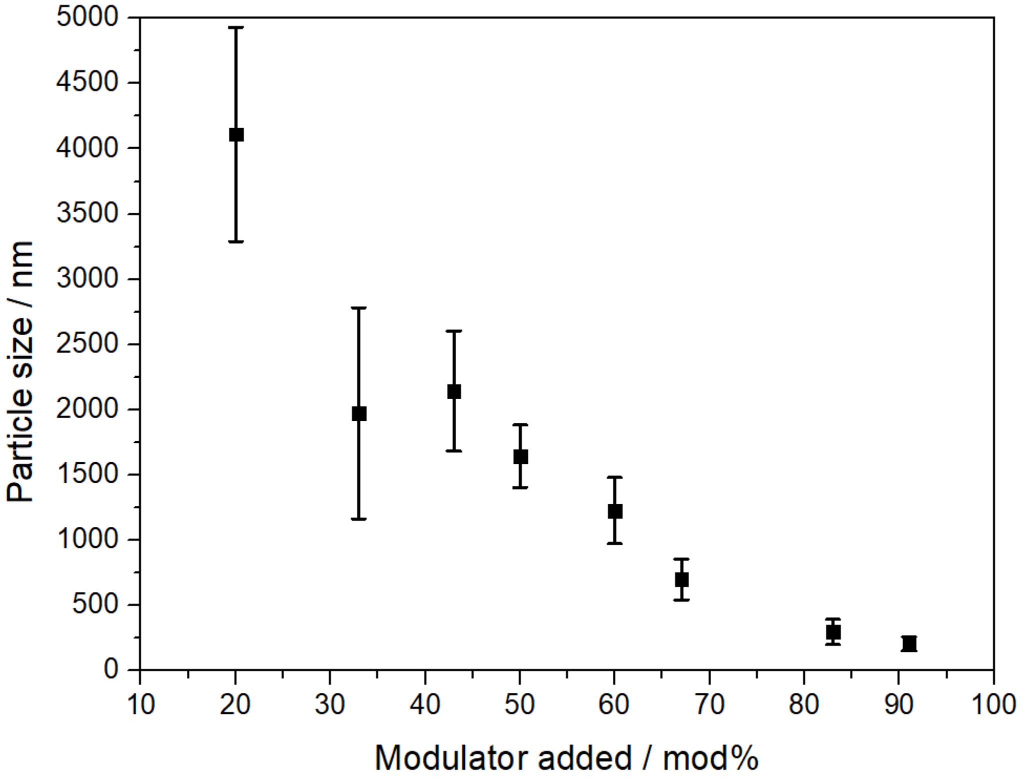

Figure S17: Representation of particle size of MUV-10-Iso-OH as a function of the modulator per cent added to the syntheses.

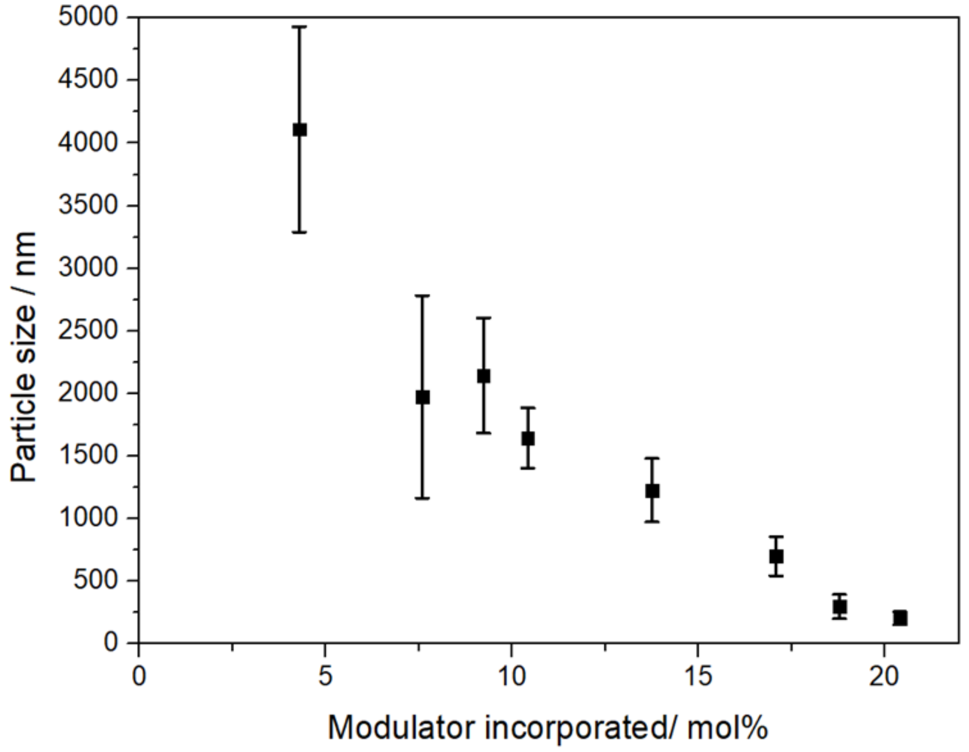

Figure S18: Representation of particle size of MUV-10 hierarchical crystals as a function of the modulator incorporated in molar percent.

S.3.5.b. Scanning Electron Microscopy (SEM) of Iso-F modulated samples

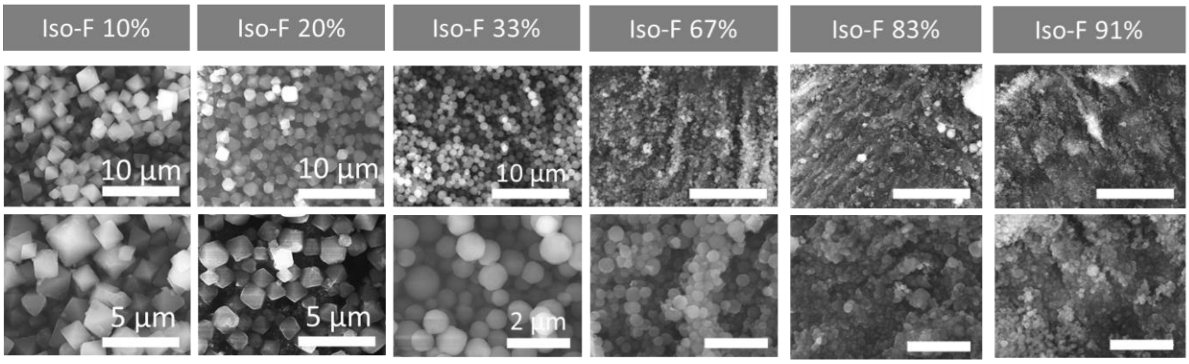

Figure S19: SEM images of MUV-10-Iso-F, showing a change in morphology compared to the pristine MOF and a decrease in particle size with increasing concentration of modulator, indicating a capping effect and/or faster metal-complexation, nucleation and crystallisation kinetics.

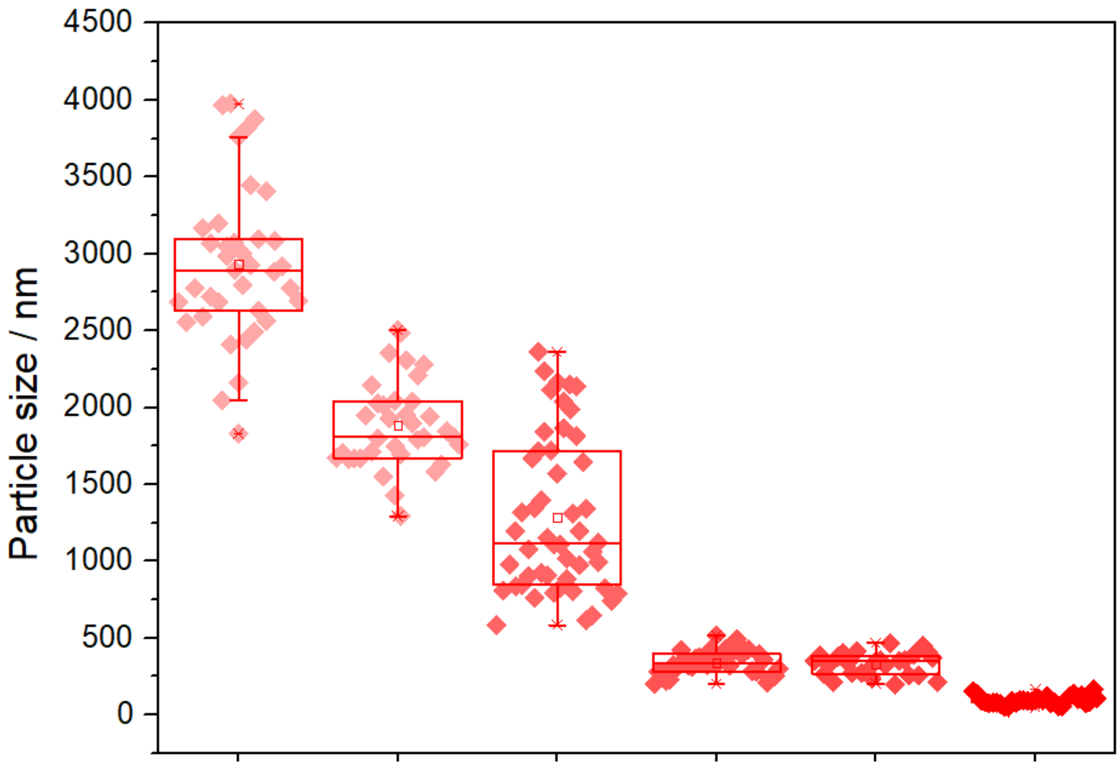

Figure S20: Box chart representation of MUV-10-Iso-F particle sizes. Bin size of 200 nm. Average size and standard deviation, 25% and 75% quartiles.

Table S8: Tabulated particle sizes and standard deviations of MUV-10-Iso-F.

Sample	10%	20%	33%	67%	83%	91%
Av size (nm)	2931	1882	1193	330	321	91
SD (nm)	502	280	501	76	72	27

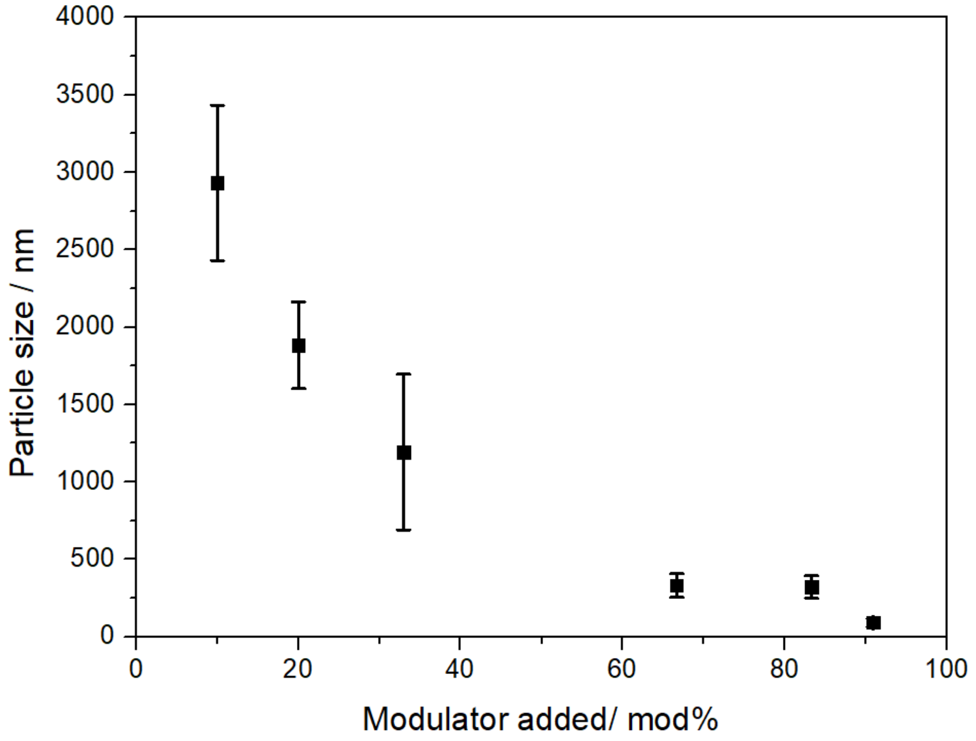

Figure S21: Representation of particle size of MUV-10-Iso-F as a function of the modulator per cent added to the syntheses.

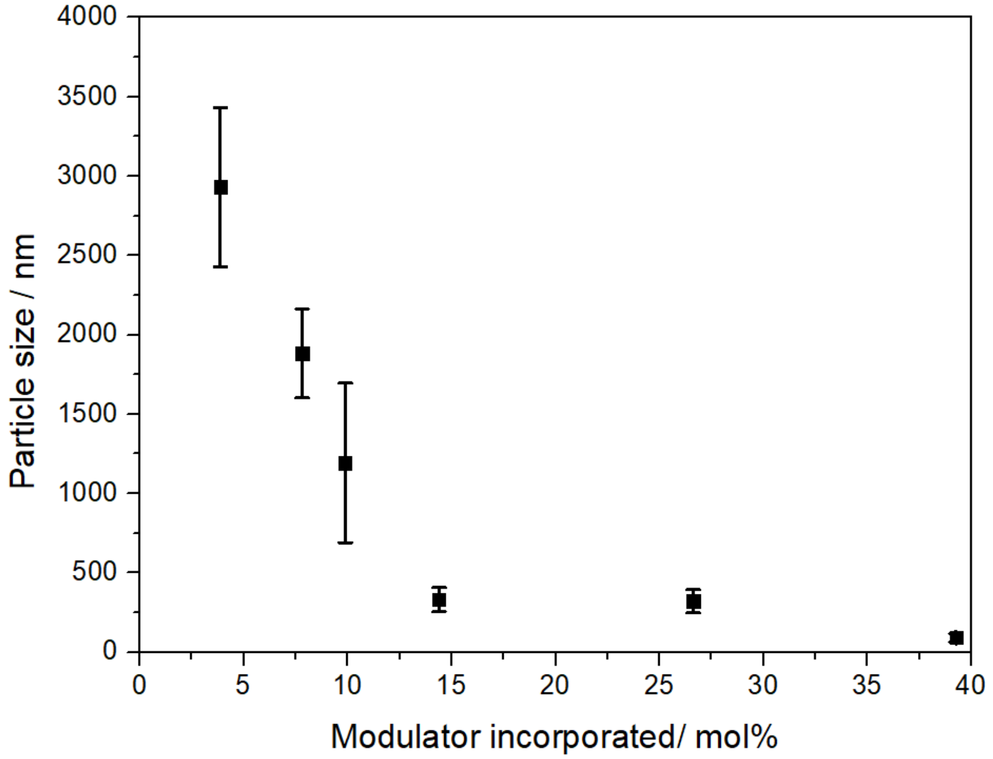

Figure S22: Representation of particle size of MUV-10-Iso-F as a function of the modulator incorporated in molar percent.

S.3.5.c. Energy-dispersive X-Ray analysis (EDX) mapping.

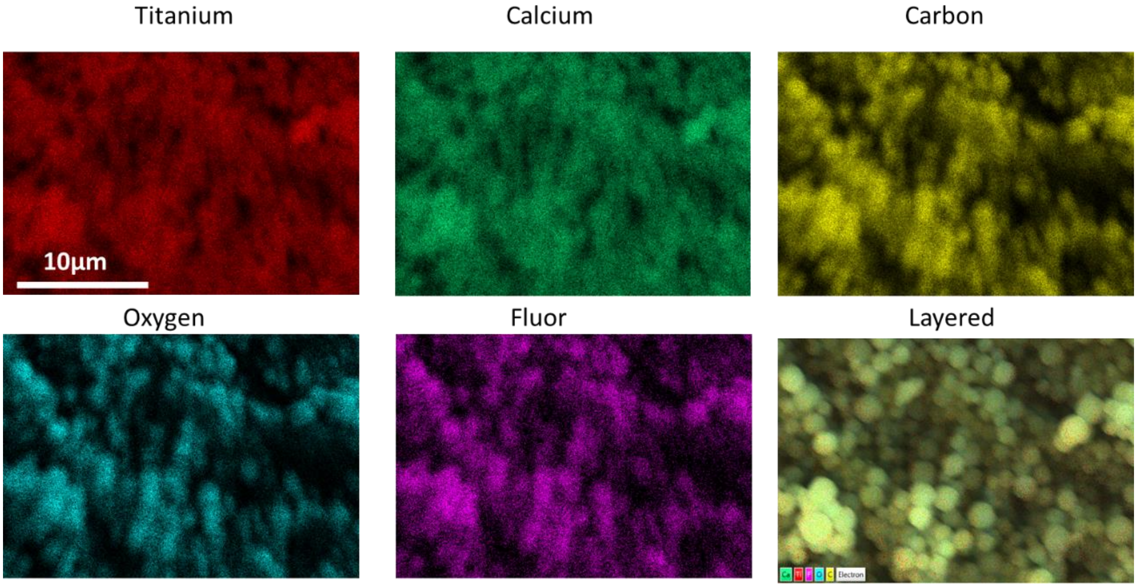

Figure S23: EDX mapping images of MUV-10-Iso-F63.

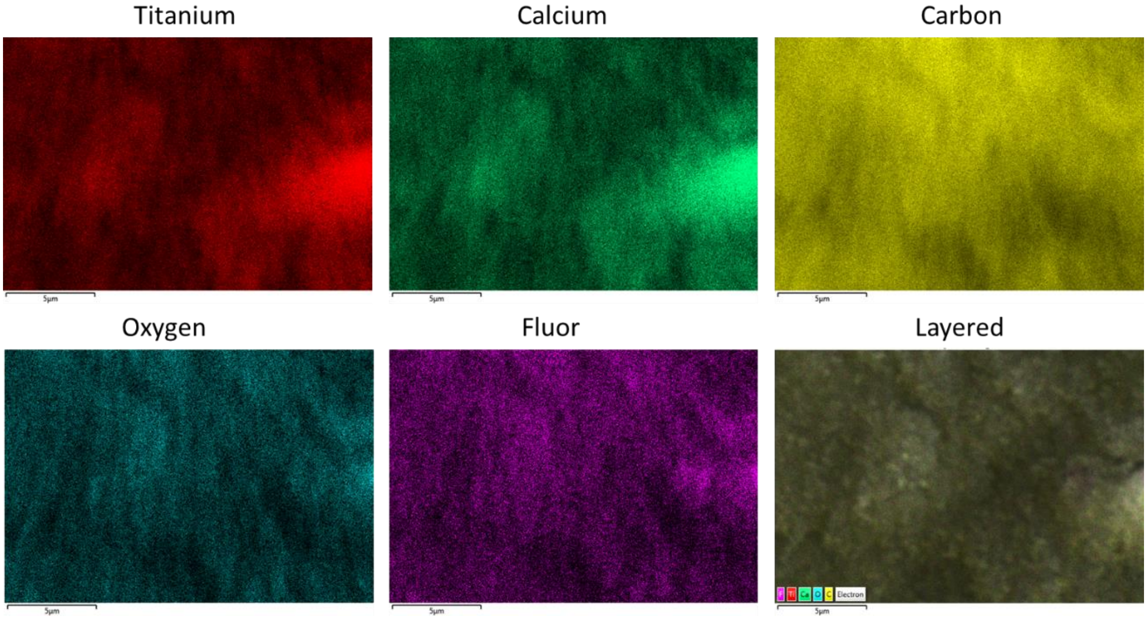

Figure S24: EDX mapping images of MUV-10-Iso-F91.

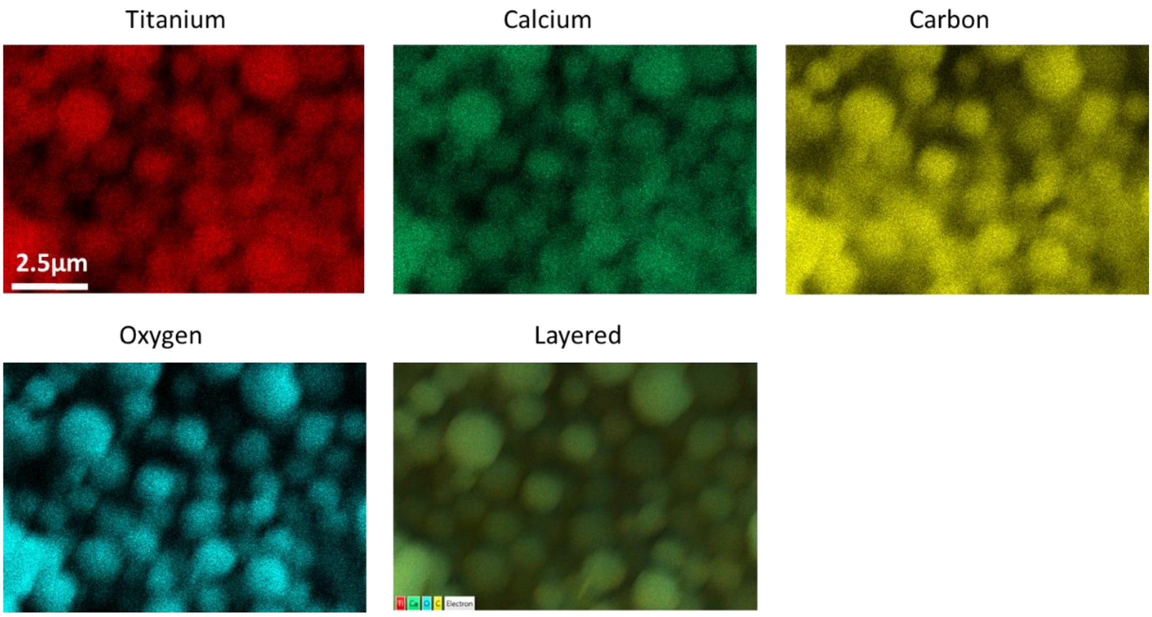

Figure S25: EDX mapping images of MUV-10-Iso-OH43.

S.3.6. Thermogravimetric analysis (TGA)

We have analysed the composition of MUV-10-Iso-X through combination of TGA with molar ratios determined by ^{1}HNMR, assuming that Iso-X is incorporated into MUV-10 structure **$TiCaO(H_2O)_W(BTC)_X(ISO)_y(FA)_z(OH)_D$**.[1] As Iso-X decomposes during the decomposition range of BTC, the experimental ratio between the molecular weigh of the DH MOF and its residue is expressed as follows:

$$\text{Experimental}\frac{\text{DH MOF}}{\text{Residue}}\text{Ratio}\left(\text{R}_{\text{expDH}}\right)=\frac{\text{M}_\text{w}\text{ [T400 °C]}}{\text{M}_\text{w}\text{[Residue]}}=\frac{\text{M}_\text{w}\text{ [TiCaO(BTC)}_\text{x}\text{(Iso}-\text{X)}_\text{y}\text{(O)}_{\frac{(4-3x-2y)}{2}}]}{\text{M}_\text{w}\text{ [TiCaO}_3]}$$

Since,

$$\text{y Iso}=\text{x BDC}*\left(\frac{\text{Iso}}{\text{BTC}}\right)\text{NMR ratio}$$

Then,

$$\text{R}_{\text{expDH}}=\frac{\text{M}_\text{w}\text{ [TiCaO(BTC)}_\text{x}\text{(Iso}-\text{X)}_{\text{xnmr}}\text{(O)}_{\frac{(4-3x-2xnmr)}{2}}]}{\text{M}_\text{w}\text{ [TiCaO}_3]}$$

Rearranging the equation

$$XBTC=\frac{\left(\text{R}_{\text{expDH}}*\ \text{M}_\text{w}\text{ [TiCaO}_3]\right)-\text{M}_\text{w}\text{ TiCaO}-2*\text{M}_\text{w}[O]}{\text{M}_\text{w}\ [BTC]+nmr*\text{M}_\text{w}[Iso-X]-\left(\frac{(3+2\text{nmr})}{2}\right)*\text{M}_\text{w}[O]}$$

Once X has been obtained,

$$\text{y Iso}=\text{x BDC}*\left(\frac{\text{Iso}-\text{X}}{\text{BTC}}\right)\text{NMR ratio}$$

$$\text{z Formate}=\text{number of BDC}*\left(\frac{\text{FA}}{\text{BTC}}\right)\text{NMR ratio}$$

The number of OH needed to compensate the charge can be calculated using the following equation:

$$4\text{Ti}+2\text{Ca}=2+3XBTC+XNMRFA+OH$$

Then $OH\ =4-3XBTC-XNMRFA$

Once the number of linkers, modulators and defect-compensating species has been determined, number of water molecules is calculated as:

$$\text{Experimental}\frac{\text{MOF}}{\text{Residue}}\text{Ratio}\left(\text{R}_{\text{exp}}\right)=\frac{\text{M}_\text{w}\text{ [T 100 °C]}}{\text{M}_\text{w}\text{[Residue]}}$$
$$=\frac{\text{M}_\text{w}\text{ [TiCaO(BTC)}_\text{x}(\text{H}_2O)_\text{z}\text{(Mod)}_\text{y}\text{(OH)}_\text{w}]}{\text{M}_\text{w}\text{ [TiCaO}_2]}$$

$$\text{Experimental}\frac{\text{MOF}}{\text{Residue}}\text{Ratio}\left(\text{R}_{\text{exp}}\right)=\frac{\text{M}_\text{w}\text{ [Calculated MOF]}+\text{x}*\text{M}_\text{w}\ [\text{H}_2O]}{\text{M}_\text{w}\text{ [TiCaO}_3]}$$

$$X\ (\text{H}_2O)=\frac{\left(\text{R}_{\text{exp}}*\ \text{M}_\text{w}\text{ [TiCaO}_3]\right)-\text{M}_\text{w}\text{ [Calculated MOF]}}{\text{M}_\text{w}\ [\text{H}_2O]}$$

S.3.6.a. Thermogravimetric analysis (TGA) of Iso-OH modulated samples

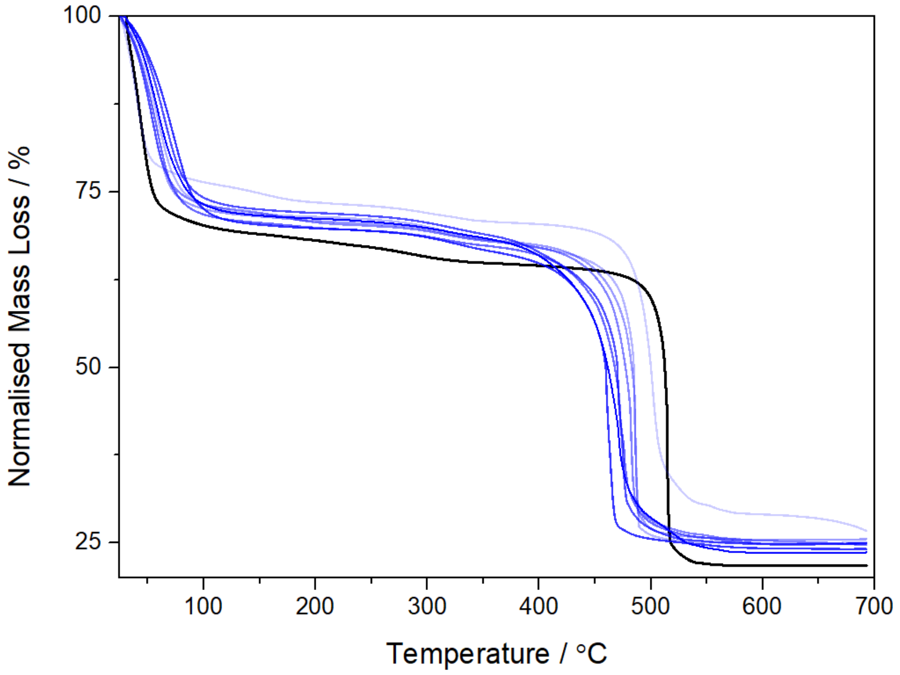

Figure S26: TGA profiles of MUV-10 synthesised with increased concentrations of Iso-OH, compared to pristine MUV-10, with the start of the decomposition profiles normalised to 100%. A slight increase in metal content can be appreciated.

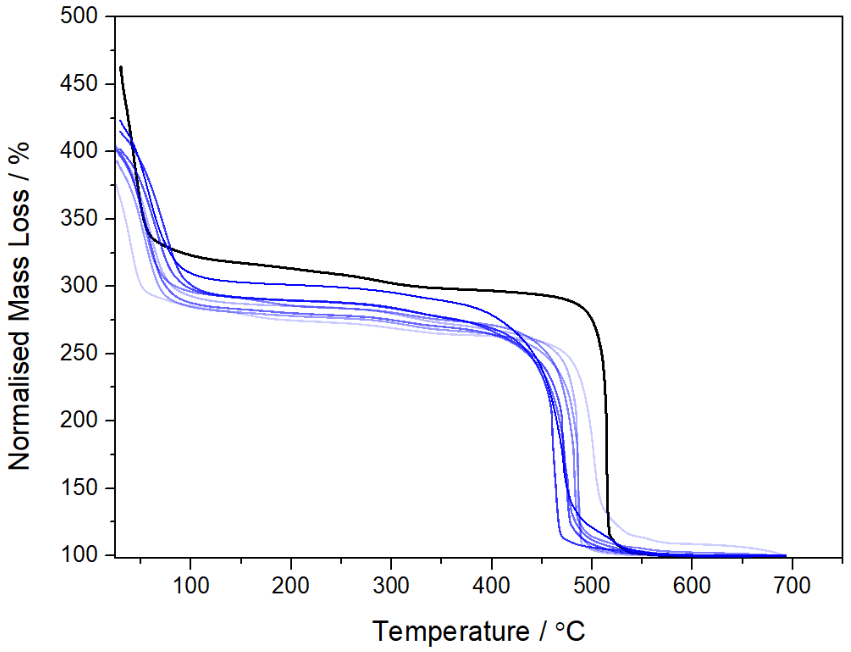

Figure S27: TGA profiles of MUV-10 synthesised with increased concentrations of Iso-OH, compared to pristine MUV-10, with the residues normalised to 100%. A decrease in the btc decomposition step is observed, alongside a decrease in thermal stability.

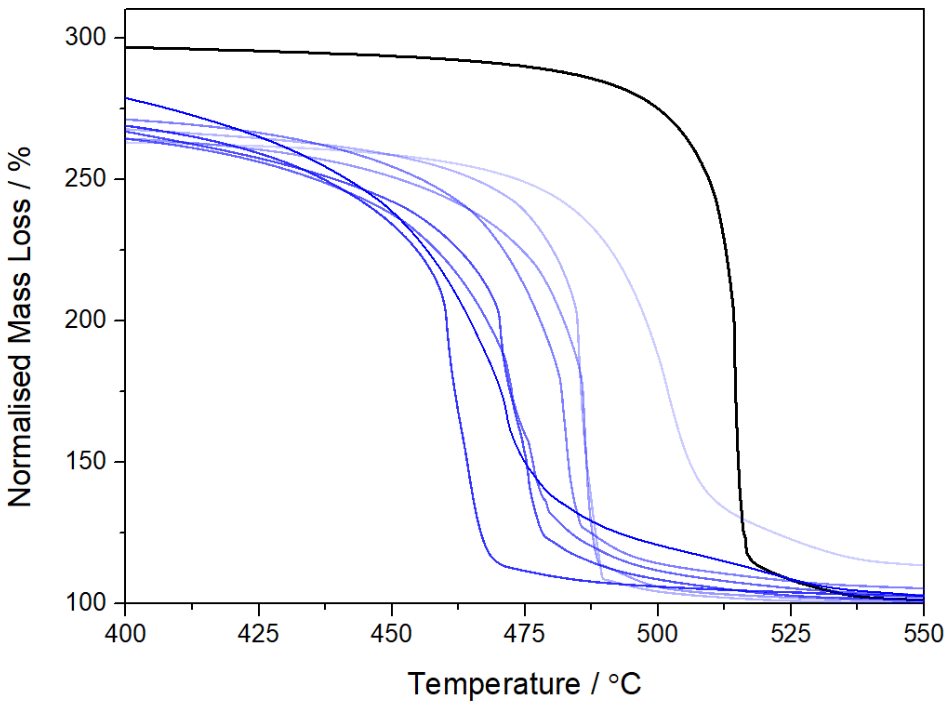

Figure S28: Amplification of TGA profiles of MUV-10 synthesised with increased concentrations of Iso-OH, compared to pristine MUV-10, with residues normalised to 100%. The palatine decrease in thermal stability is observed alongside the decrease in the magnitude of the last decomposition step, attributed to linker and modulator.

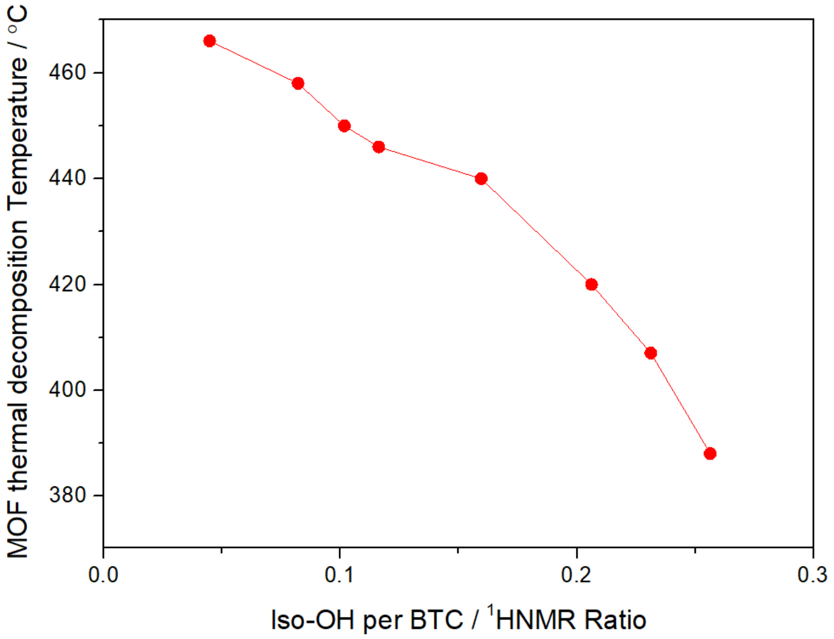

Figure S29: Representation of the framework's decomposition temperature as a function of the modulator incorporated in molar ratio, previously determined by ^{1}HNMR.

Table S9: Data extracted from TGA analysis for the model framework $[TiCaO(H_2O)_w(btc)_x(Iso\text{-}OH)_y(FA)_z(OH)_d]$ using 100% as the end of the last decomposition step (residue). Note that missing linker is 1.33-x and missing linker molar percent (1.33-btc)/btc*100

Sample	RATIO btc/TI	FA	ISO	OH	H_20	Iso per ML	ML%
**20%**	0.918	0.016	0.041	1.147	2.7	0.237	30.946
**33%**	0.988	0.100	0.081	0.774	0.7	0.283	25.726
**43%**	0.839	0.009	0.085	1.303	0.8	0.193	36.918
**50%**	0.656	0.008	0.076	1.873	1.7	0.156	50.711
**60%**	0.659	0.007	0.105	1.805	0.2	0.100	50.445
**67%**	0.779	0.009	0.156	1.342	4.3	0.174	41.432
**83%**	0.600	0.076	0.141	1.843	5.8	0.113	54.924
**91%**	0.473	0.044	0.134	2.271	7.9	0.157	64.457

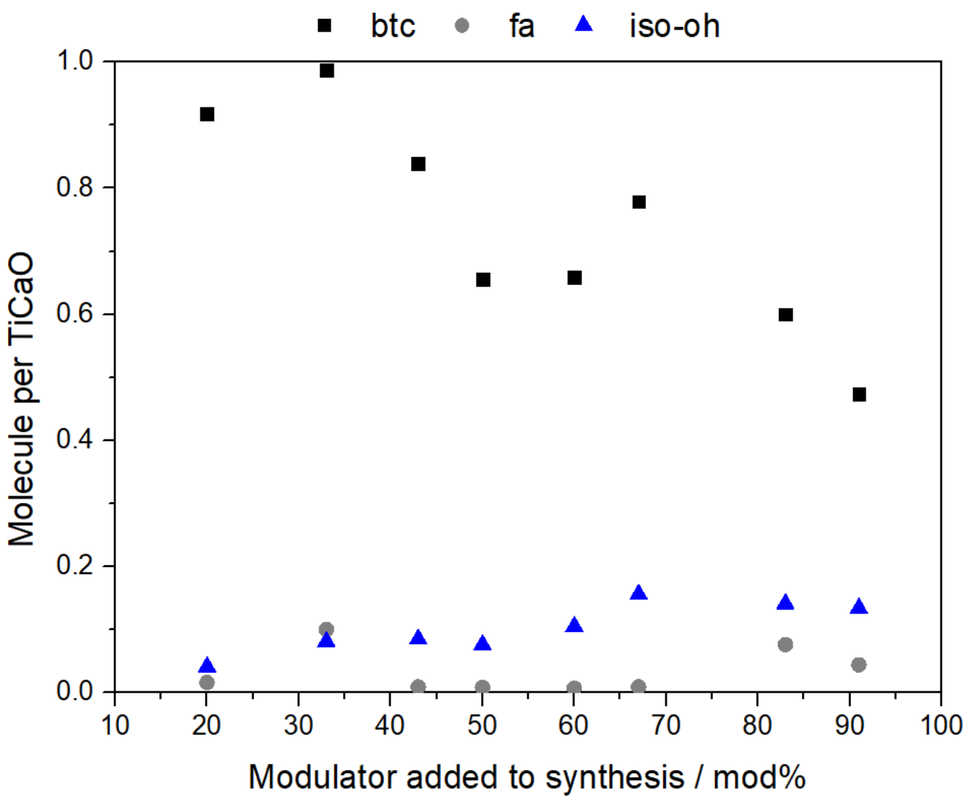

Figure S30: Composition of MUV-10-Iso-OH (H) of molecular formula Iso-$[TiCaO(H_2O)_w(btc)_x(Iso\text{-}OH)_y(FA)_z(OH)_d]$ as a function of the modulator added to synthesis.

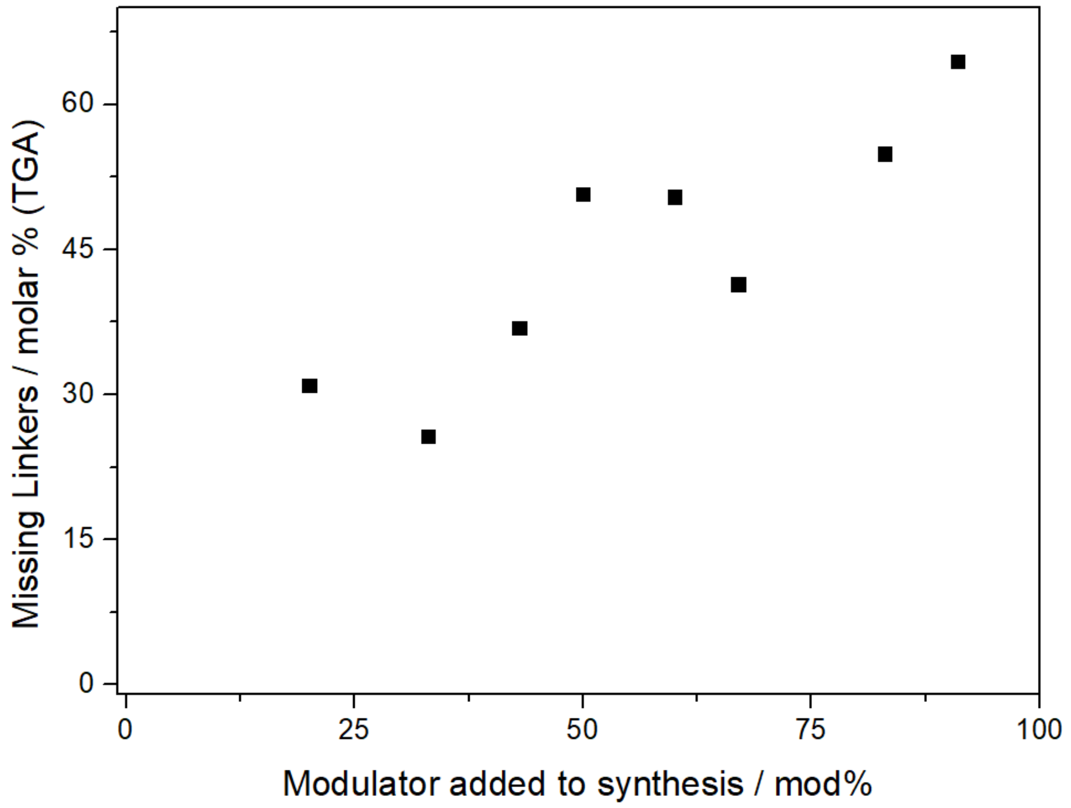

Figure S31: Molar percent of missing linkers a function of the modulator added to synthesis.

S.3.6.b. Thermogravimetric analysis (TGA) of Iso-F modulated samples

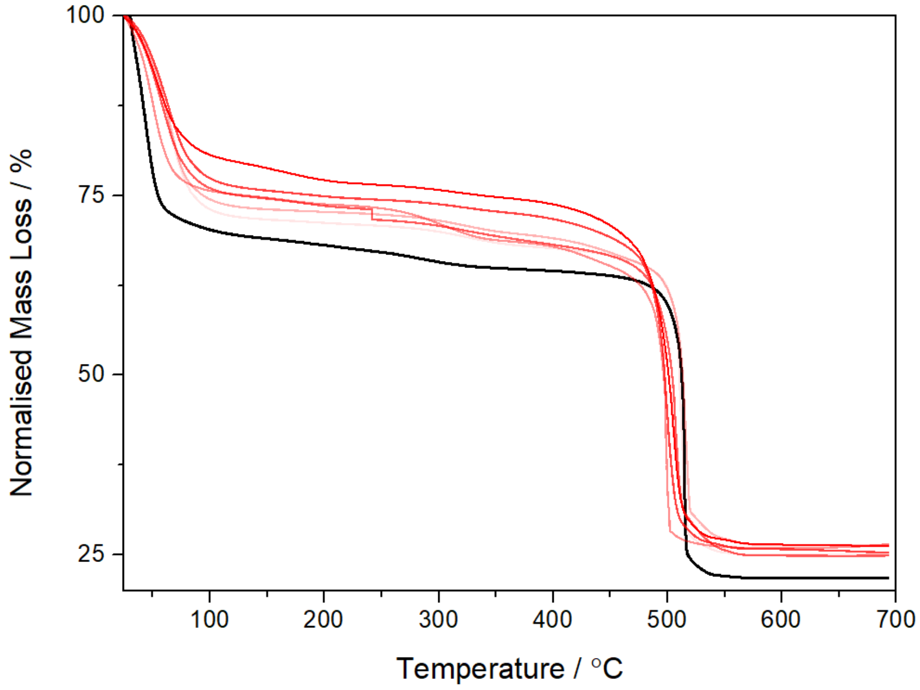

Figure S32: TGA profiles of MUV-10 synthesised with increased concentrations of Iso-F, compared to pristine MUV-10, with the start of the decomposition profiles normalised to 100%. A slight increase in metal content can be appreciated.

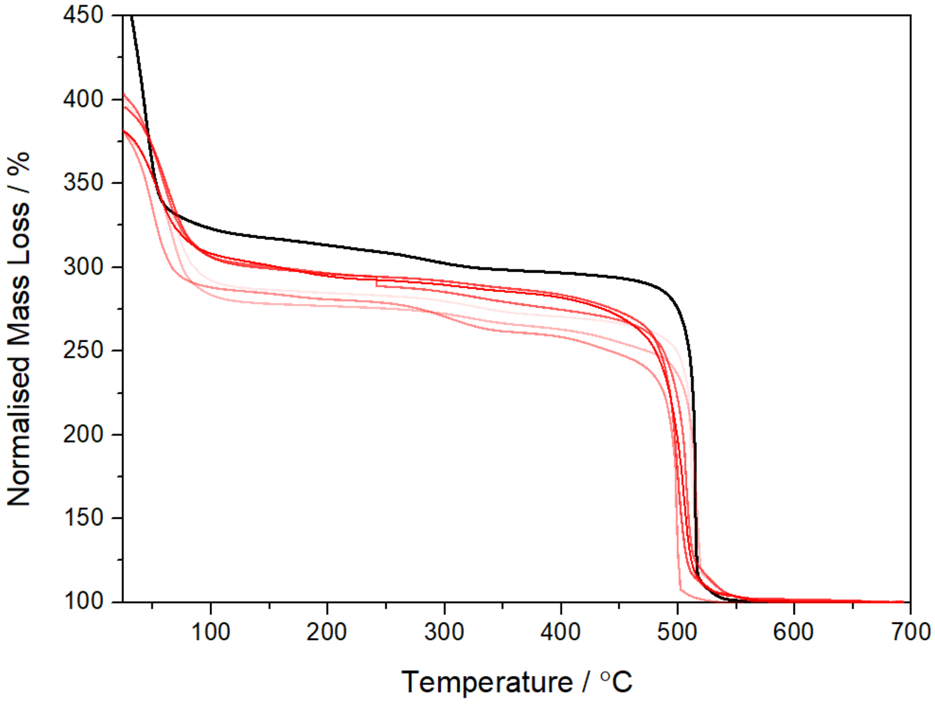

Figure S33: TGA profiles of MUV-10 synthesised with increased concentrations of Iso-F, compared to pristine MUV-10, with the residues normalised to 100%. A decrease in the btc decomposition step is observed, with no decrease in thermal stability.

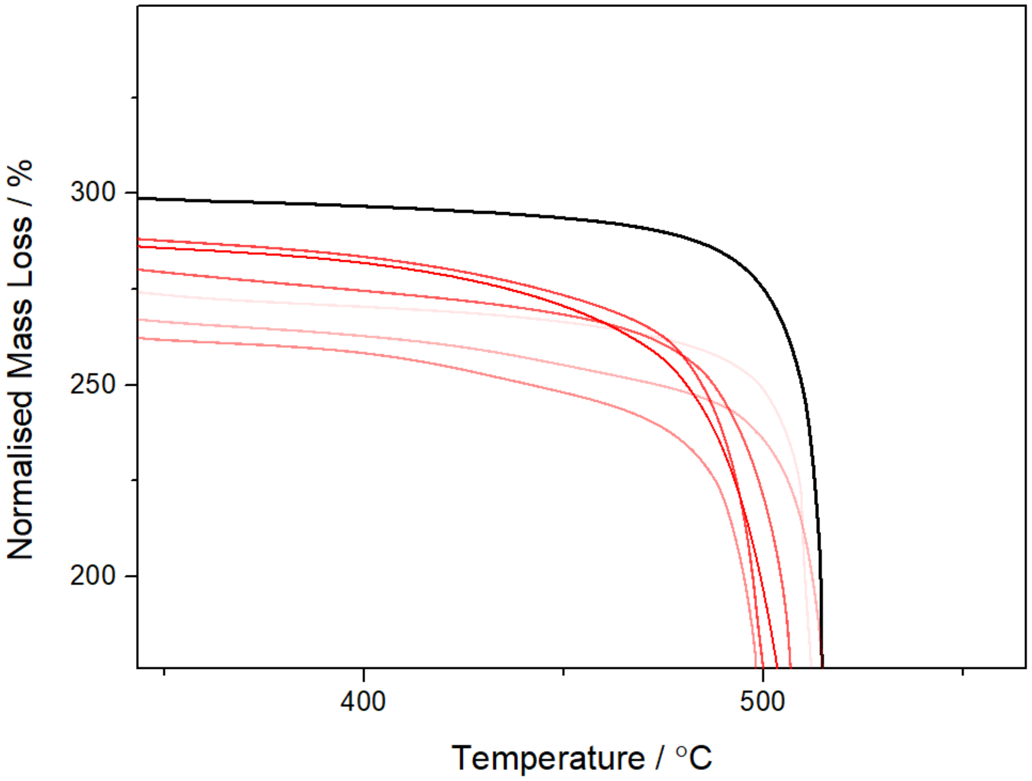

Figure S34: Amplification of TGA profiles of MUV-10 synthesised with increased concentrations of Iso-F, compared to pristine MUV-10, with residues normalised to 100%. No decrease in thermal stability is observed.

Table S10: Data extracted from TGA analysis for the model framework [$TiCaO(H_2O)_w(btc)_x(Iso\text{-}F)_y(FA)_z(OH)_d$] using 100% as the end of the last decomposition step (residue). Note that missing linker is 1.33-x and missing linker molar percent (1.33-btc)/btc*100

Sample	RATIO btc/TI	FA	ISO-F	OH	H_20	ML%	ML/8	Iso/ML
**10%**	1.168	0.033	0.047	0.369	1.6	12.158	0.990	0.289
**20%**	1.116	0.023	0.095	0.439	1.0	16.098	1.305	0.443
**33%**	1.086	0.126	0.119	0.379	1.1	18.366	1.486	0.487
**67%**	1.082	0.164	0.182	0.226	2.0	18.639	1.507	0.734
**83%**	0.961	0.112	0.348	0.310	1.8	27.770	2.236	0.944
**91%**	0.809	0.000	0.523	0.525	1.9	39.138	3.143	1.005

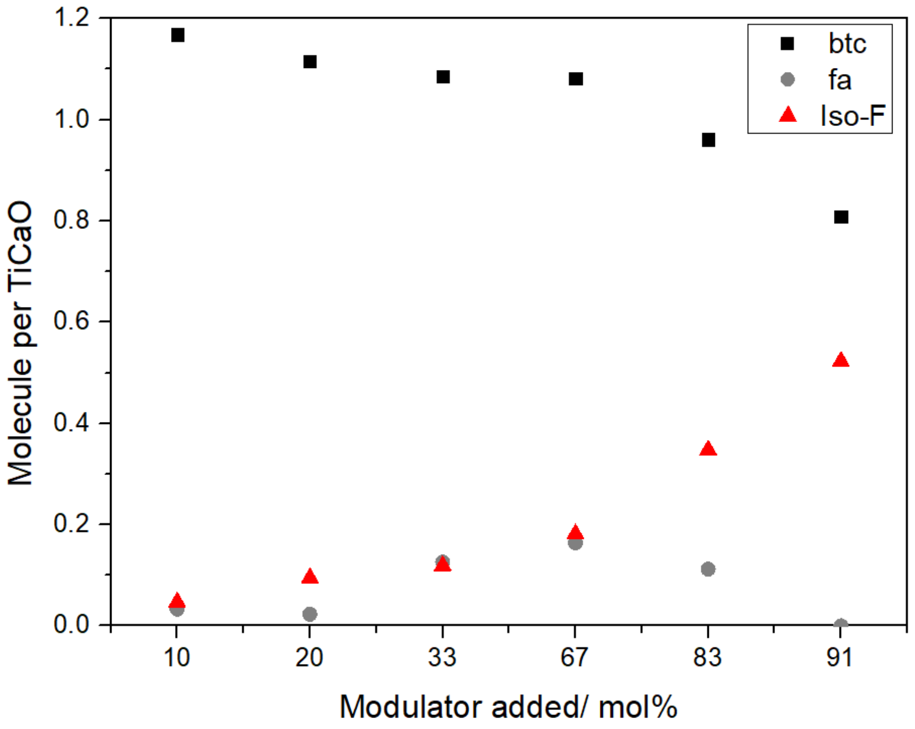

Figure S35: Composition of MUV-10-Iso-F (H) of molecular formula Iso-[TiCaO(H_2O)$_w$(btc)$_x$(Iso-F)$_y$(FA)$_z$(OH)$_d$] as a function of the modulator added to synthesis.

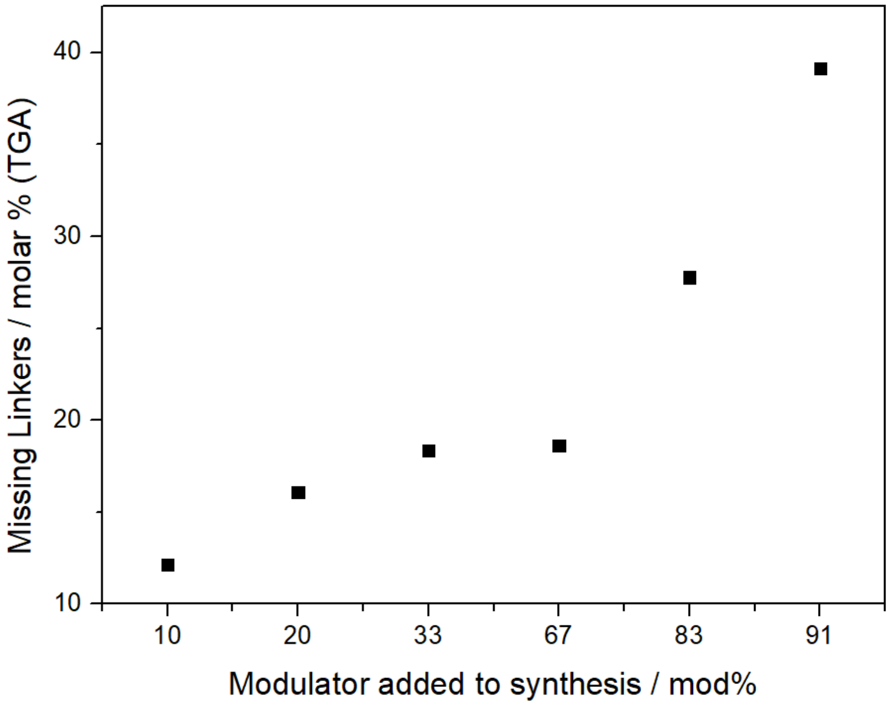

Figure S36: Molar percent of missing linkers a function of the modulator added to synthesis.

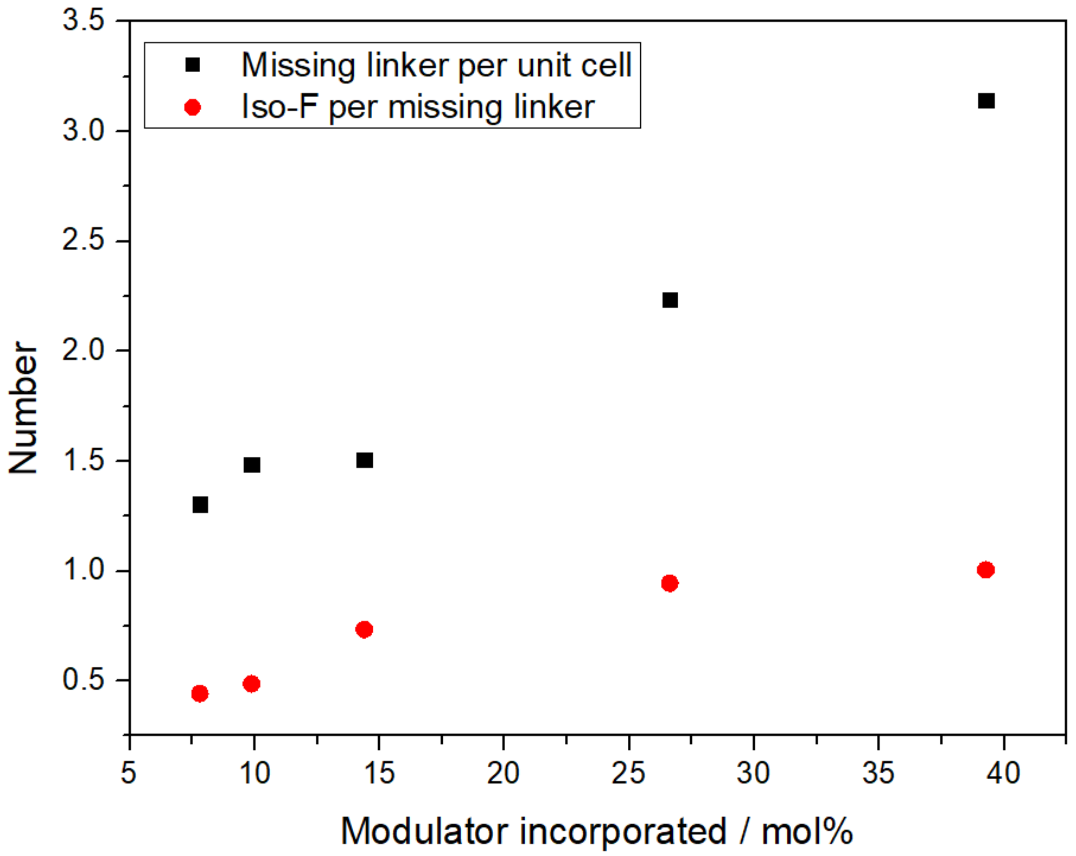

Figure S37: Number of missing linker per unit cell and iso-f per missing linker as a function of the modulator imported in molar percent.

S.3.7. Water and Methanol Stability

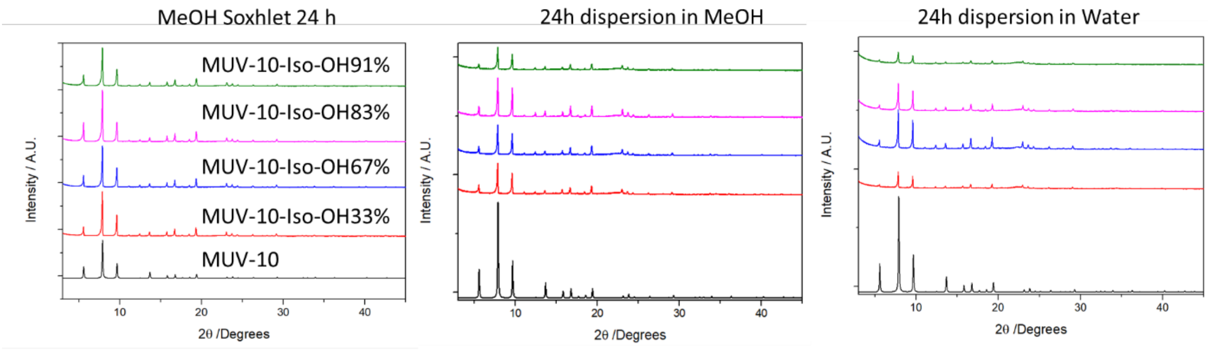

Figure S38: PXRD patterns of the Iso-OH MOFs as synthesised and after MeOH and water dispersion during 24 hours, showing a decrease in crystallinity after water dispersion as the incorporation of Iso-OH increases.

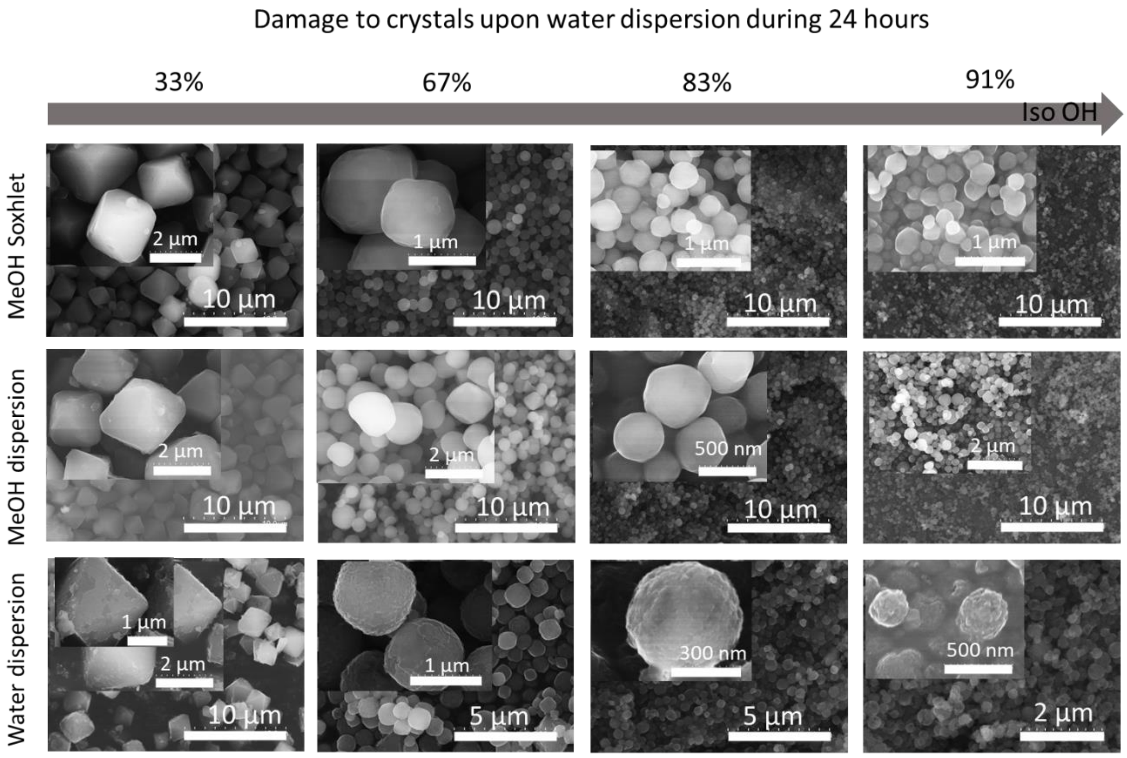

Figure S39: SEM images of the Iso-OH MOFs as synthesised and after MeOH and water dispersion during 24 hours, showing damage to the crystals after water dispersion as the incorporation of Iso-OH increases.

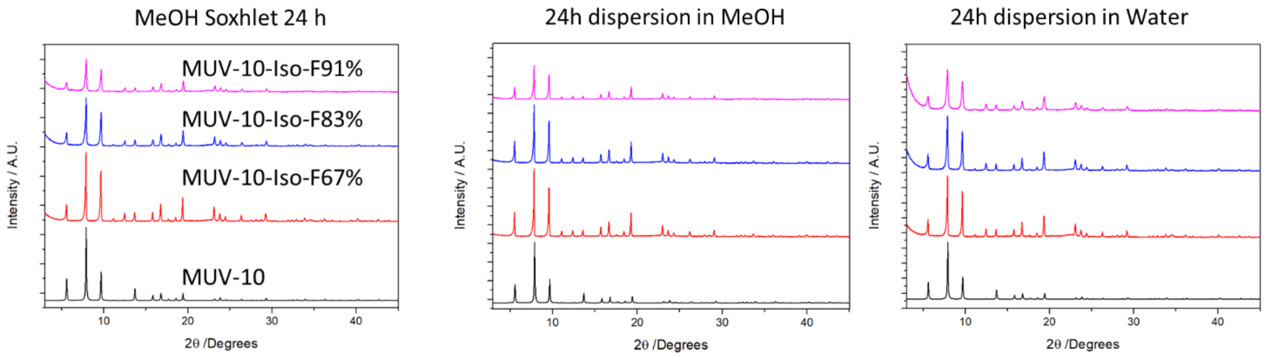

Figure S40: PXRD patterns of the Iso-F MOFs as synthesised and after MeOH and water dispersion during 24 hours, showing no decrease in crystallinity after water dispersion.

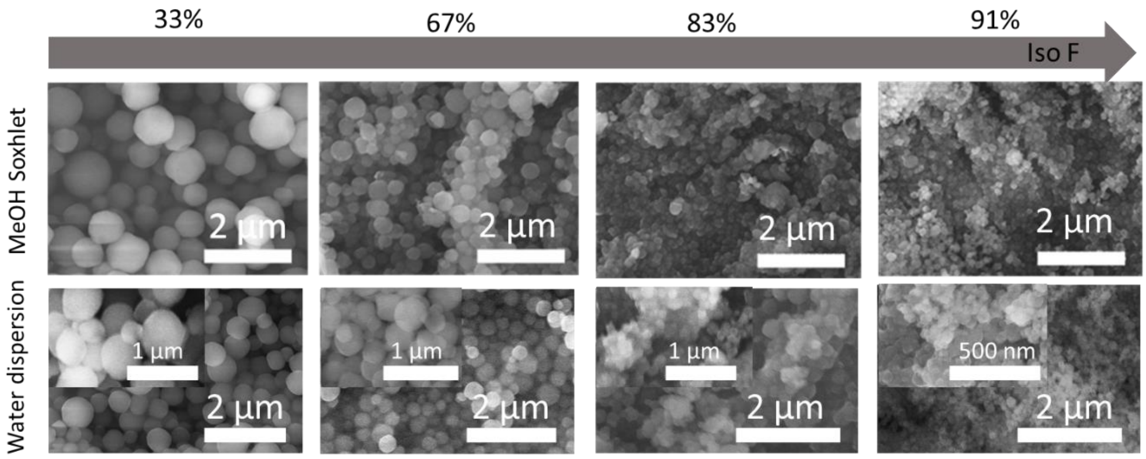

Figure S41: SEM images of the Iso-F MOFs as synthesised and after water dispersion during 24 hours, showing no damage to the crystals after water dispersion as the incorporation of Iso-F increases.

S.3.8. Nitrogen Adsorption and desorption measurements

S.3.8.a. Nitrogen Adsorption and desorption measurements of Iso-OH modulated samples

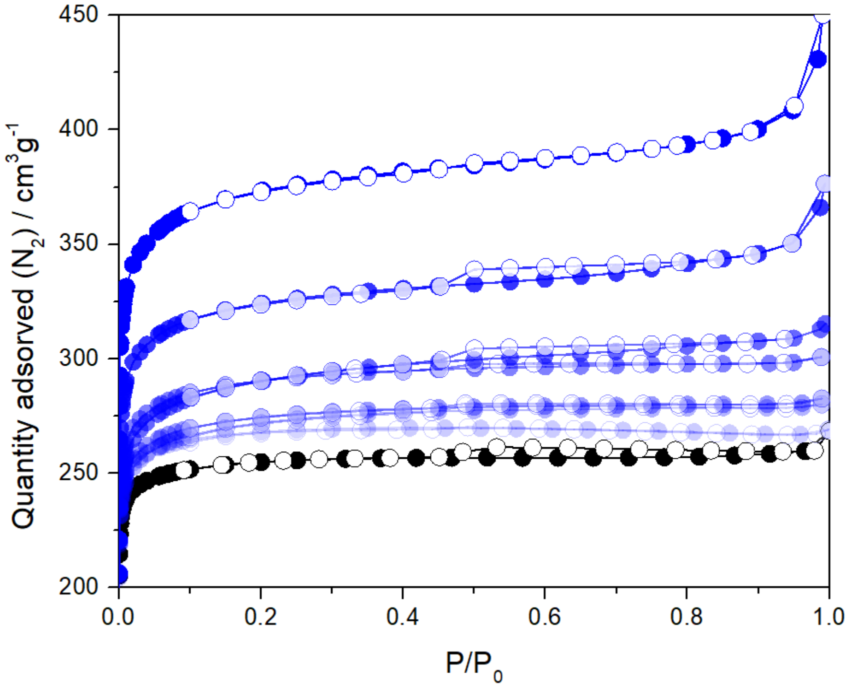

Figure S42: N_2 adsorption and desorption isotherms of MUV-10 synthesised with increased Iso-OH concentration, showing an increase in porosity consistent with modulator addition and subsequent incorporation.

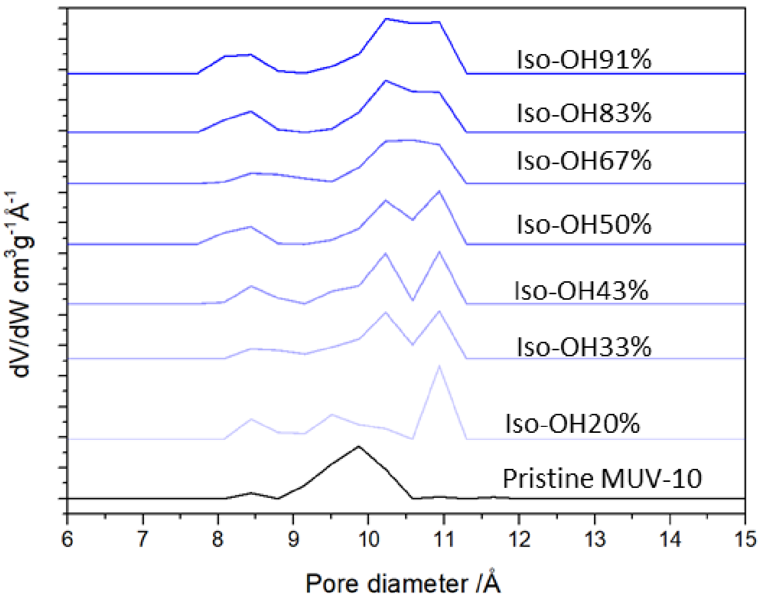

Figure S43: Pore size distributions extracted from the N_2 adsorption and desorption isotherms of MUV-10 increased Iso-OH concentration. Pore size distributions calculated with DFT Cylindrical oxide surface pore model with no regularisation. An increase in the pore sizes is observed consistently with the incorporation of Iso-OH.

Table S11: Tabulated data extracted from N_2 adsorption and desorption measurements of MUV-10 synthesised with increased concentration of Iso-OH showing an increase in surface area, microporosity and total pore volumes.

Sample	S_{BET}	S_{MICRO}	S_{EXT}	V_{MICRO}	V_{MESO}	V_{TOTAL}
**Pristine**	1040	974	66	0.365	0.037	0.402
**20%**	1075	1002	73	0.383	0.031	0.414
**33%**	1072	954	118	0.369	0.063	0.432
**43%**	1083	957	81	0.371	0.064	0.435
**50%**	1091	987	104	0.379	0.059	0.438
**60%**	1152	1033	119	0.398	0.065	0.463
**67%**	1135	975	160	0.379	0.099	0.478
**83%**	1290	1129	161	0.4314	0.105	0.536
**91%**	1472	1348	124	0.524	0.097	0.621

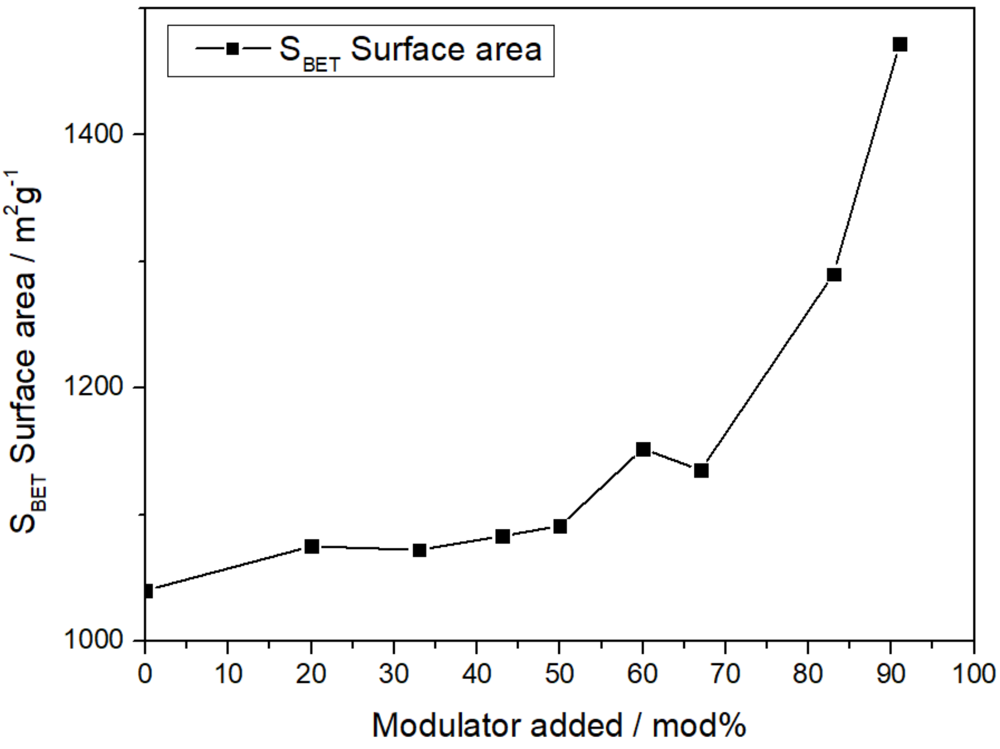

Figure S44: Representation of the BET surface area as a function of the modulator added to synthesis.

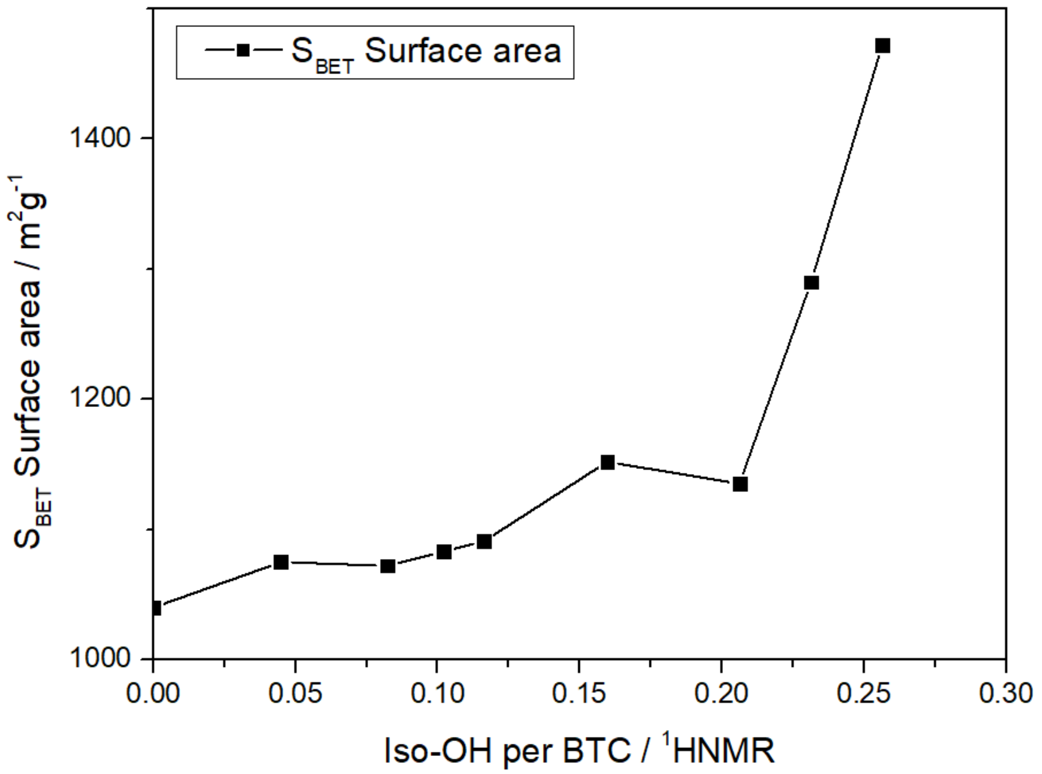

Figure S45: Representation of the BET surface area as a function of the modulator incorporated (molar ratio compared to btc).

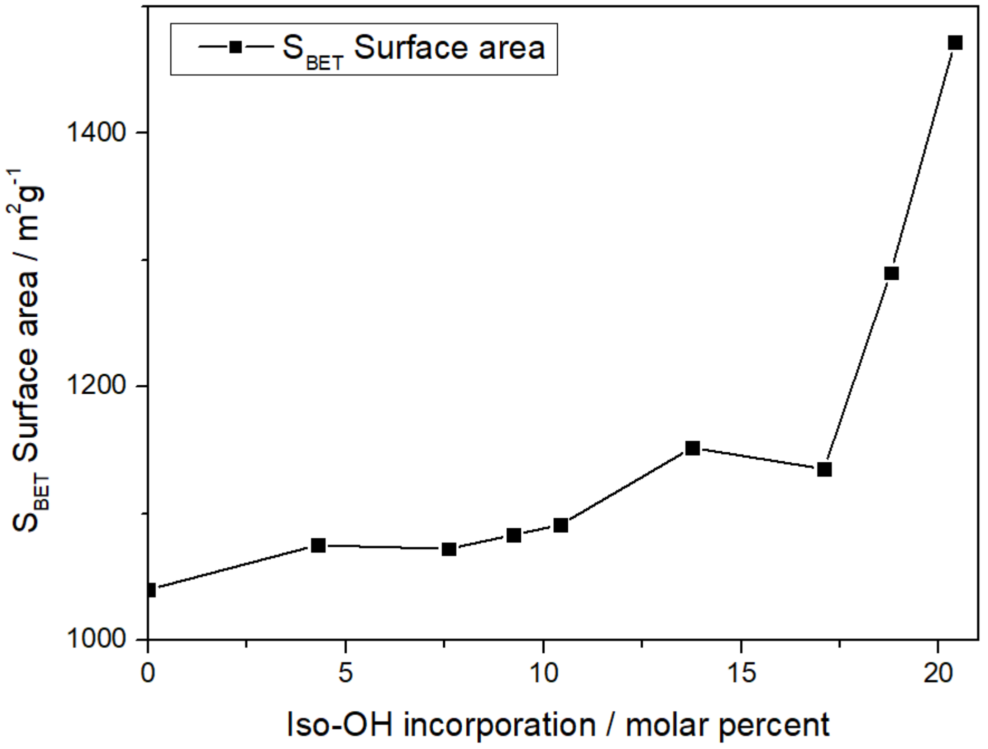

Figure S46: Representation of the BET surface area as a function of the modulator incorporated (molar percent compared to btc)

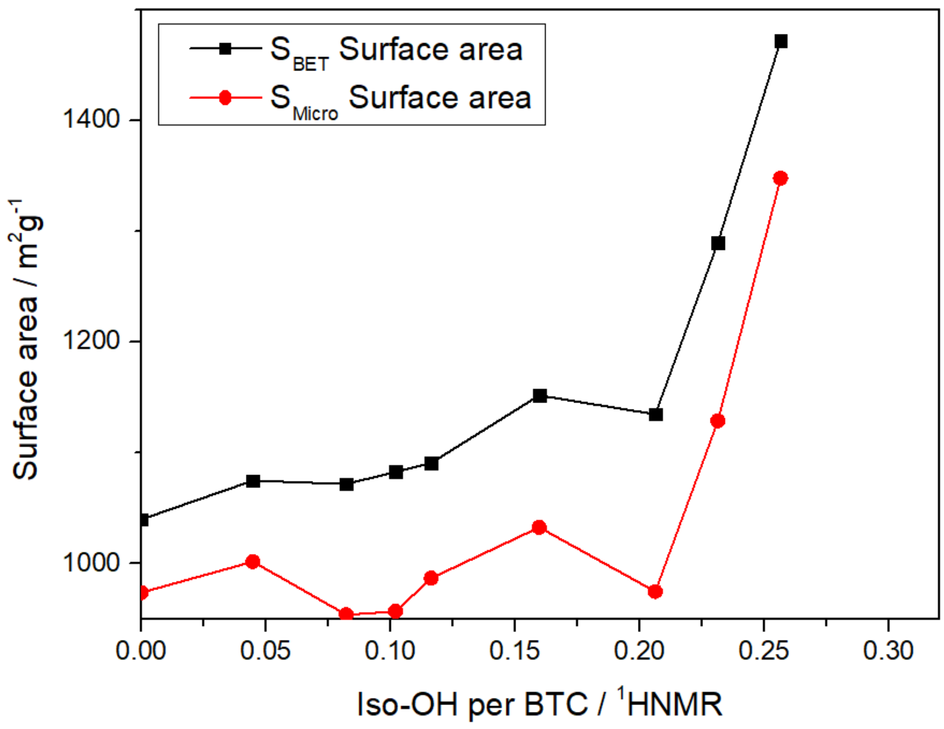

Figure S47: Representation of the BET and micropore surface area as a function of the modulator incorporated (molar ratio compared to btc)

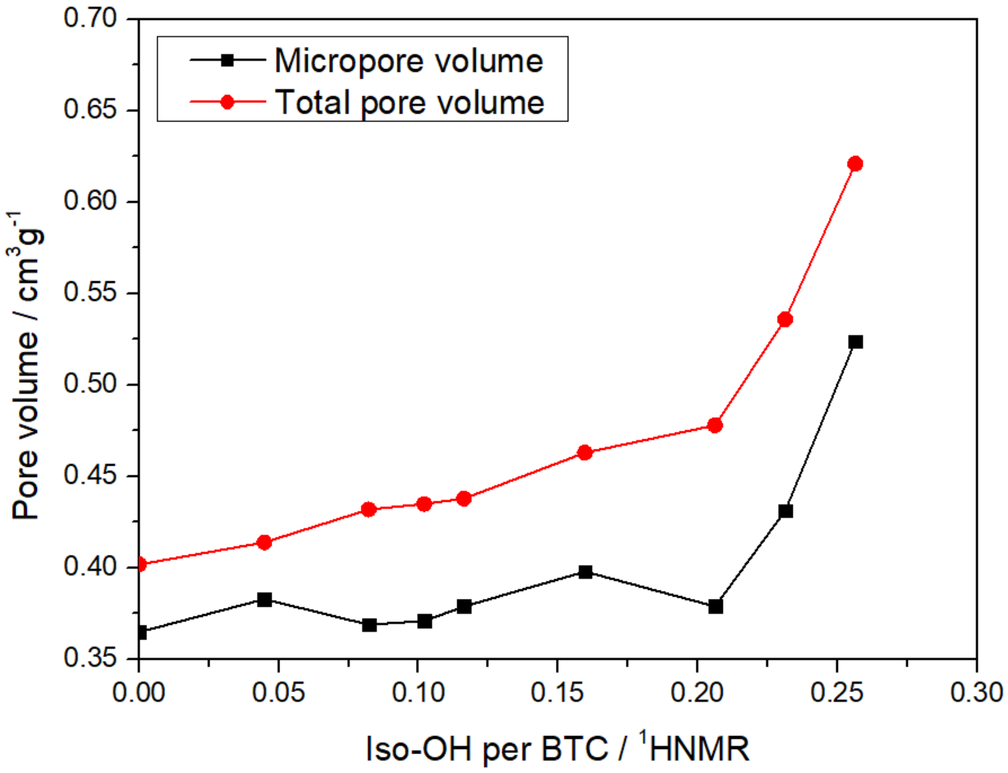

Figure S48: Representation of the micropore and total pore volumes as a function of the modulator incorporated (molar ratio compared to btc)

S.3.8.b. Nitrogen Adsorption and desorption measurements of Iso-F modulated samples

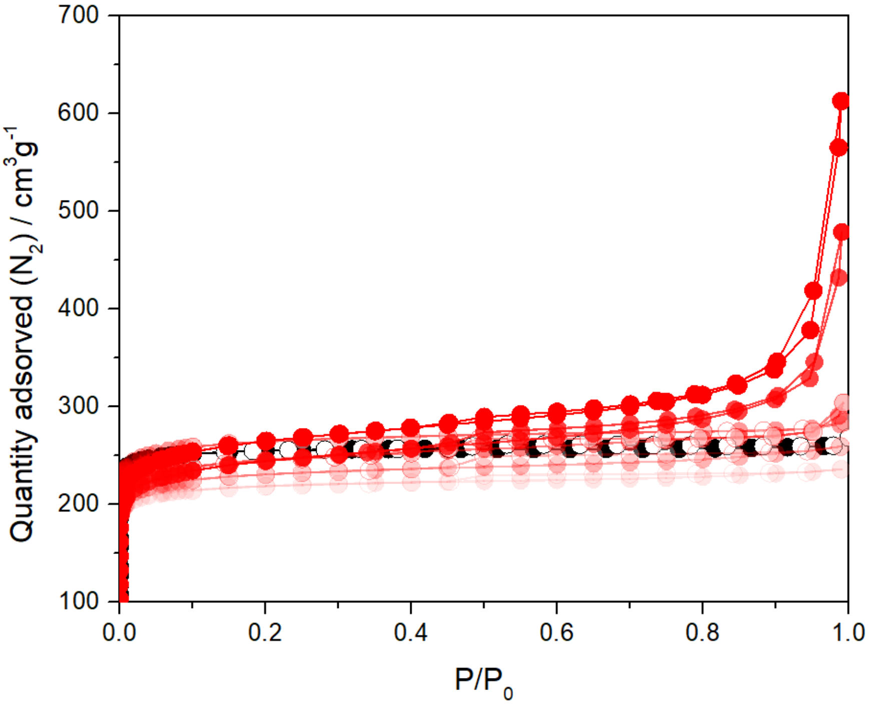

Figure S49: N_2 adsorption and desorption isotherms of MUV-10 synthesised with increased Iso-F concentration, showing an increase in porosity consistent with modulator addition and subsequent incorporation.

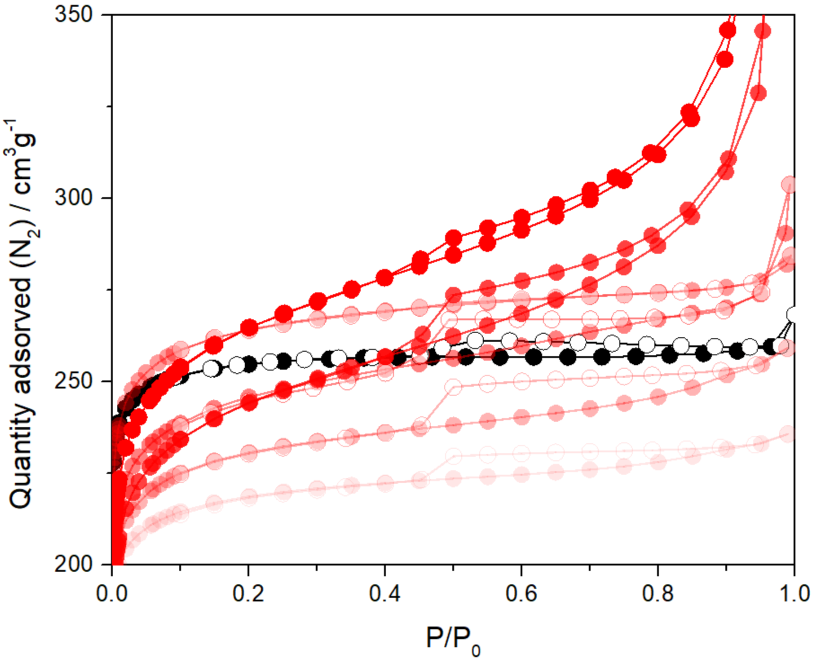

Figure S50: N_2 adsorption and desorption isotherms of MUV-10 synthesised with increased Iso-F concentration, showing an increase in porosity consistent with modulator addition and subsequent incorporation.

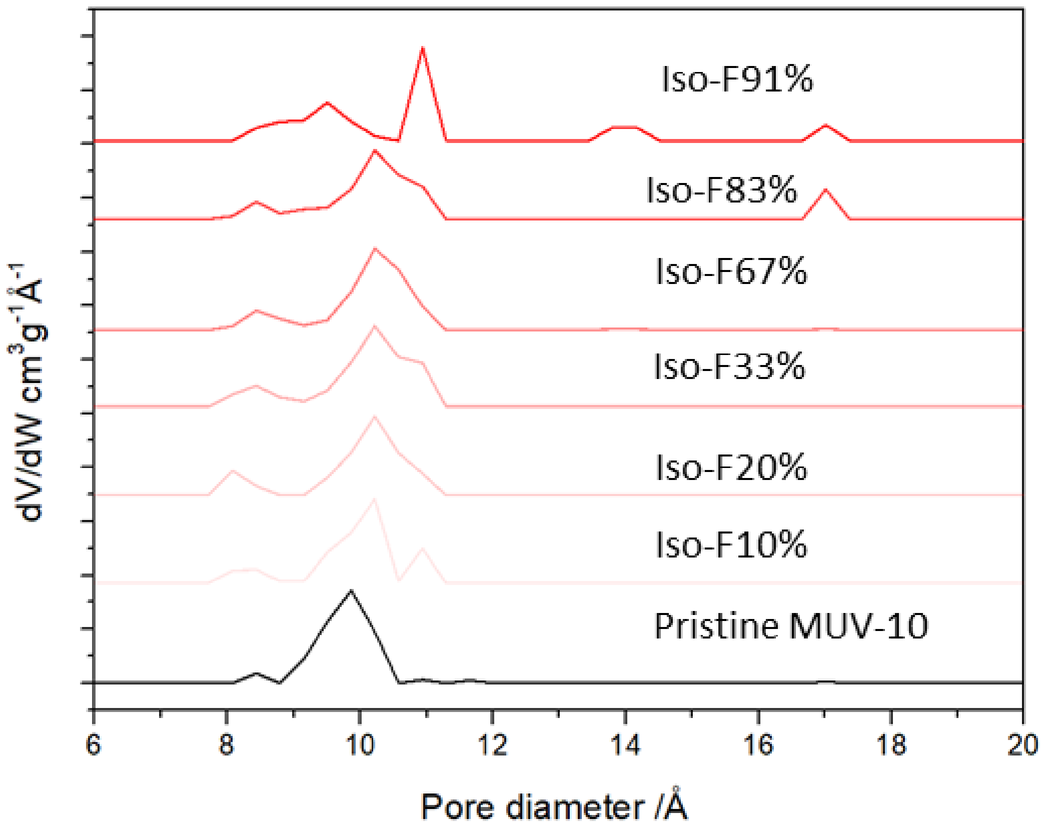

Figure S51: Pore size distributions extracted from the N_2 adsorption and desorption isotherms of MUV-10 increased Iso-F concentration. Pore size distributions calculated with DFT Cylindrical oxide surface pore model with no regularisation. An increase in the pore sizes is observed consistently with the incorporation of Iso-F.

Table S12: Tabulated data extracted from N_2 adsorption and desorption measurements of MUV-10 synthesised with increased concentration of Iso-F showing maintained surface area and microporosity, alongside an increase in total pore volumes.

Sample	S_{BET}	S_{MICRO}	S_{EXT}	V_{MICRO}	V_{MESO}	V_{TOTAL}
**Pristine**	1040	974	66	0.365	0.037	0.402
**10%**	876	786	90	0.300	0.06	0.360
**20%**	910	789	121	0.304	0.087	0.391
**33%**	1051	930	121	0.356	0.072	0.428
**67%**	958	803	155	0.313	0.114	0.427
**83%**	929	710	219	0.282	0.228	0.510
**91%**	1005	773	232	0.305	0.219	0.524

S.4. Photocatalysis

Methodology:

A quartz photoreactor attached with a regular manometer was loaded with 20 mL of a H_2O:methanol mixture (4:1 v/v) in which 20 mg of MOF (1mg/mL) was prior dispersed in methanol. The reactor was purged with an Ar flow for 10-15 minutes, after which it was pressurized to 1 bar. The photocatalytic reactions were carried out by employing a focalized UV-Vis light generated by a 300 W Hg-Xe lamp (0.15 W/cm^2). All reactions were stopped after 24 hours of irradiation. No external heat source was employed. The evolution of H_2 inside the photoreactor was monitored by the employ of an Agilent 490 MicroGC (MolSieve 5Å column, Ar carrier gas) with a TC detector. The system was calibrated prior to the experiments by injecting gas mixtures of known v%. Data quantification was carried out by employ of this calibration.

Table S13: Tabulated data for the photocatalytic H_2 evolution of the unmodulated 2µm MUV-10.

**Time (hours)**	**H_2 production (μmol/g)**
0.5	1.62 (±2.89)
1.5	10.98 (±2.08)
3	28.59 (±7.53)
6	51.74 (±5.75)
24	309.45 (±20.35)

Table S14: Tabulated data for the photocatalytic H_2 evolution of the unmodulated 200 nm MUV-10.

**Time (hours)**	**H_2 production (μmol/g)**
0.5	3.16 (±2.27)
1.5	8.21 (±2.35)
3	25.26 (±3.43)
6	69.88 (±6.09)
24	371.01 (±16.35)

S.4.1.a. Photocatalysis of MUV-10-Iso-OH

Table S15: Tabulated data for the photocatalytic H_2 evolution of MUV-10-Iso-OH 20%.

Time (hours)	H_2 production (μmol/g)
0.5	1.99 (±1.68)
1.5	7.18 (±3.25)
3	23.06 (±2.42)
6	80.53 (±11.21)
24	527.16 (±23.53)

Table S16: Tabulated data for the photocatalytic H_2 evolution of MUV-10-Iso-OH 33%.

Time (hours)	H_2 production (μmol/g)
0.5	2.99 (±1.77)
1.5	10.32 (±3.93)
3	25.5 (±8.99)
6	79.71 (±12.18)
24	583.27 (±41.84)

Table S17: Tabulated data for the photocatalytic H_2 evolution of MUV-10-Iso-OH 43%.

Time (hours)	H_2 production (μmol/g)
0.5	2.66 (±0.69)
1.5	10.57 (±2.38)
3	48.38 (±12.83)
6	166.75 (±32.92)
24	703.01 (±52.231)

Table S18: Tabulated data for the photocatalytic H_2 evolution of MUV-10-Iso-OH 50%.

**Time (hours)**	**H_2 production (μmol/g)**
0.5	2.99 (±2.59)
1.5	10.29 (±4.99)
3	29.76 (±6.06)
6	82.85 (±15.31)
24	470.86 (±34.24)

Table S19: Tabulated data for the photocatalytic H_2 evolution of MUV-10-Iso-OH 60%.

**Time (hours)**	**H_2 production (μmol/g)**
0.5	1.48 (±0.54)
1.5	7.03 (±1.04)
3	25.17 (±3.65)
6	89.79 (±10.93)
24	435.73 (±30.02)

Table S20: Tabulated data for the photocatalytic H_2 evolution of MUV-10-Iso-OH 67%.

**Time (hours)**	**H_2 production (μmol/g)**
0.5	1.35 (±1.18)
1.5	4.45 (±4.87)
3	13.86 (±8.32)
6	58.41 (±8.17)
24	373.08 (±26.83)

Table S21: Tabulated data for the photocatalytic H_2 evolution of MUV-10-Iso-OH 83%.

**Time (hours)**	**H_2 production (μmol/g)**
0.5	2.21 (±0.23)
1.5	8.27 (±1.56)
3	18.68 (±1.69)
6	48.04 (±5.19)
24	330.83 (±29.41)

Table S22: Tabulated data for the photocatalytic H_2 evolution of MUV-10-Iso-OH 91%.

**Time (hours)**	**H_2 production (μmol/g)**
0.5	1.66 (±1.75)
1.5	5.29 (±1.17)
3	20.12 (±6.85)
6	55.97 (±8.87)
24	322.06 (±27.158)

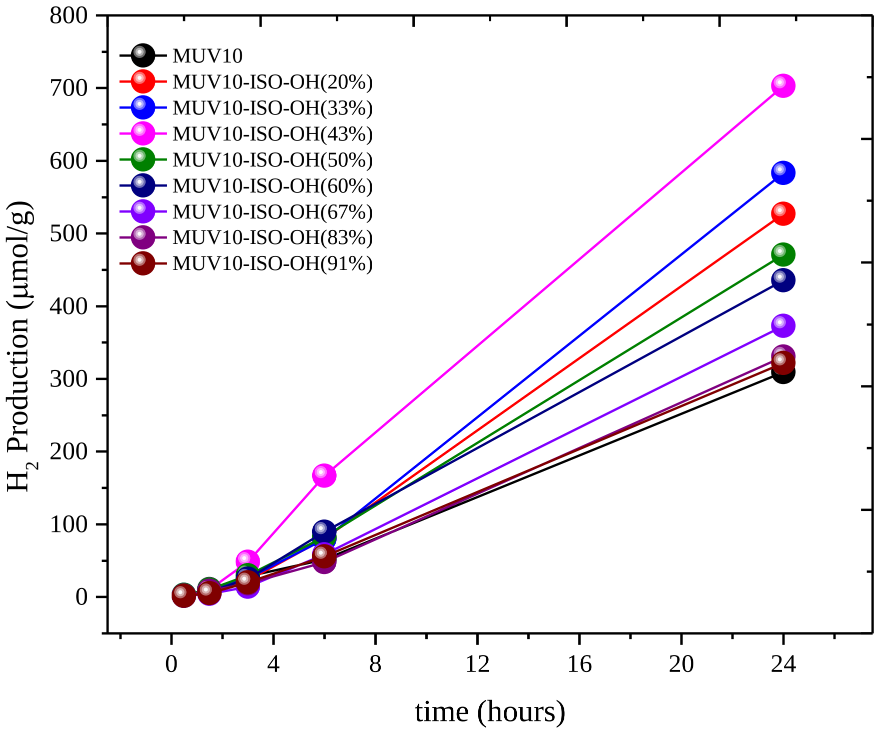

Figure S52: Time evolution profiles of the photocatalytic H_2 evolution from MUV-10-Iso-OH samples.

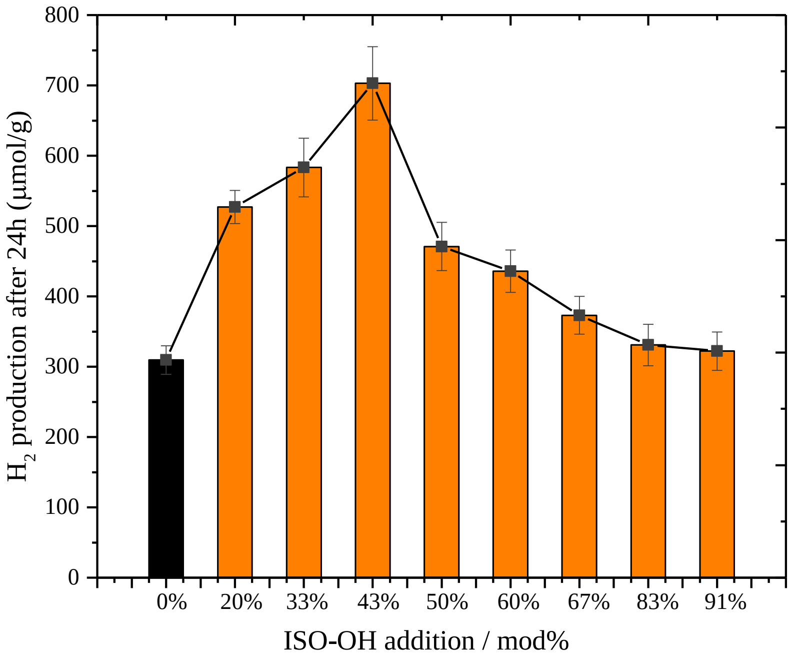

Figure S53: Representation of the photocatalytic H_2 evolution from MUV-10-Iso-OH samples after 24 hours of irradiation.

S.4.1.b. Photocatalysis of MUV-10-Iso-F

Table S23: Tabulated data for the photocatalytic H_2 evolution of MUV-10-Iso-F 10%.

**Time (hours)**	**H_2 production (μmol/g)**
0.5	1.78 (±1.09)
1.5	6.11 (±0.7)
3	18.84 (±3.18)
6	54.38 (±10.77)
24	308.45 (±20.73)

Table S24: Tabulated data for the photocatalytic H_2 evolution of MUV-10-Iso-F 20%.

**Time (hours)**	**H_2 production (μmol/g)**
0.5	1.59 (±0.38)
1.5	5.45 (±1.97)
3	20.01 (±5.73)
6	55.14 (±7.79)
24	331.9 (±26.35)

Table S25: Tabulated data for the photocatalytic H_2 evolution of MUV-10-Iso-F 33%.

**Time (hours)**	**H_2 production (μmol/g)**
0.5	1.85 (±0.74)
1.5	9.19 (±0.54)
3	28.63 (±5.87)
6	79.01 (±10.52)
24	426.66 (±31.31)

Table S26: Tabulated data for the photocatalytic H_2 evolution of MUV-10-Iso-F 67%.

**Time (hours)**	**H_2 production (μmol/g)**
0.5	1.47 (±2.88)
1.5	12.64 (±4.65)
3	38.81 (±9.56)
6	101.92 (±13.01)
24	718.0.4 (±62.31)

Table S27: Tabulated data for the photocatalytic H_2 evolution of MUV-10-Iso-F 83%.

**Time (hours)**	**H_2 production (μmol/g)**
0.5	2.39 (±0.89)
1.5	8.95 (±2.63)
3	24.48 (±3.08)
6	95.31 (±8.62)
24	776.27 (±64.32)

Table S28: Tabulated data for the photocatalytic H_2 evolution of MUV-10-Iso-F 91%.

**Time (hours)**	**H_2 production (μmol/g)**
0.5	5.28 (±3.57)
1.5	19.81 (±6.44)
3	68.3 (±16.22)
6	240.03 (±21.33)
24	1297.99 (±107.21)

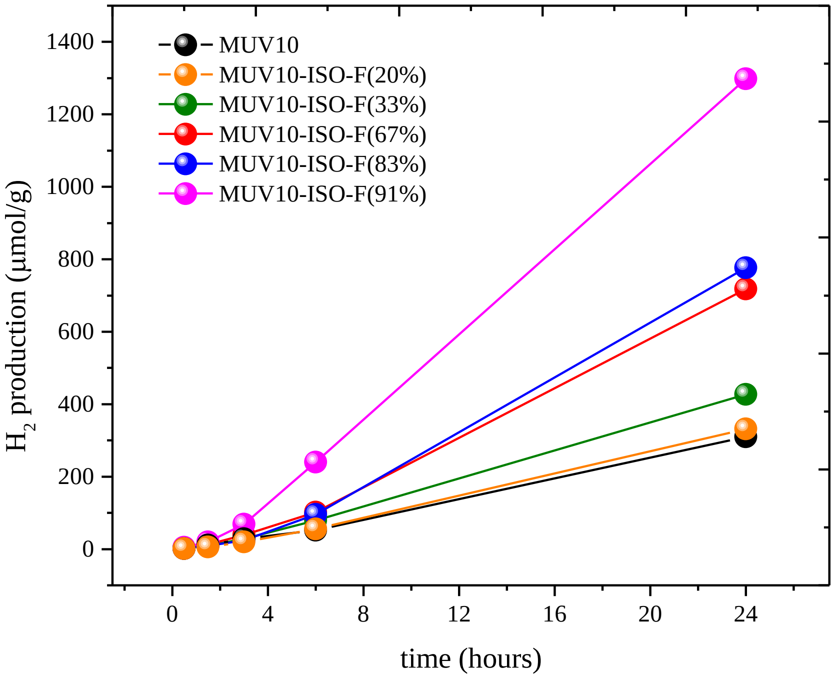

Figure S54: Time evolution profiles of the photocatalytic H_2 evolution from MUV-10-Iso-F samples.

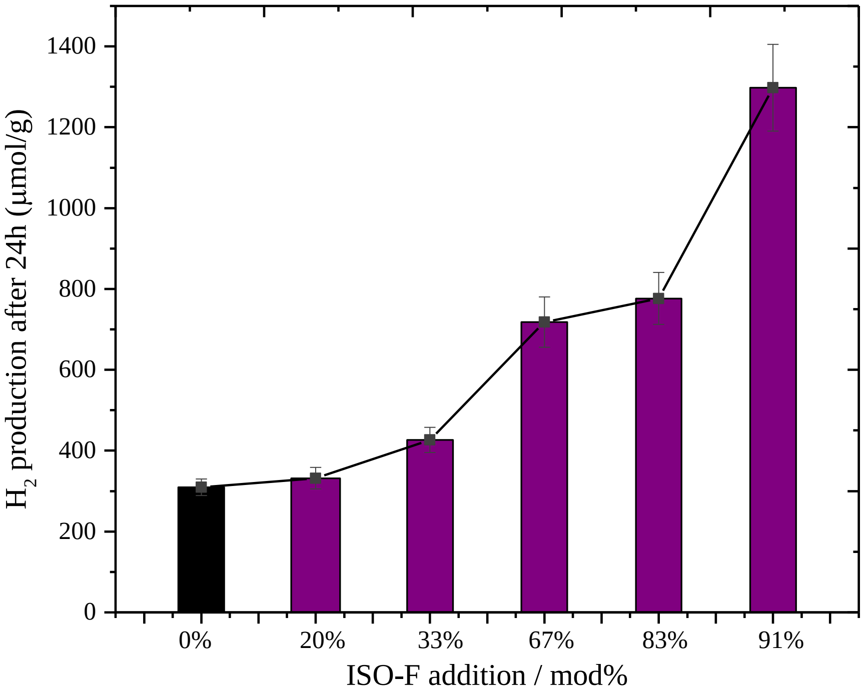

Figure S55: Representation of the photocatalytic H_2 evolution from MUV-10-Iso-F samples after 24 hours of irradiation.

S.4.2. Photocalytic decarboxylation

The CO_2 evolution of the samples was calculated using the maximum amount of possible CO_2 released from the samples decarboxylation based on the composition obtained by thermal analysis (See Section S.3.6).

$$Number\ of\ CO2\ molecules = 3 * BTC + 2 * Iso - X + FA$$

Then, the mass of CO_2 in the 20 mg of samples employed during the photocatalytic studies is calculated, and the mass of CO_2relases is normalised to that value.

Table S29: Tabulated data for the CO_2 evolution of MUV-10 pristine samples in µmol and normalised to the number of carboxylates present in the samples

Time (h)	MUV10 2µm µmol	MUV-10 200 nm µmol	MUV10 2µm %	MUV-10 200 nm %
**0.50**	1.50	2.54	0.80	1.50
**1.50**	1.71	4.13	0.91	2.44
**3.00**	2.46	9.99	1.31	5.90
**6.00**	2.58	16.66	1.37	9.84
**24.00**	7.66	31.80	4.07	18.78

Table S30: Tabulated data for the CO_2 evolution of MUV-10-Iso-OH in µmol.

Time (h)	Iso-OH (20)	Iso-OH (33)	Iso-OH (43)	Iso-OH (50)	Iso-OH (60)	Iso-OH (67)	Iso-OH (83)	Iso-OH (91)
**0.50**	2.27	1.05	3.44	3.49	2.67	4.21	2.76	3.40
**1.50**	2.33	2.74	12.49	13.22	14.00	14.35	11.84	11.41
**3.00**	2.62	5.66	24.02	21.13	25.86	24.37	24.36	19.37
**6.00**	3.63	10.11	32.16	35.36	35.19	42.23	33.14	31.16
**24.00**	17.60	27.22	32.42	41.95	43.39	45.46	44.16	46.05

Table S31: Tabulated data for the CO_2 evolution of MUV-10-Iso-OH in %CO_2 released.

Time (h)	Iso-OH (20)	Iso-OH (33)	Iso-OH (43)	Iso-OH (50)	Iso-OH (60)	Iso-OH (67)	Iso-OH (83)	Iso-OH (91)
**0.5**	1.31	0.56	2.07	2.44	1.84	2.59	1.93	2.78
**1.5**	1.34	1.47	7.52	9.26	9.65	8.83	8.28	9.31
**3**	1.51	3.05	14.46	14.81	17.83	14.98	17.04	15.81
**6**	2.09	5.44	19.36	24.77	24.27	25.96	23.18	25.43
**24**	10.16	14.65	19.52	29.39	29.92	27.95	30.89	37.58

Table S32: Tabulated data for the CO_2 evolution of MUV-10-Iso-F in µmol.

Time (h)	MUV10	Iso-F (20)	Iso-F (33)	Iso-F (67)	Iso-F (83)	Iso-F(91)
**0.5**	1.50	5.08	5.97	7.15	5.19	6.27
**1.5**	1.71	19.21	19.34	18.67	12.87	7.59
**3**	2.46	31.21	32.53	29.09	22.18	24.46
**6**	2.58	31.97	34.55	33.36	33.71	36.26
**24**	7.66	36.40	39.10	39.23	38.55	42.33

Table S33: Tabulated data for the CO_2 evolution of MUV-10-Iso-F in %CO_2 released.

Time (h)	MUV10	Iso-F (20)	Iso-F (33)	Iso-F (67)	Iso-F (83)	Iso-F(91)
**0.5**	0.80	2.73	3.15	3.88	2.87	3.70
**1.5**	0.91	10.32	10.20	10.12	7.13	4.48
**3**	1.31	16.77	17.16	15.77	12.30	14.44
**6**	1.37	17.18	18.22	18.09	18.69	21.42
**24**	4.07	19.56	20.62	21.27	21.37	25.00

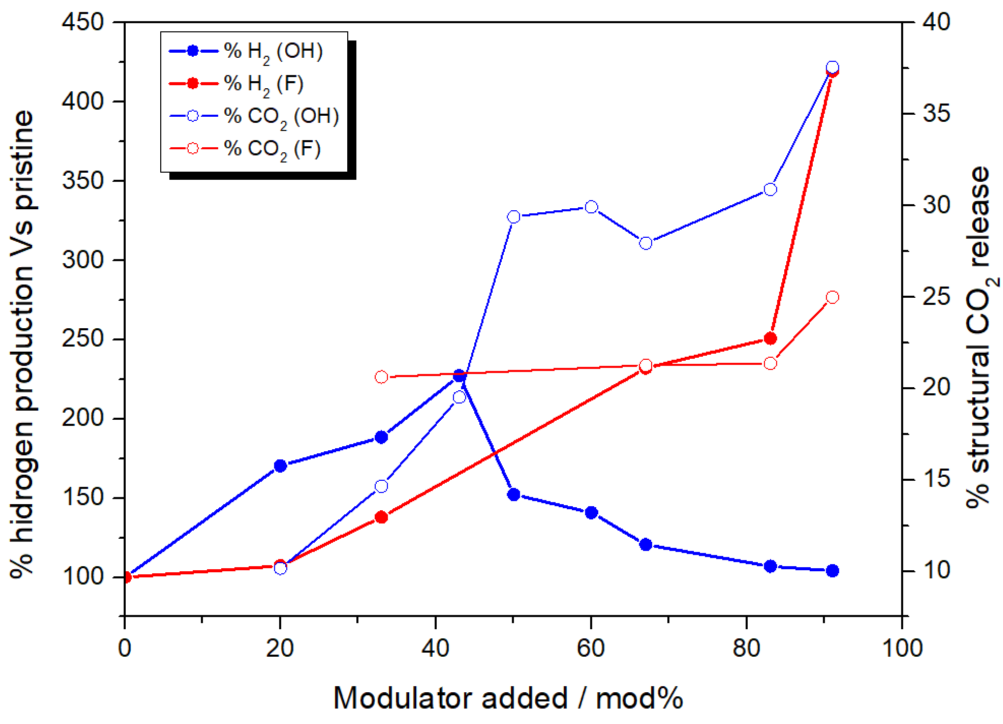

Figure S56: Representation of the H_2 and CO_2 evolution from MUV-10-Iso-OH samples after 24 hours. CO_2 evolution is indicative of the photodecarboxylation of the framework. [2]

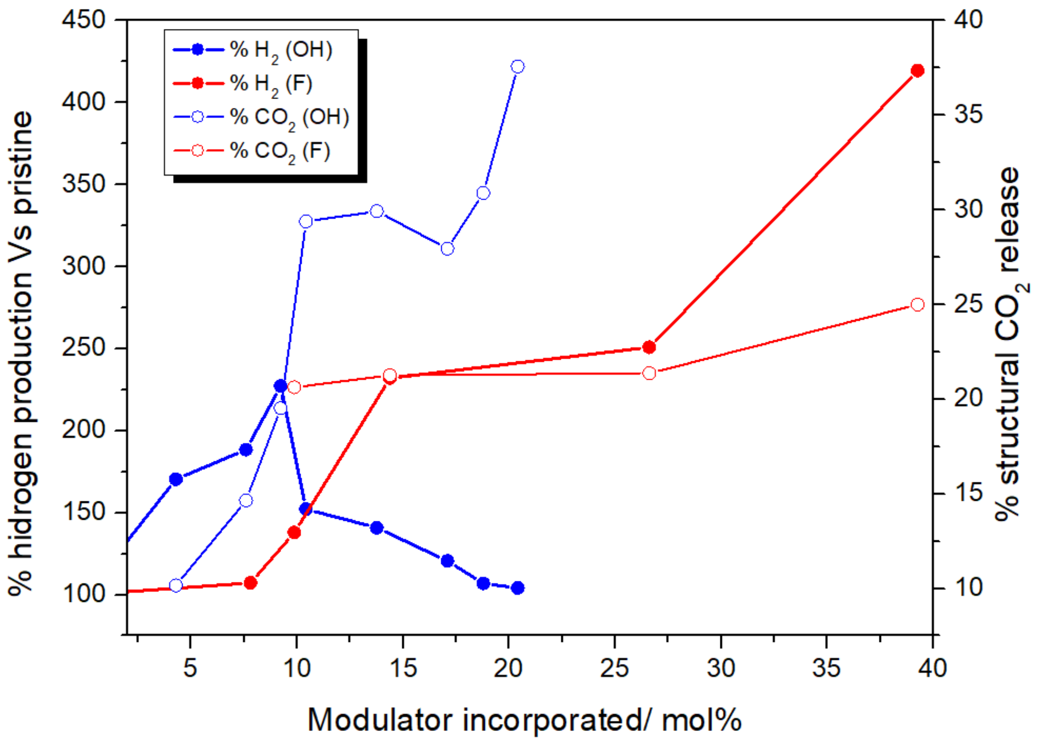

Figure S57: Representation of the H_2 and CO_2 evolution from MUV-10-Iso-F samples after 24 hours.

S.4.3. Photocurrent experiments

Photoelectrodes were fabricated by photocatalyst deposition on transparent conductive supports of fluorine-doped tin oxide (FTO) in identical amounts. First, photocatalysts slurries were prepared by materials dispersion in EtOH (10 mg in 200 μL) containing 50 μL of commercial Nafion dispersion (5 wt.%). 30 μL of the as-prepared slurries were deposited on the FTO surface (1.5 x 1 cm^2), and allowed for solvent evaporation at 40 ^oC overnight. The prepared photoelectrodes were used as working electrodes, while Pt wire and $Ag/AgCl_{Sat.}$ were used as counter electrodes and reference electrodes, respectively. The employed electrolyte was 0.1M $LiClO_4$ acetonitrile solution. Four consecutive cycles of 60 s were carried out switching on and off UV-vis light from a Xe lamp at 1080 W/m^2 while photocurrent values were acquired in a potentiostat.Versatat 3 (AMETEK).

S.4.4. Band Gap

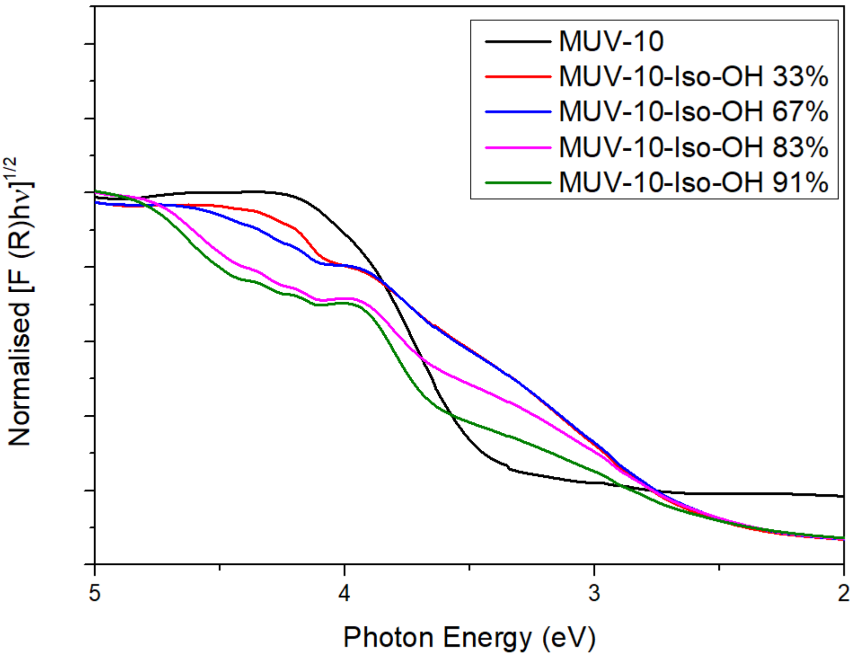

Figure S58: Kubelka-Munk function of MUV-10 and MUV-10-OH samples.

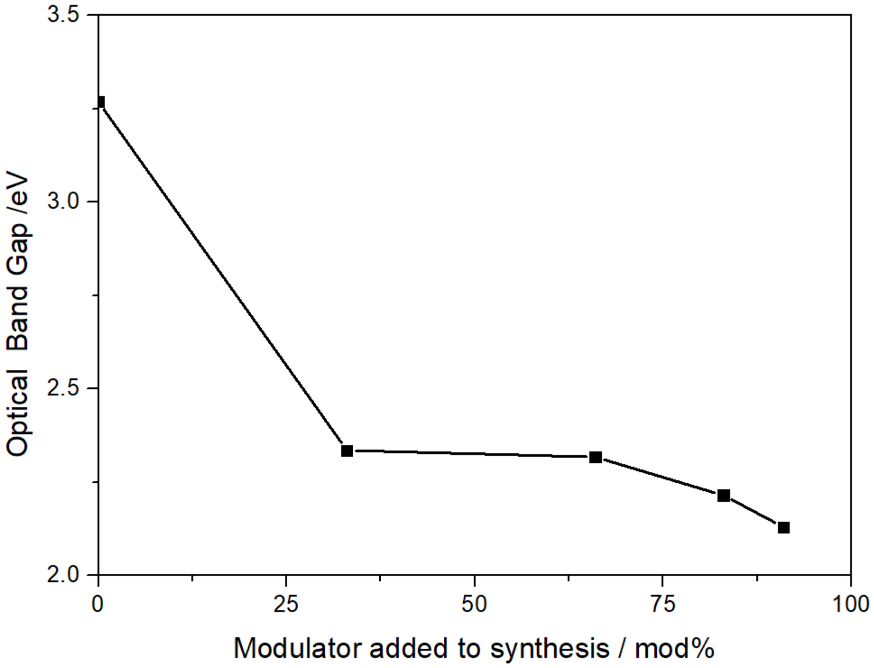

Figure S59: Representation of band gap of MUV-10-OH samples as a function of the molar percent of modulator incorporated into the framework.

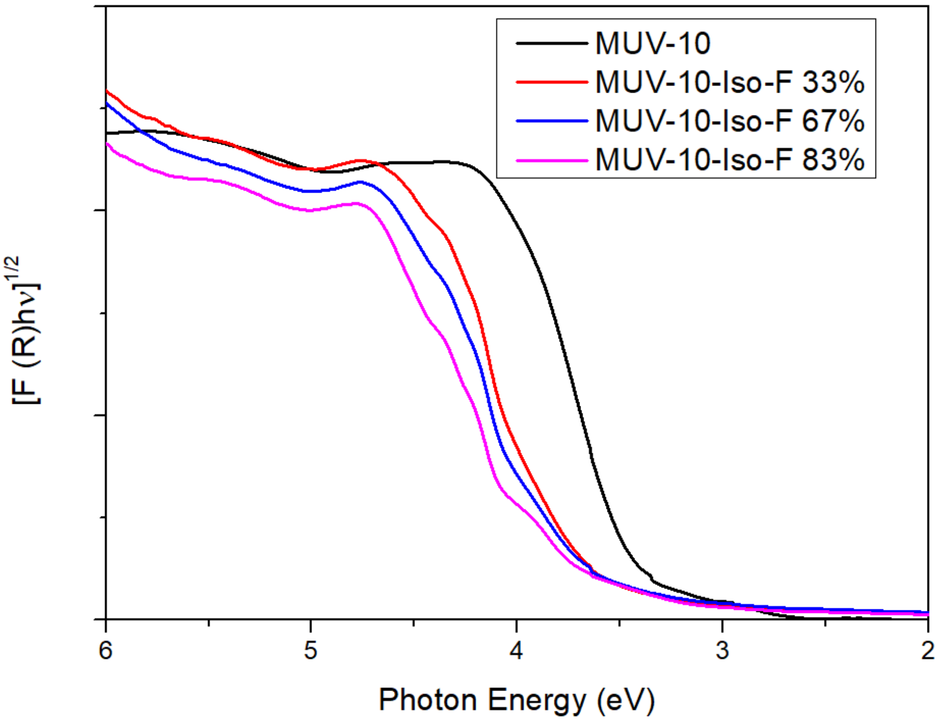

Figure S60: Kubelka-Munk function of MUV-10 and MUV-10-OH samples.

S.5. Transient absorption spectroscopy

Transient absorption spectra were recorded using the forth harmonic of a Q switched Nd:YAG laser (Quantel Brilliant, 266 nm, 15 mJ/pulse, 7 ns fwhm) coupled to a mLFP-122 Luzchem miniaturized detection equipment. This transient absorption spectrometer includes a 300 W ceramic xenon lamp, 125 mm monochromator, Tektronix TDS-2001C digitizer, compact photomultiplier and power supply, cell holder and fibre-optic connectors, computer interfaces, and a software package developed in the LabVIEW environment from National Instruments. The laser flash generates a 5 V trigger pulses with programmable frequency and delay. The rise time of the detector/digitizer is ~3 ns up to 300 MHz (2.5 GHz sampling). The monitoring beam is provided by a ceramic xenon lamp and delivered through a fibre-optic cable. The laser pulse is probed by a fibre that synchronizes the photomultiplier detection system with the digitizer operating in the pre-trigger mode.

$$f(t) = a_1 e^{-b_1 t} + a_2 e^{-b_2 t} \qquad \text{Equation 1}$$

$$f(t) = A e^{-Bt} \qquad \text{Equation 2}$$

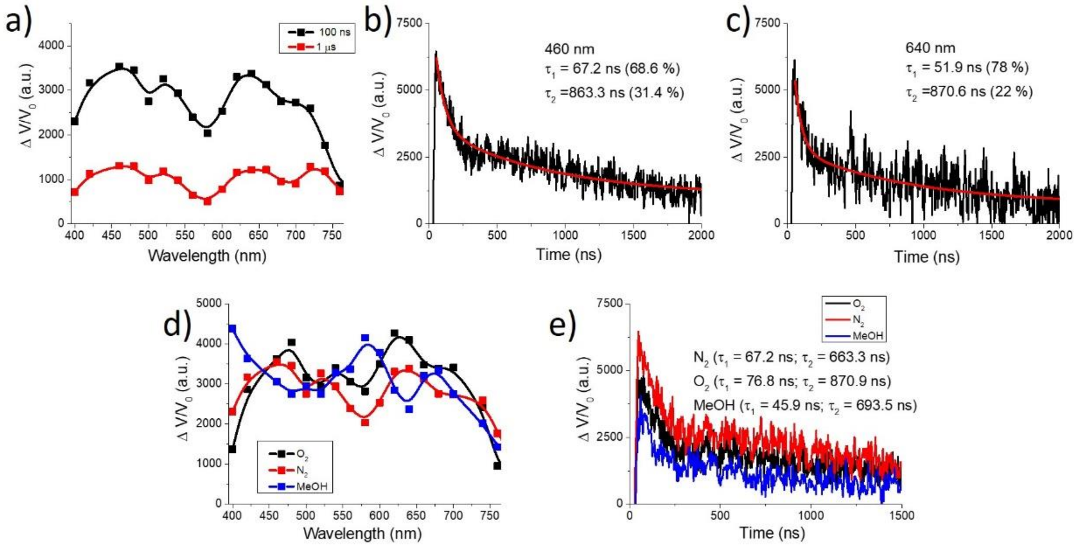

Figure S61: Transient absorption spectrum (a) of MUV-10 dispersion in acetonitrile under N_2 atmosphere upon 355 nm laser excitation. Deactivation kinetics monitored at 460 nm (b) and 640 nm(c). Red lines indicate bi-exponential function fitting. The lifetimes obtained from the fitting are indicated in the insets. Transient absorption spectrum (d) of MUV-10 under N_2 (red), O_2 (red) and MeOH:Acetonitrile mixture 15% v:v in N_2 atmosphere. (e) Transient kinetics monitored at 640 nm under N_2 or O_2 atmosphere and MeOH:Acetonitrile mixture 15% v:v in N_2 atmosphere.

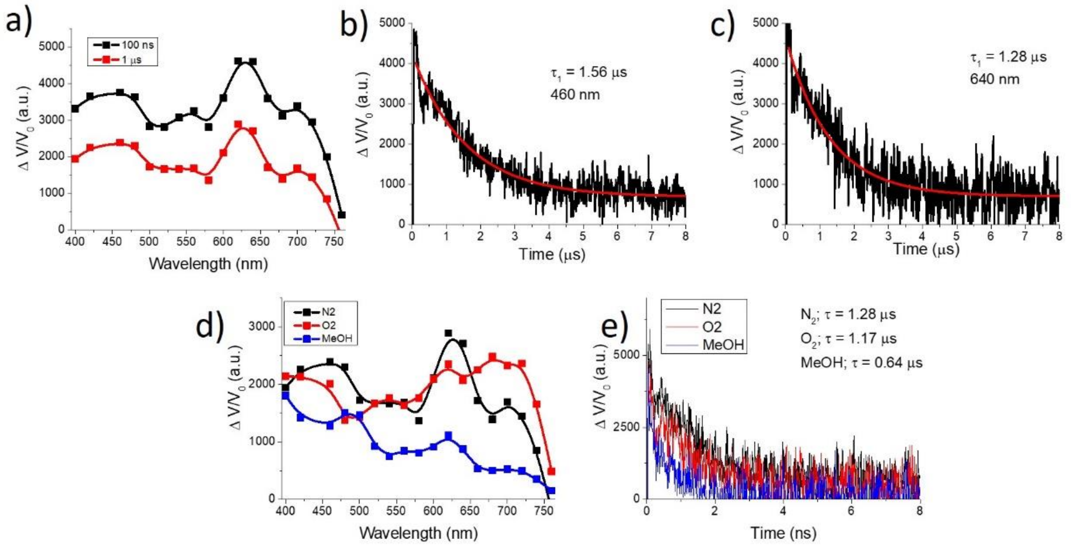

Figure S62: Transient absorption spectrum (a) of MUV-OH(33) dispersion in acetonitrile under N_2 atmosphere upon 355 nm laser excitation. Deactivation kinetics monitored at 460 nm (b) and 640 nm(c). Red lines indicate bi-exponential function fitting. The lifetimes obtained from the fitting are indicated in the insets. Transient absorption spectrum (d) of MUV-OH(33) under N_2 (red), O_2 (red) and MeOH:Acetonitrile mixture 15% v:v in N_2 atmosphere. (e) Transient kinetics monitored at 640 nm under N_2 or O_2 atmosphere and MeOH:Acetonitrile mixture 15% v:v in N_2 atmosphere.

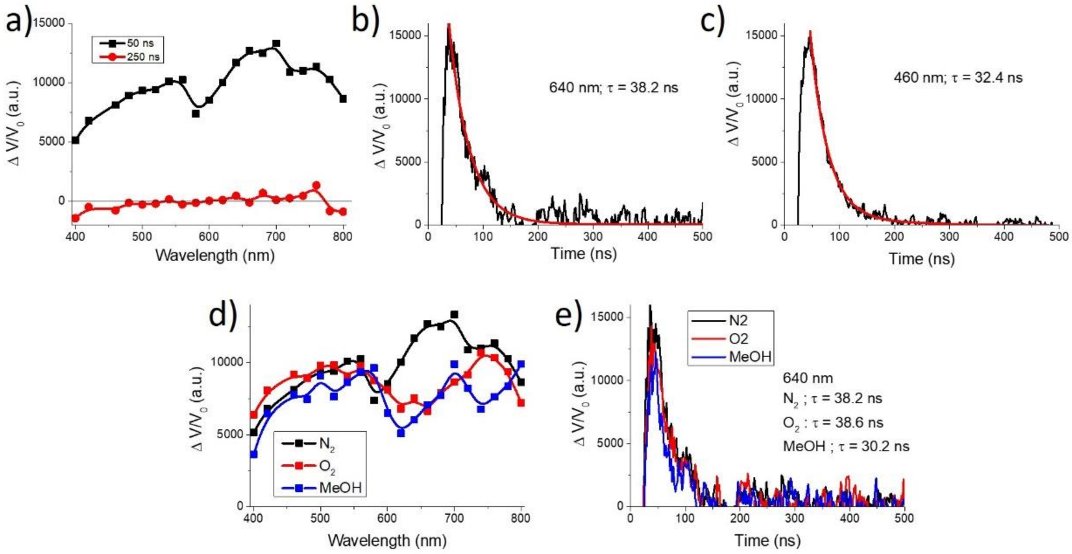

Figure S63: Transient absorption spectrum (a) of MUV-OH(91) dispersion in acetonitrile under N_2 atmosphere upon 355 nm laser excitation. Deactivation kinetics monitored at 460 nm (b) and 640 nm(c). Red lines indicate bi-exponential function fitting. The lifetimes obtained from the fitting are indicated in the insets. Transient absorption spectrum (d) of MUV-OH(91) under N_2 (red), O_2 (red) and MeOH:Acetonitrile mixture 15% v:v in N_2 atmosphere. (e) Transient kinetics monitored at 640 nm under N_2 or O_2 atmosphere and MeOH:Acetonitrile mixture 15% v:v in N_2 atmosphere.

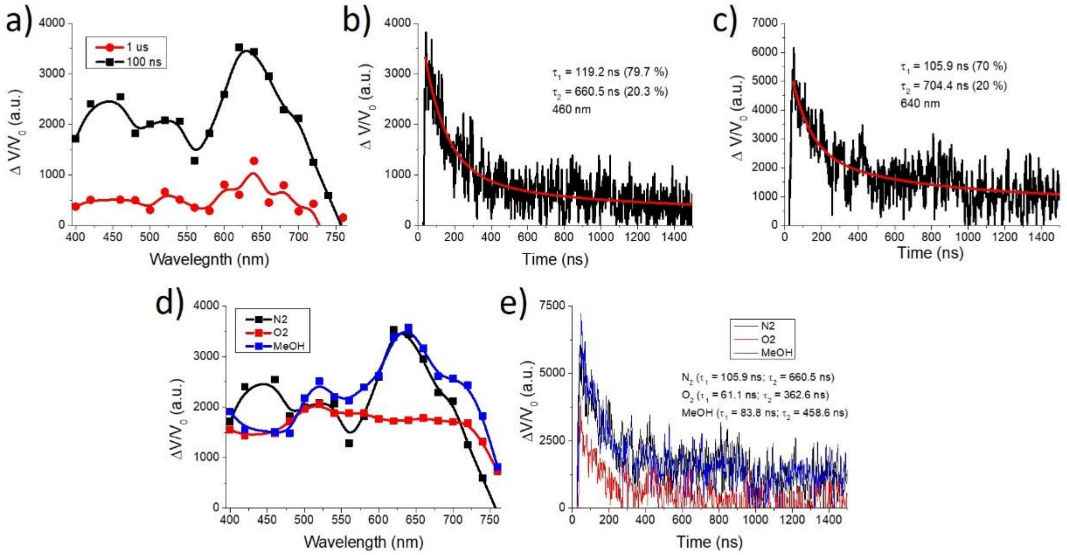

Figure S64: Transient absorption spectrum (a) of MUV-F(33) dispersion in acetonitrile under N_2 atmosphere upon 355 nm laser excitation. Deactivation kinetics monitored at 460 nm (b) and 640 nm(c). Red lines indicate bi-exponential function fitting. The lifetimes obtained from the fitting are indicated in the insets. Transient absorption spectrum (d) of MUV-F(33) under N_2 (red), O_2 (red) and MeOH:Acetonitrile mixture 15% v:v in N_2 atmosphere. (e) Transient kinetics monitored at 640 nm under N_2 or O_2 atmosphere and MeOH:Acetonitrile mixture 15% v:v in N_2 atmosphere.

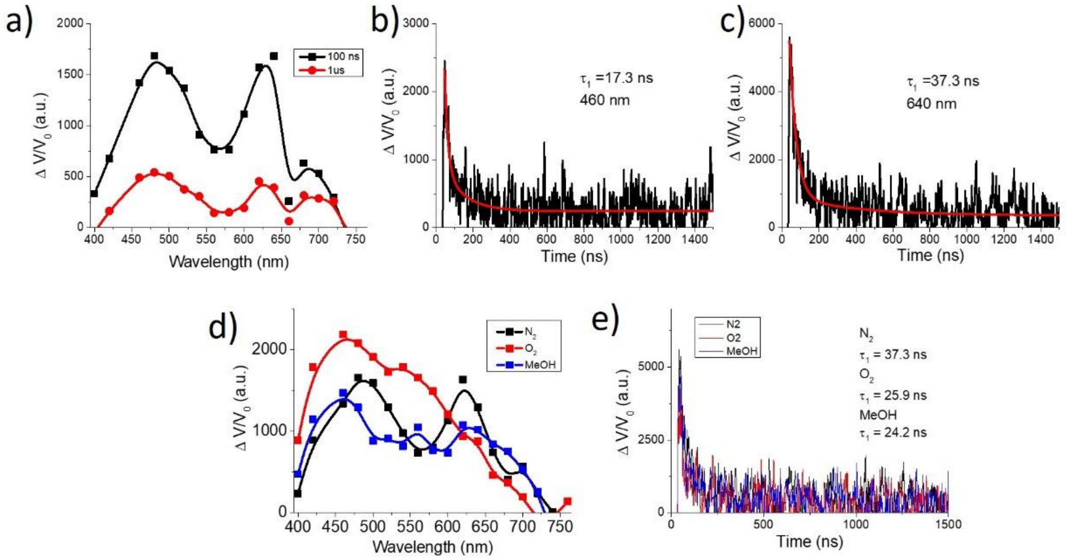

Figure S65: Transient absorption spectrum (a) of MUV-F(91) dispersion in acetonitrile under N_2 atmosphere upon 355 nm laser excitation. Deactivation kinetics monitored at 460 nm (b) and 640 nm(c). Red lines indicate bi-exponential function fitting. The lifetimes obtained from the fitting are indicated in the insets. Transient absorption spectrum (d) of MUV-F(91) under N_2 (red), O_2 (red) and MeOH:Acetonitrile mixture 15% v:v in N_2 atmosphere. (e) Transient kinetics monitored at 640 nm under N_2 or O_2 atmosphere and MeOH:Acetonitrile mixture 15% v:v in N_2 atmosphere.

S.6. References

1 I. A. Lázaro, *Eur J Inorg Chem,* 2020, **2020**, 4284.

2 D. Mateo, A. Santiago-Portillo, J. Albero, S. Navalón, M. Alvaro and H. García, *Angewandte Chemie Int Ed*, 2019, **58**, 17843.
